# The synthesis of some novel stilbene dimers incorporating diamide tethers: studies in single electron transfer oxidation (FeCl_3_)†

**DOI:** 10.1039/c7ra12534h

**Published:** 2018-01-11

**Authors:** Maryam Sadat Alehashem, Azhar Ariffin, Amjad Ayad Qatran Al-Khdhairawi, Noel F. Thomas

**Affiliations:** Department of Chemistry, Faculty of Science, University of Malaya 50603 Kuala Lumpur Malaysia maryam.alehashem@yahoo.com azhar70@um.edu.my noelfthomas@um.edu.my noelfthomas@yahoo.com; Atta-ur-Rahman Institute for Natural Product Discovery, Faculty of Pharmacy, Universiti Teknologi MARA Selangor Branch 42300 Puncak Alam Selangor Malaysia

## Abstract

The FeCl_3_ oxidative cascade reaction of the acetamido stilbene 1 which we reported some years ago produced the first atropodiastereomeric indolostilbene hybrid 3. By contrast, recent investigation of the oxidation of the stilbene succinamide dimer 72 (FeCl_3_/CH_2_Cl_2_) appears, on the basis of spectroscopic evidence, to have produced the bridged macrocyclic indoline 73.

## Abstract 2

Investigation of the oxidation of the stilbene succinamide dimer 72 (FeCl_3_/CH_2_Cl_2_) appears, on the basis of spectroscopic evidence, to have produced the bridged macrocyclic indoline 73.

## Graphical abstract

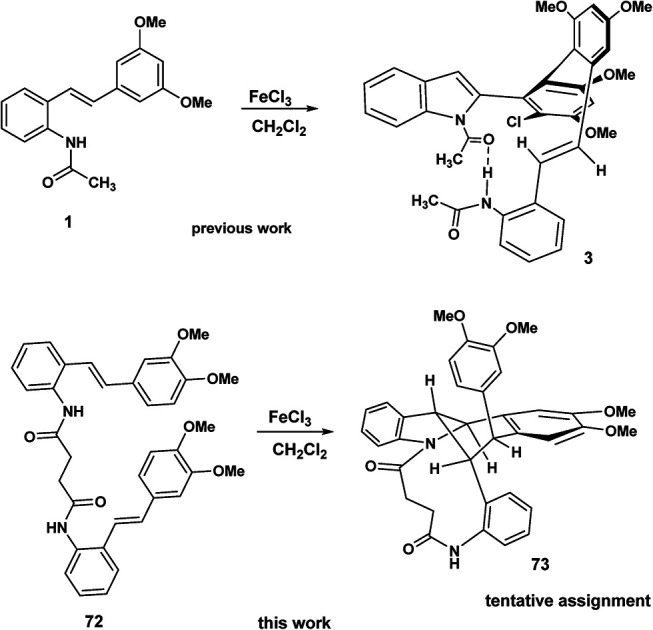

## 1. Introduction

Some years ago we discovered that the 3,5-dimethoxy substituted acetamido stilbene, 1 when exposed to FeCl_3_ in CH_2_Cl_2_ proceeded in a mechanistically complex reaction to yield four products,^1^ one of which was, unprecedented.

**Scheme 1:**
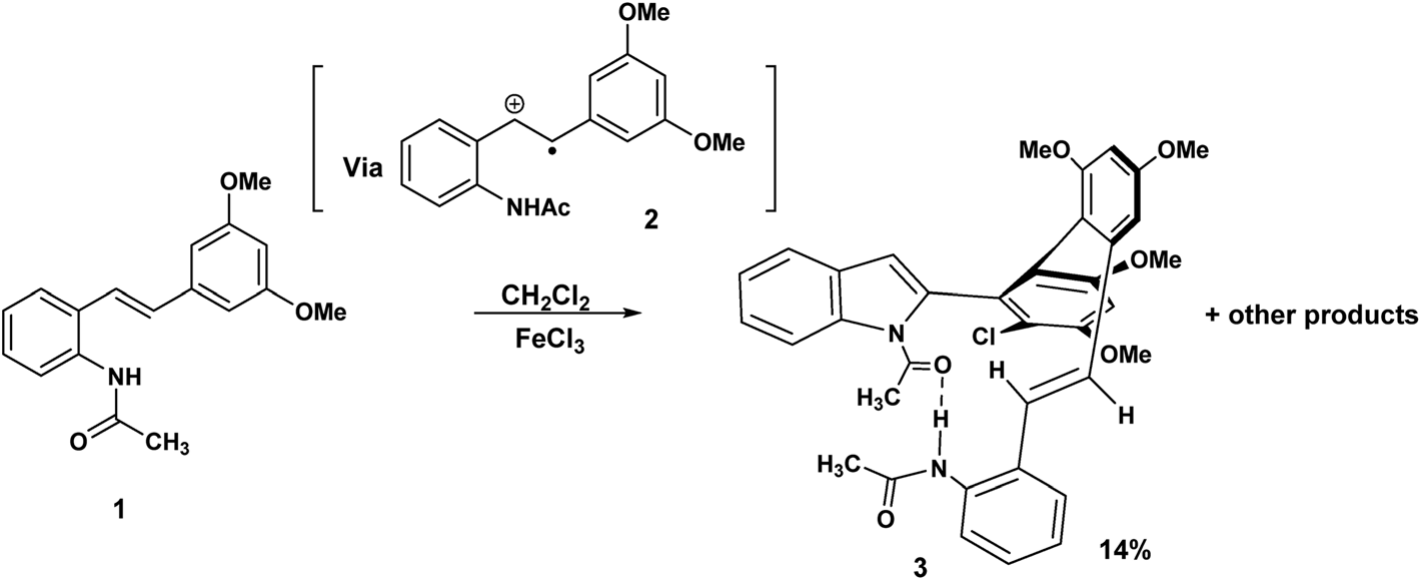
A radical cation mediated synthesis of an indolo stilbene hybrid.

**Scheme 2:**
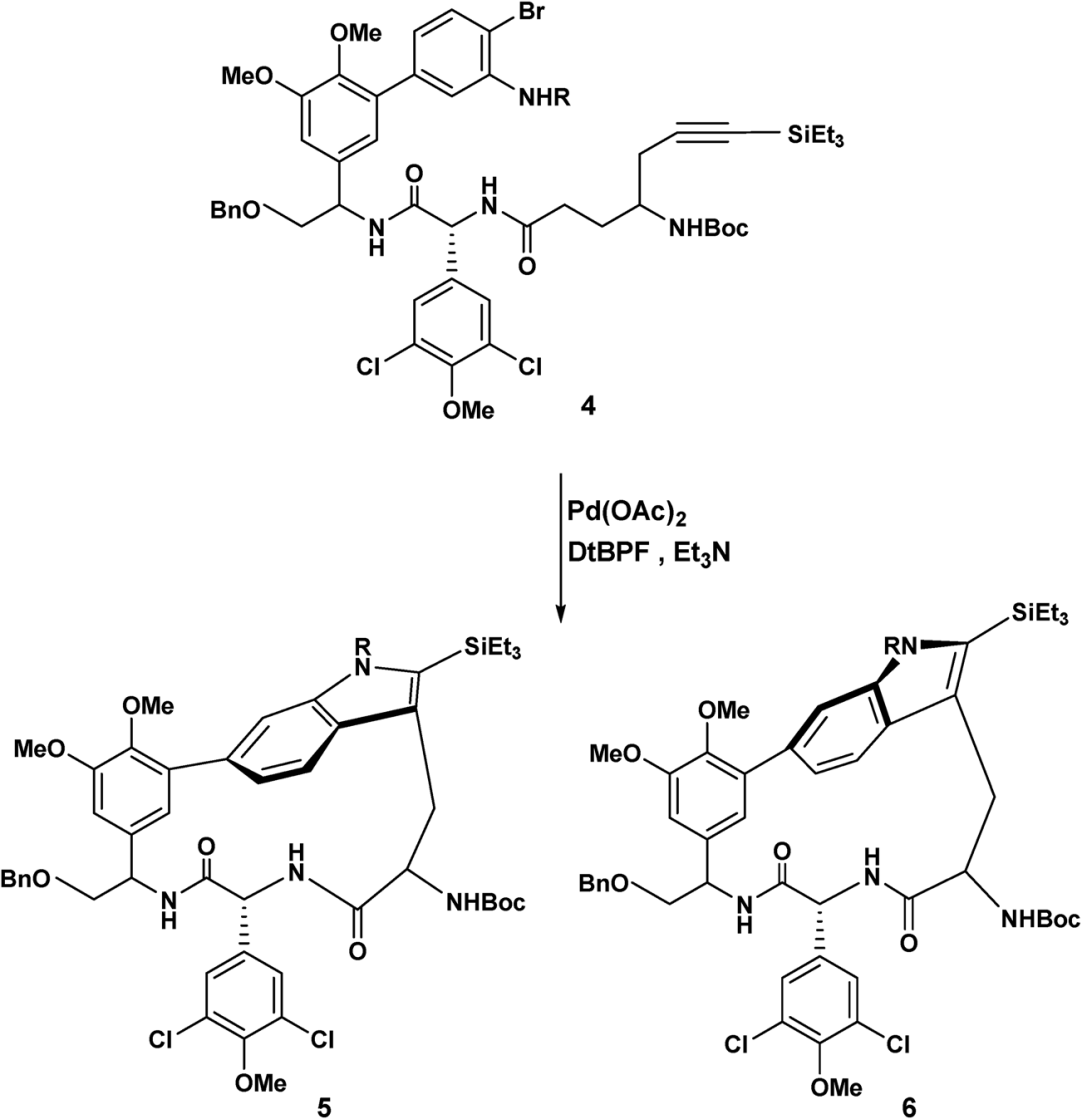
Boger's application of the Larock annulation to macrocyclic construction.

This atropodiastereoselective transformation gave rise to a product 3 incorporating a stilbene, an indole, a chlorodimethoxyphenyl substituent, two stereogenic axes and an intramolecular hydrogen bond resulting in a 14-membered pseudomacrocycle in the conformation shown, 3 (Scheme 1). This development raises the question of the effect of methoxy substitution since completely different products are obtained when the substitution pattern was changed from 3,5 to 3,4-dimethoxy.^2^ The above transformation raises the intriguing possibility that a macrocyclic variant of this reaction may be a realistic possibility. To the best of our knowledge, macrocyclic synthesis *via* oxidatively generated tethered amino stilbene radical cations, has not previously been reported. To put this development into a wider context, Boger reported a powerful and versatile Pd(0) mediated indole macrocyclisation that resulted in the synthesis of chloropentin/DEF molecules^3^ (Scheme 2).

The Boger group noted that free radical and peptide coupling strategies failed^3^ to deliver the macrocycles 5 and 6. For other examples of palladium mediated heterocyclic/macrocyclic construction see the work of Harrowven,^4^ Ohno,^5^ Martin^6^ and Parker.^7^

**Scheme 3:**
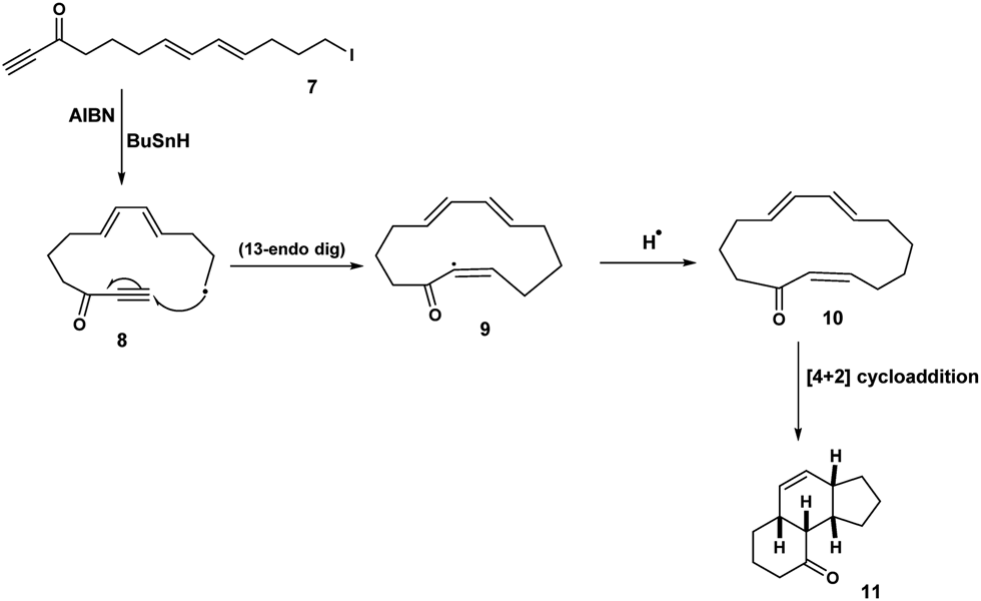
Macrocyclic precursors *en route* Diels Alder reaction.

Some years earlier in a fascinating exploration of the radical-mediated transannular/Diels Alder reaction, Jones^8^ reported that treatment of 7 with tributyltin hydride/AIBN gave rise to the tricycle 11*via* 13-endo dig and Diels Alder reactions (Scheme 3).

**Scheme 4:**
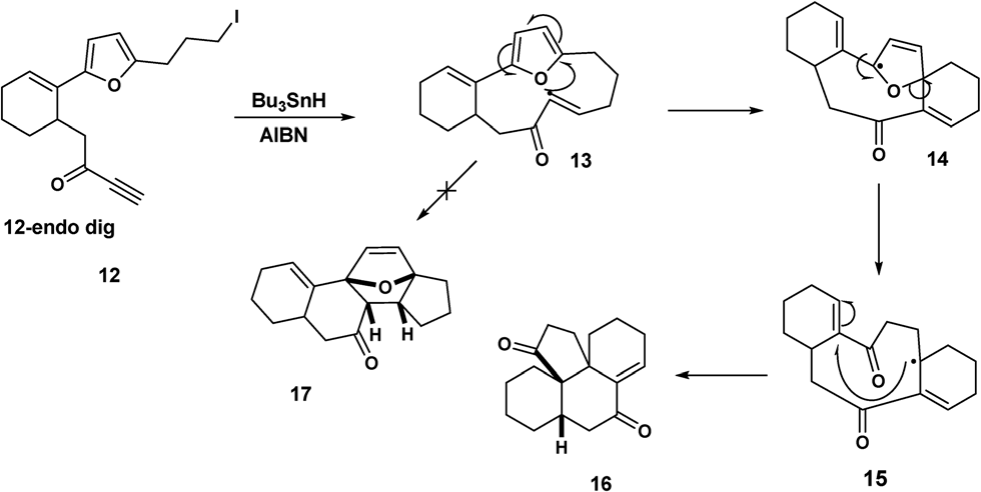
Pattenden's radical mediated synthesis of a tetracyclic ketone *via* macrocyclic precursors.

By contrast, when Pattenden *et al.* treated the iodopropyl furan derivative 12 with tributyltin hydride, the intermediate formed by 12-endo dig ring closure underwent furan cleavage 14 followed by 5-exo trig ring closure 15 to yield the tetracyclic ketone 16, Scheme 4.

**Scheme 5:**
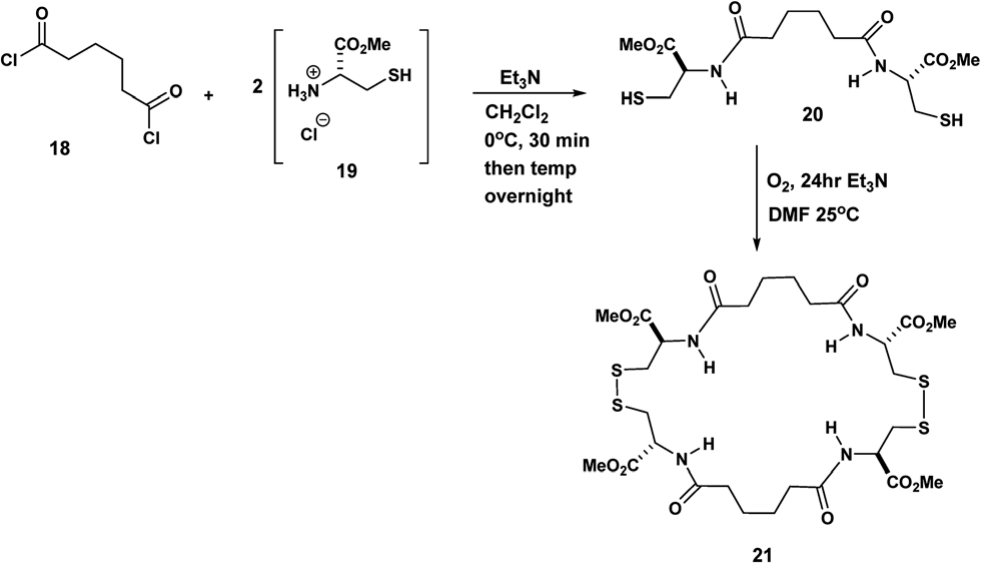
Exploitation of adipoyl tethered cysteines in macrocyclic disulphide synthesis.

The Pattenden syntheses above generated macrocyclic intermediates which are an efficient means to the desired ends that is, the macrocycles are not the final products. By contrast in the next few examples, the macrocycles are the intended targets. For example in the synthesis by Endo, the macrocyclic final products incorporate diamide tethers. Endo,^9^ linked two molecules of l-cysteine 19 by means of adipoyl chloride 18. The corresponding diester diamide was exposed O_2_ in Et_3_N/DMF at 25 °C to yield the bisdisulphide macrocycle, 21 (Scheme 5).

The concept of the tethered stilbene has been illustrated by the work of Mizuno.^10^ Parabromobenzaldehyde 22 was exposed to TiCl_4_/Zn to yield the paradibromo stilbene 23 by McMurry coupling. Treatment of 23 with *t*-BuLi gave the dilithiated species (24) which then reacted with 1,3-bis[(chlorodimethylsilyl)methyl]benzene 25 to produce the *trans*,*trans* stilbenophane 26. Photoreaction of 26 gave rise to the tethered cyclobutane macrocycles^10^27 and 28. Again to the best of our knowledge, no stilbene dimers incorporating diamide ethers based on succinamide or adipoylamide have been reported and studied from the point of view of FeCl_3_ promoted cyclisations. This brings us to the next section where this novel concept is described (our synthetic plan).

## 2. The synthetic plan

**Scheme 6:**
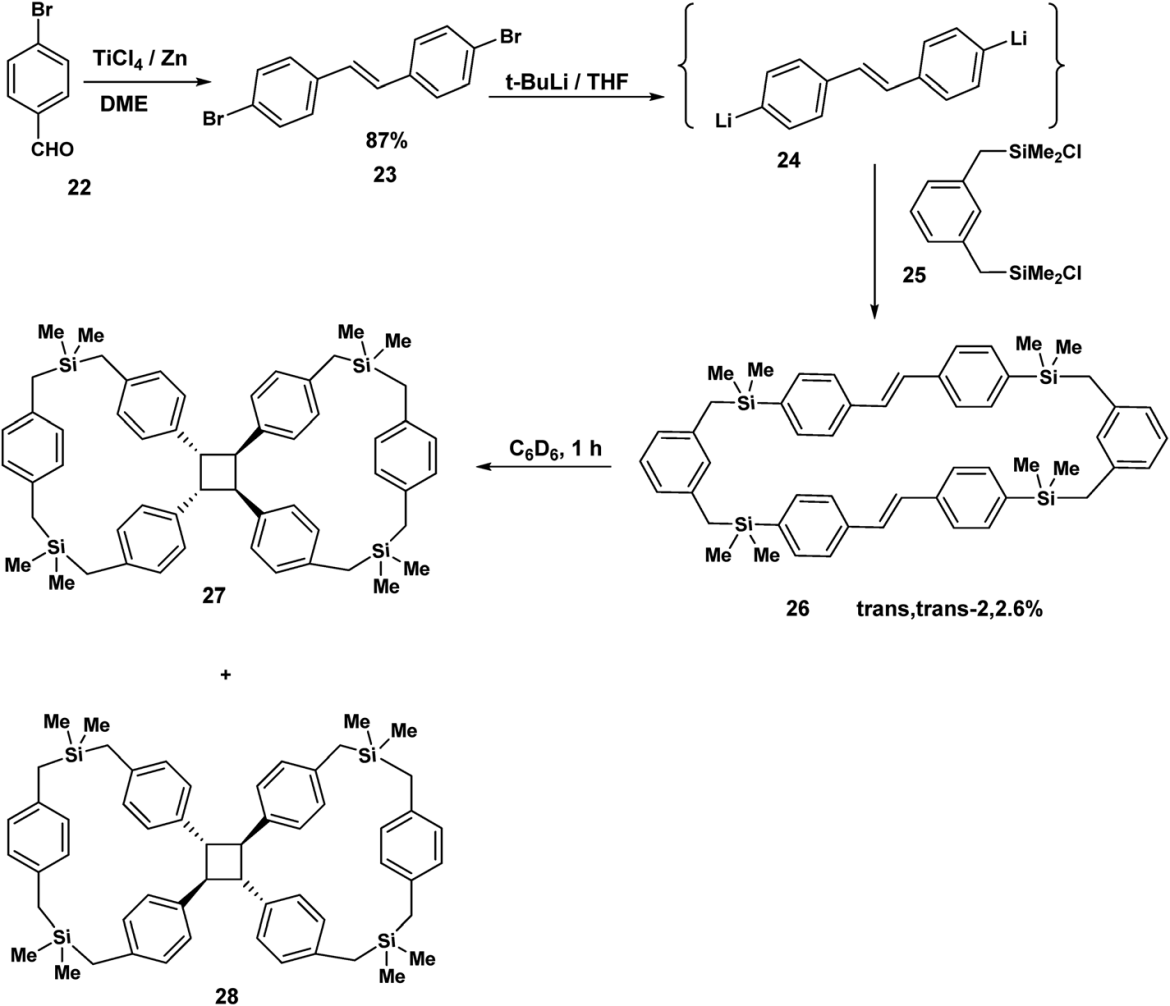
Stilbenophanes (generated by the McMurry reaction) transannular precursors of the cyclobutane macrocycles.

**Scheme 7:**
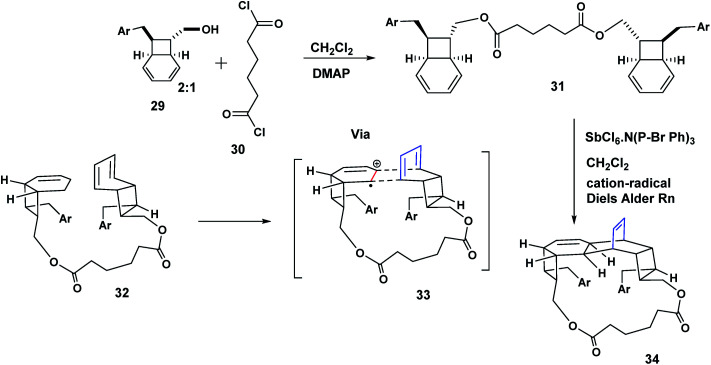
Parker's use of an adipoyl tether for an intramolecular cation radical Diels Alder reaction.

The Mizuno study we have just described (Scheme 6) established the principle that two stilbenes could be held together by a silane tether in preparation for cycloaddition. Our confidence in viability of our diamide tethered Heck precursor strategy was further encouraged by the report of Parker^11^ in which the bicyclo-[4.2.0]-octadiene 29 was prepared in racemic form in seven steps. Treatment of 29 with adipoyl chloride, DMAP, CH_2_Cl_2_, gave the two adipoylamide octadiene dimers; a racemic modification and the C-2 symmetric compound 31. By way of illustration, when the racemic compound was exposed to the antimony hexachloride complex (a cation radical salt), a pentacyclic bislactone macrocycle 34 was obtained. This bis lactone was eventually transformed to the natural product (+)-Kingianin (A).^11^ The literature reveals many elegant applications of the cation radical Diels Aleder reaction^12–17^ (Scheme 7).

**Scheme 8:**
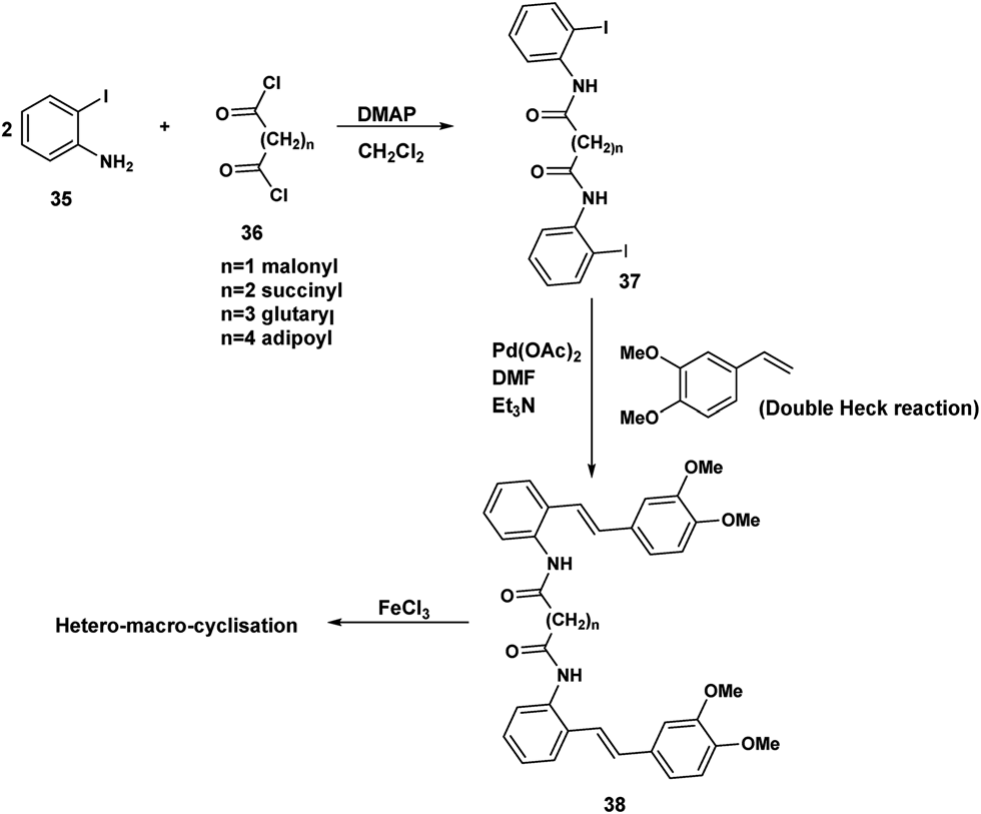
Our synthetic plan for the preparation of diamide tethered stilbenes.

We believed we could adopt diacid chlorides like 30 to prepare our novel dimers as described below. Our basic plan is shown in Scheme 8.

## 3. Results and discussion

**Scheme 9:**
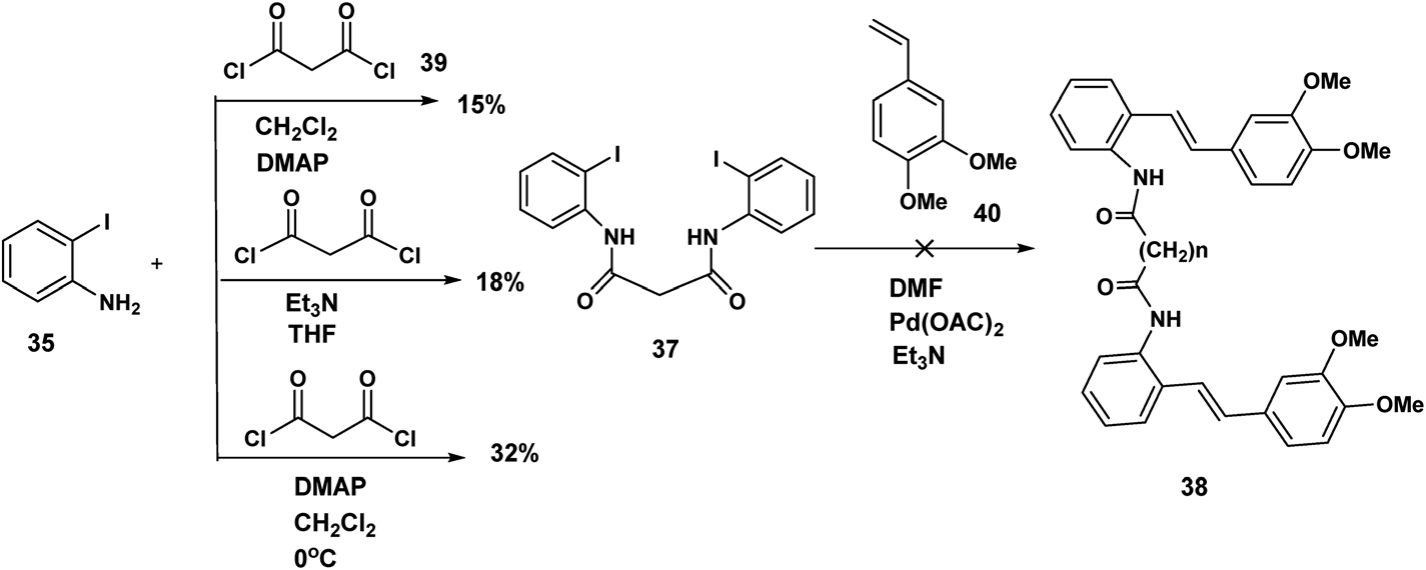
Attempted double acylation/double Heck.

Although the iodophenyl malonamide dimer 37 could be prepared in moderate yields (Scheme 9), for reasons we will revisit later, the attempted double Heck coupling proved to be disappointing. We surmised that the coupling of the palladium reagent to the two amide carbonyls to give a six membered chelate inhibited the oxidative insertion step in the Heck reaction. To the extent that the presence of two NH'S contributes to this problem, protection of both amide nitrogen by the SEM protecting group in addition to changing both the palladium reagent and the inclusion of a ligand would improve the efficiency of the Heck coupling probably by promotion of the all important oxidative insertion into the aryl indole bond. An alternative reagent combination^18^ Pd_2_(dba)_3_/Pd(*t*-Bu_3_P)_2_ could have been tried. These alternatives notwithstanding, we opted for a revised strategy. In this approach the amino stilbene monomer was constructed by the Heck procedure followed by a double acylation with the appropriate diacid chloride. This revised procedure is depicted below.

The optimum conditions for the Heck, in contrast to our previous experience with various ortho amido stilbenes,^19^ was to dissolve the iodoaniline 35 in dry CH_3_CN under nitrogen with heating to 80 °C. Palladium acetate, Et_3_N and 2-furyl phosphine^20^ were then added in this order followed by 3,4-dimethoxystyrene 40. Refluxing continued until the starting material was consumed. Similar conditions were exploited by Heck in the course of a synthesis of a more functionalized version of our amino stilbene.

The double acylation reaction proved to be much more challenging. In our early attempts malonyl chloride was added dropwise to a solution of the amino stilbene in dry CH_2_Cl_2_. This led to a complex mixture from which, after chromatography, only 22% of the desired product could be isolated. The complications associated with preparing amides by the acid chloride procedure are by now well known as the extensive the peptide and related literature clearly indicate. Ketene formation and associated side reactions are to be expected. Although many ingenious methods for tacking these problems by activation of the corresponding carboxylic acids, have been developed, we found that when the amino stilbene was added slowly dropwise to malonyl chloride for 1 h at 0 °C, we obtained the best yield of the corresponding dimer (65%). By means of this improved procedure the corresponding dimers incorporating succinyl (*n* = 2), glutaryl (*n* = 3) and adipoyl amides (*n* = 4) tethers were prepared in comparable yields.

### Mechanistic hypotheses/predictions dependent on radical cation reactive conformations

**Scheme 10:**
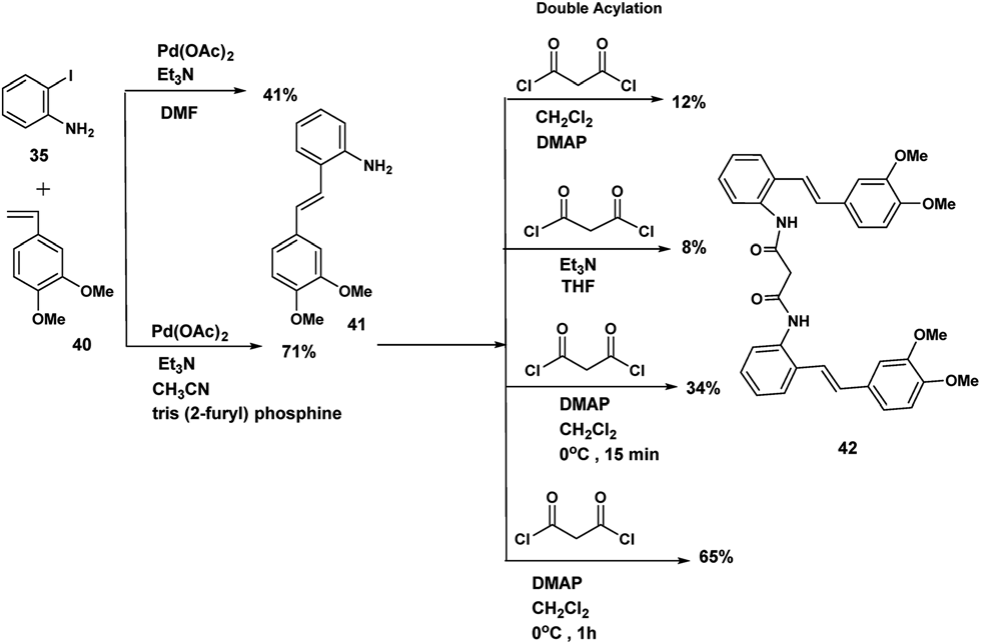
Heck coupling/double acylation.

In Scheme 8, we indicated that 38 when exposed to FeCl_3_ would be expected to undergo polycyclisation/macrocyclisation. The text “carbon-centred free radicals and radical cations” edited by Forbes^21^ in worthy of careful study. We have described the chemistry of radical cations in a review entitled: the radical cation mediated cleavage of catharanthine leading to the vinblastine type alkaloids: implications for total synthesis and drug design.^22^ Radical cation/intact olefin and diradical cation pathways (assuming oxidation of both olefinic bonds) need to be considered (Scheme 10).

**Scheme 11:**
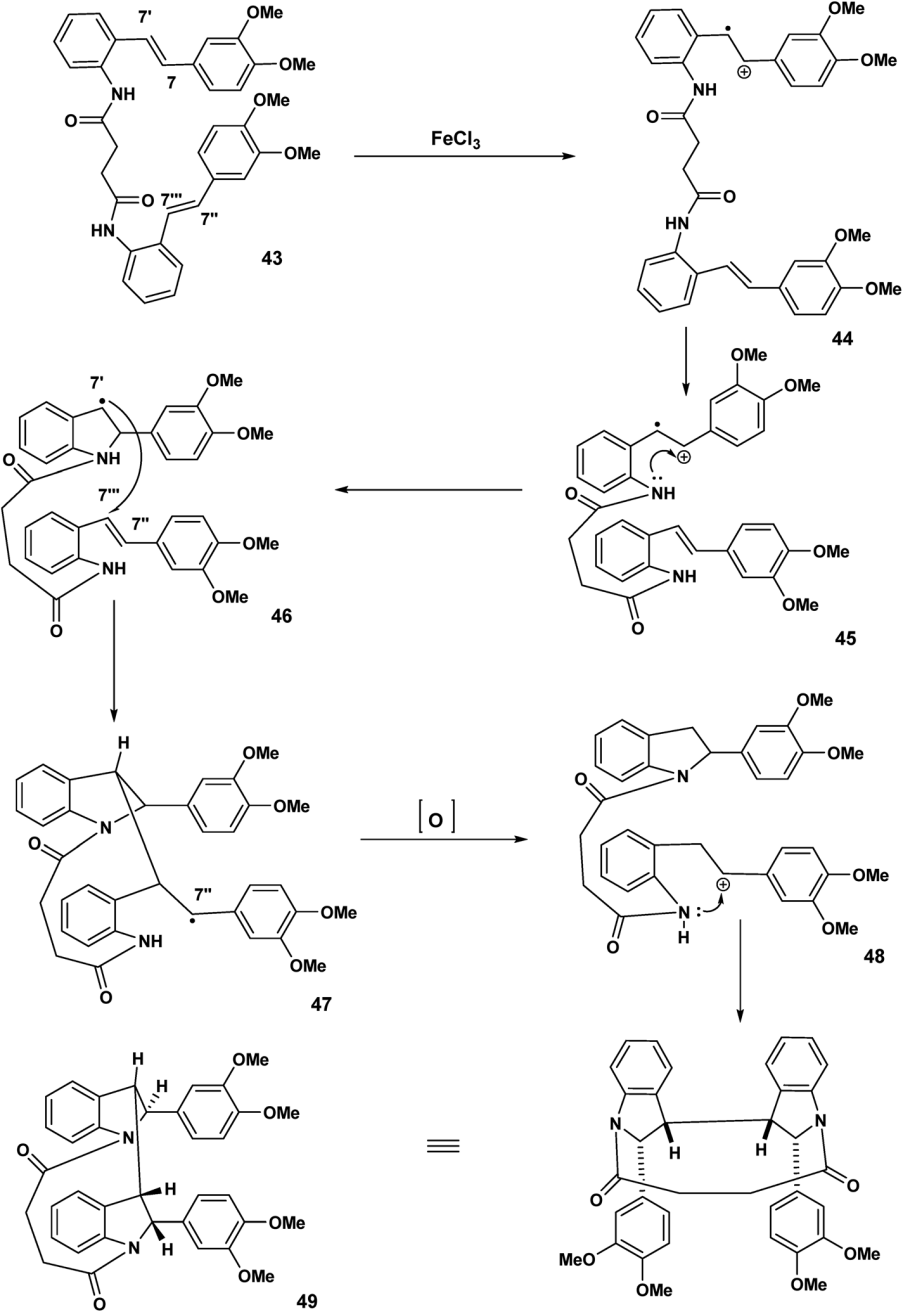
Intramolecular attack of a radical cation on an intact olefin.

In the first proposed pathway, the stilbene 43 has undergone single electron transfer oxidation of one of the olefinic bonds to yield the monoradical cation 44. Intramolecular nucleophilic attack by the NH 44 will give rise to the indolyl radical 46 which subsequently attacks the intact olefin at the C-7′′′ position to yield the C-7′′ macrocyclic radical 47 which undergoes oxidation to the corresponding benzylic carbocation and a second intramolecular capture should result in the bisindoline 49, Scheme 11.

**Scheme 12:**
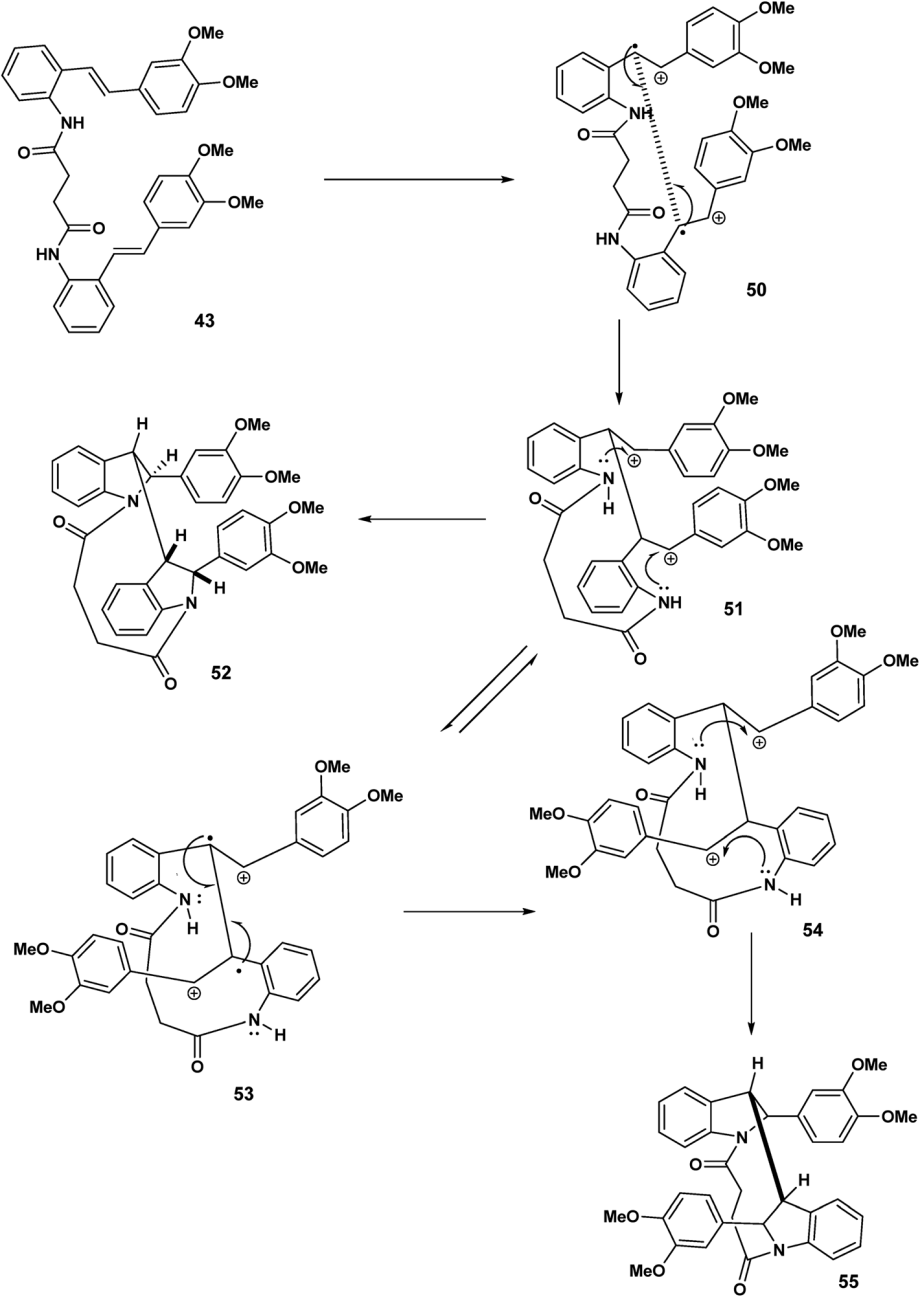
Formation of meso and racemic bisindolines *via*51 and threo 54 dicationic intermediates.

Our speculation regarding diverse pathways for our tethered stilbene radical cation continues in Scheme 12. The key intermediate in this case the diradical cation 50 resulting from oxidation of both olefinic bonds. In intermolecular variants of amino stilbene (or amido stilbene) oxidative dimerisation under electrochemical conditions, a high concentration of the stilbene radical cation is believed to be present on the electrode surface.^23,24^ Under conditions of ferric chloride oxidation, radical cation intact olefin dimerisations would appear to be reasonable given the presumption that the radical cation is in a low concentration although this is not a hard and fast rule. In any event we are in this report dealing with tethered stilbenes and therefore it would prudent to keep an open mind. With the formation of the diradical cation 50 (Scheme 12) radical combination (assumed to precede cyclisation) would give rise to the dication species 51. Double intramolecular capture of the dication will yield either the mesobisindoline 52 from the erythro intermediate 51 or alternatively, the racemic bisindoline 55*via* the threo dicationic intermediate 54 (Scheme 12).

**Scheme 13:**
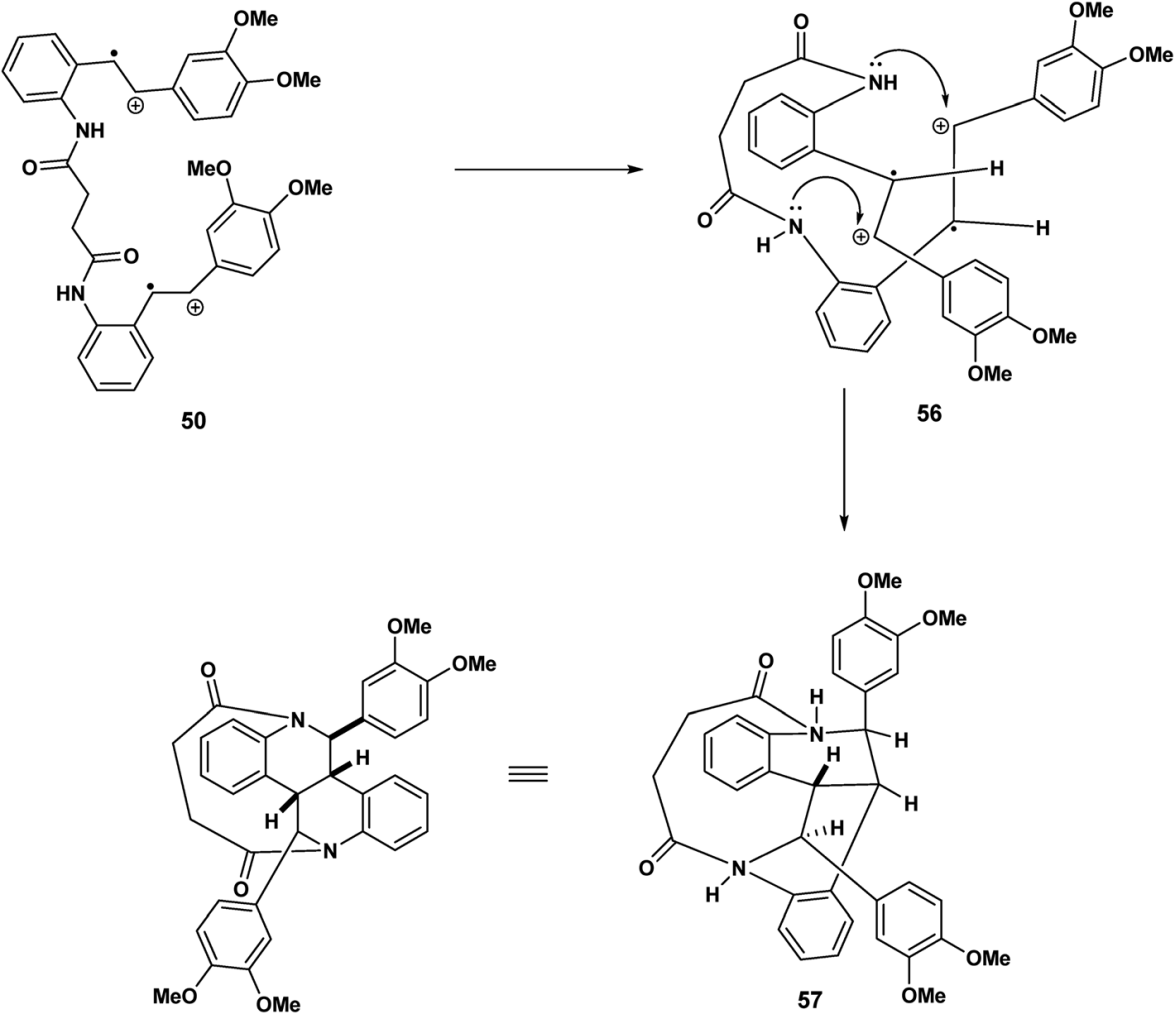
Double intramolecular (cross over capture) of dicationic species 56.

**Scheme 14:**
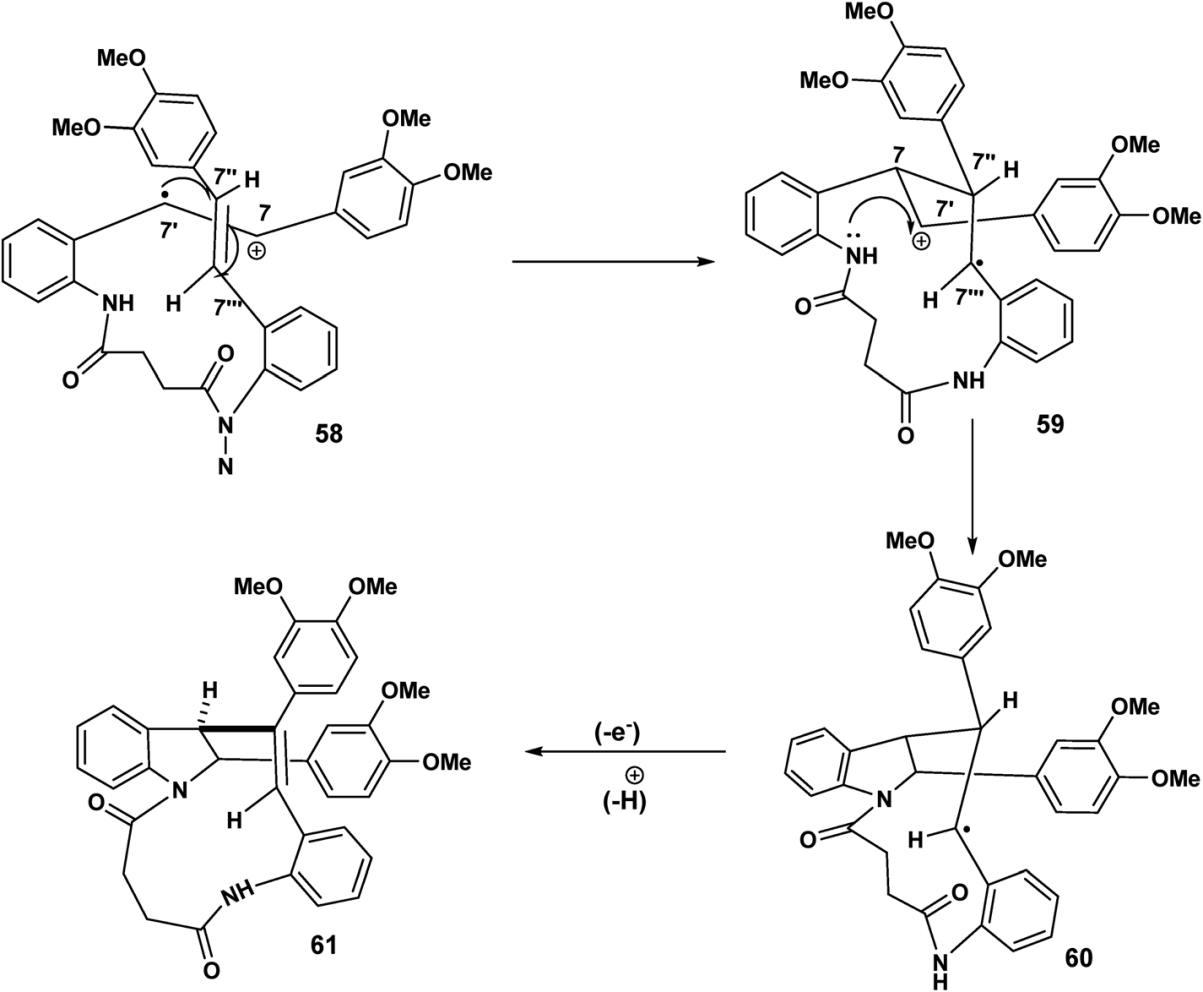
12-endo trig (for *n* = 1) cyclisation of the C(7′) radical culminating in the indolostilbene 61.

Continuing our mechanistic speculation, we see in Scheme 13 the intramolecular example of crossover nucleophilic capture, the intermolecular variant of which we have described previously.^25^ The tethered dication must adopt a reactive conformation that prohibits direct nucleophilic capture on the benzylic carbenium ions 56 of the type described in Scheme 12. The “macrocyclic” bisisoquinolines with cis ring fusion 57 would be the result (Scheme 14).

**Scheme 15:**
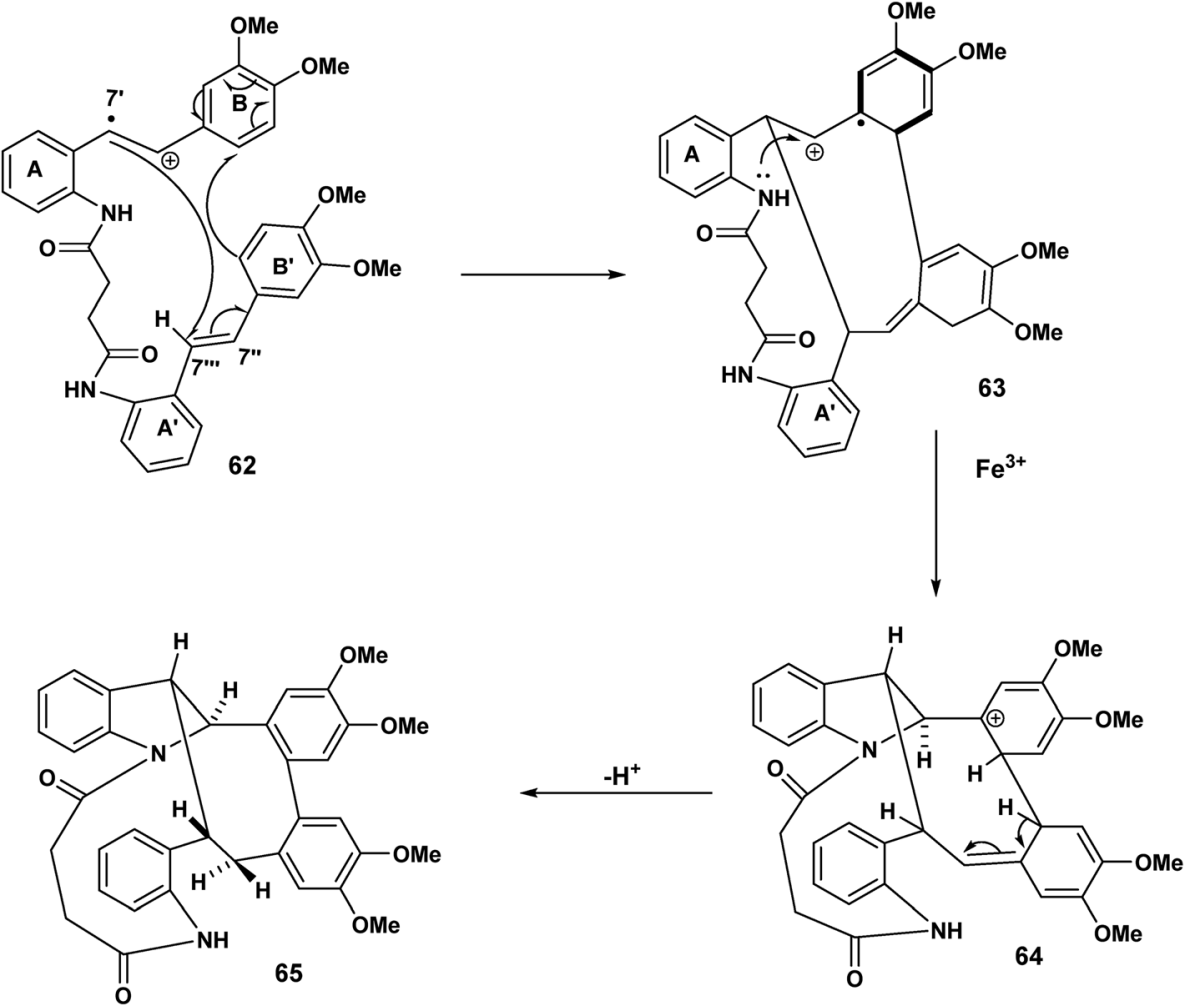
11-endo trig for (*n* = 1) cyclisation of the C(7′) radical biaryl formation.

In Scheme 14, we see another example of radical cation intact olefin dimerisation. Intramolecular attack by the radical 58 on the intact olefin results in a second benzylic radical [59 to 60] after intramolecular capture of the benzylic carbocation 59 (Scheme 14). Further oxidation and deprotonation would proceed to yield the indolostilbene hybrid 61. Any strain inherent in such a structure would be alleviated for higher values of *n* (*e.g.* 3, 4 *etc.*). A different reactive conformation is depicted in Scheme 15 [compare 62 (Scheme 15) with 58 (Scheme 14)]. In this alternative the radical cation, through the of the C-7′ radical, attacks the intact olefin. This sets up the a biaryl link as a result of delocalisation C(7′′) radical (not shown) (Scheme 15). Oxidation and deprotonation gives rise to the bridged fused 8 : 10 ring system incorporating an indoline moiety 65.

**Scheme 16:**
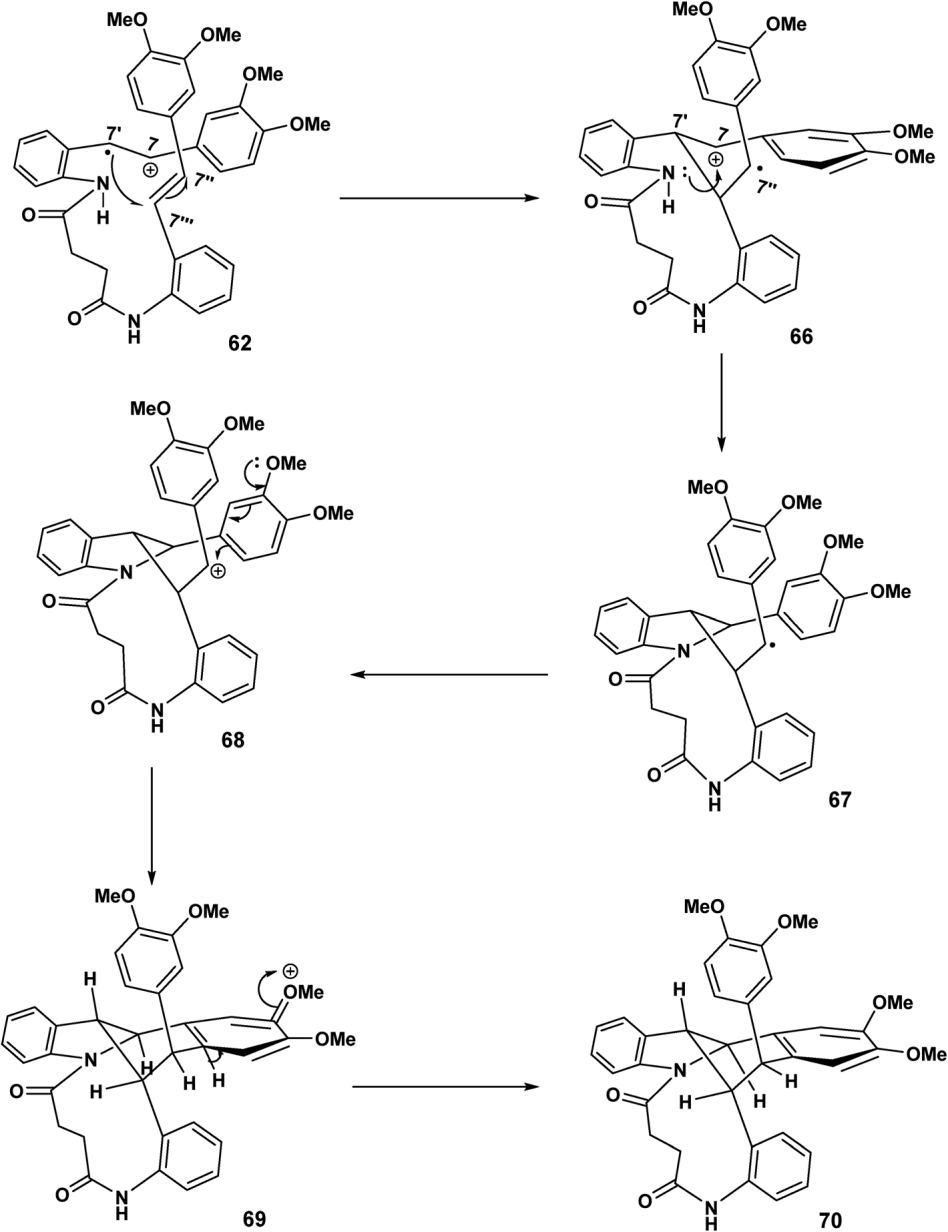
11-endo trig (*n* = 1) ring closer, oxidation, electrophilic aromatic substitution leading to 70.

The cascade sequence depicted Scheme 16 is in contrast to Scheme 15. In the earlier scheme the C(7′) radical 62 underwent delocalisation and subsequently biaryl coupling 63. By contrast (Scheme 16), the C(7′′) radical undergoes rapid oxidation [67 to 68] to yield the carbocationic intermediate 68 which undergoes intramolecular electrophilic aromatic substitution 68 and rearomatisation leading to 70.

### Preliminary reflections on the FeCl_3_ promoted cascade reactions

When the aminostilbene succinamide 72 was exposed to FeCl_3_ (in a 1 : 1 molar ratio) in CH_2_Cl_2_, 73 was obtained as the major product. A comparison of the NMR spectroscopic features of starting material and this product is intriguing.

**Scheme 17:**
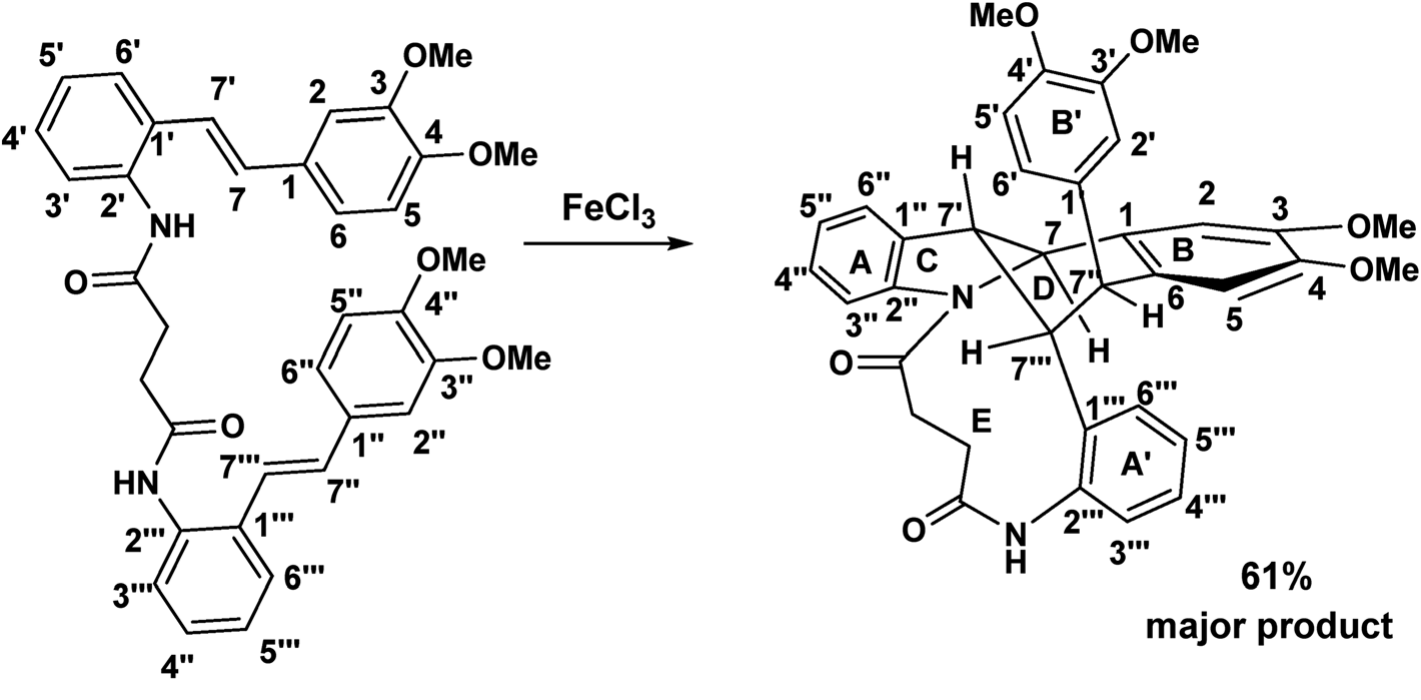
A bridged macrocyclic indoline diamide.

**Fig. 1:**
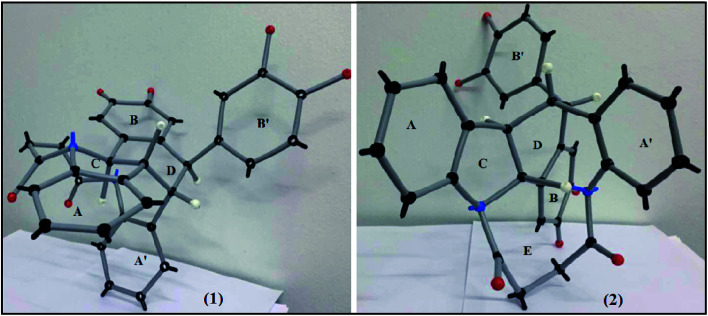
Two views of the physical model of 73.

In examining the aromatic region of 400 MHz NMR spectrum of the proposed structure 73 (Scheme 17) we note that the overlapping pair of doublets, integrating for three protons, at 6.48 ppm correlate with triplets at 7.18 and 7.3 ppm according to the COSY spectrum. The *J* value of 10.32 Hz is consistent with the ortho coupled C(3′′′) C(4′′′) and C(6′′′) and C(5′′′) protons of the A′ ring. The *J* value of 10.3 Hz is slightly outside the normal range for such protons it is interesting to note that the C(6′′′) and C(3′′′) protons of the A′ 73 have experienced much greater shielding compared to the starting succinamide 72 and the corresponding protons of the A ring of the dimer. This is because the C(6′′′) proton experiences a repulsive interaction with the C(7′′′) methine proton (as the physical model reveals) and the C(3′′′) proton is in close proximity to the methylene protons of the succinamide tether (Fig. 1). On the other hand the physical model indicates a more congested environment for this A′ ring system compared with the A ring system (and compared to the starting stilbene succinamide dimer 72) which accounts for the unusual shielding of the C(6′′′) and C(5′′′) protons. In the case of the latter, we observe a second set of doublets at 6.85 and 6.9*δ* which also correlate with the set of triplets in the region 7.27 to 7.4*δ*. These peaks are more deshielded with coupling constants of 7.24 and 7.28 Hz (more normal values). This is consistent with a less crowded environment for the A ring system. Overall these correspond to the doublet–triplet–triplet–doublet pattern represented by the A and A′ ortho substituted aniline ring systems. We believe that the correlating doublets at 6.45 and 6.75 ppm correspond to the protons on the B′ ring system *i.e.* C(5′) H and C(6′) H (a classic AB pattern). It is worth noting that there are three broad aromatic singlets at 6.78 and 6.94 ppm with one more hidden under the broad doublet at 6.5 ppm which integrates for 3 protons. By of comparison, the starting stilbene succinamide dimer 72 has a doublet at 6.87 ppm corresponding to C(2)–H. By contrast for the product 73 there are two broad singlets (at 6.94 and 6.78 ppm) with the third singlet we believe to be hidden under the broad doublet at 6.48 ppm. These singlets correspond to C(2)–H, C(5)–H and C(2′)–H. We now examine the aliphatic region of the spectrum. The four methoxy singlets that cover the region 3.77 to 3.96 ppm indicate the disruption of symmetry in the product 73 compared to the starting material 72 (Scheme 17). It is noteworthy that in the ^13^C spectrum of 73, the four CH_3_ (carbons) are clearly visible and partially overlapping at 55.05, 55.99, 55.86 and 55.93 ppm. From an examination of the HMQC and ^13^C DEPT spectrum. This suggests a disruption of symmetry which is all the more evident the ^13^C data is compared with that of the starting aminostilbene malonamide 72 where the four methoxymethyl carbons overlap to a degree. It is interesting that we have two distinctive singlets at 4.2 and 3.4 ppm. We believe that these singlets correspond to the C(7′′′)–H and C(7′′)–H protons located at two of the four contiguous asymmetric centres. The virtually orthogonal relationship of the protons at C(7′′)–H, C(7′′′)–H and C(7′)–H accounts for the appearance of the C(7′′′)–H and C(7′′)–H protons as singlets. We would expect the C(7′)–H and C(7)–H protons to appear as doublets. We suspect that these protons are subsumed under the broad peak at 2.06 ppm. This broad peak partly masks a small impurity. These features are consistent with our proposed structure and account for the at first surprising upfield shift of these protons. We now turn our attention to the ^13^C spectrum with respect to the methine carbons corresponding to C(7′′′) and C(7′′). There carbons in the HMQC are found at 47 and 50 ppm respectively. These peaks relate to the proton spectrum where C(7′′) is at 4.2 and C(7′′′) at 3.4 ppm. C(7′′) is clearly more deshielded because of the electron withdrawing effect of the B and B′ aromatic rings. The other two methine carbons can be found under the region 26 to 28 ppm and are thus related to the corresponding protons under the broad peak at 2.06 ppm (from the HMQC) (the carbon 13 peaks are admittedly weak and impurities in that region complicate matters but the HMQC places these methine carbons at about 27 ppm). It is significant that the C(7) and C(7′) methine protons are in more sterically congested environments due on the one hand, to the environment of the succinamide moiety and the A′ ring and on the other, the B′ ring in a pseudo 1,3-diaxial interaction with the C(7′) proton (there is of course another pseudo 1,3-diaxial interaction between the C(7) proton and the A′ ring). This explains the upfield shift of these methine protons in the proton spectrum. Notice that the broad peak we have mentioned at 2.06 ppm most likely includes the N–H which is more shielded compared to other systems we have studied. The succinyl protons that appeared as a sharp peak (singlet) integrating for four protons at 2.87 ppm in the starting stilbene succinamide dimer, now appear as a broad multiplet at 3.18 in 73. The broad peak at 2.06 ppm integrates for three protons (the C(7), C(7′) and NH protons). These intriguing features among others are a consequence of conformational effects inherent in the bridged macrocyclic super structure.

**Fig. 2:**
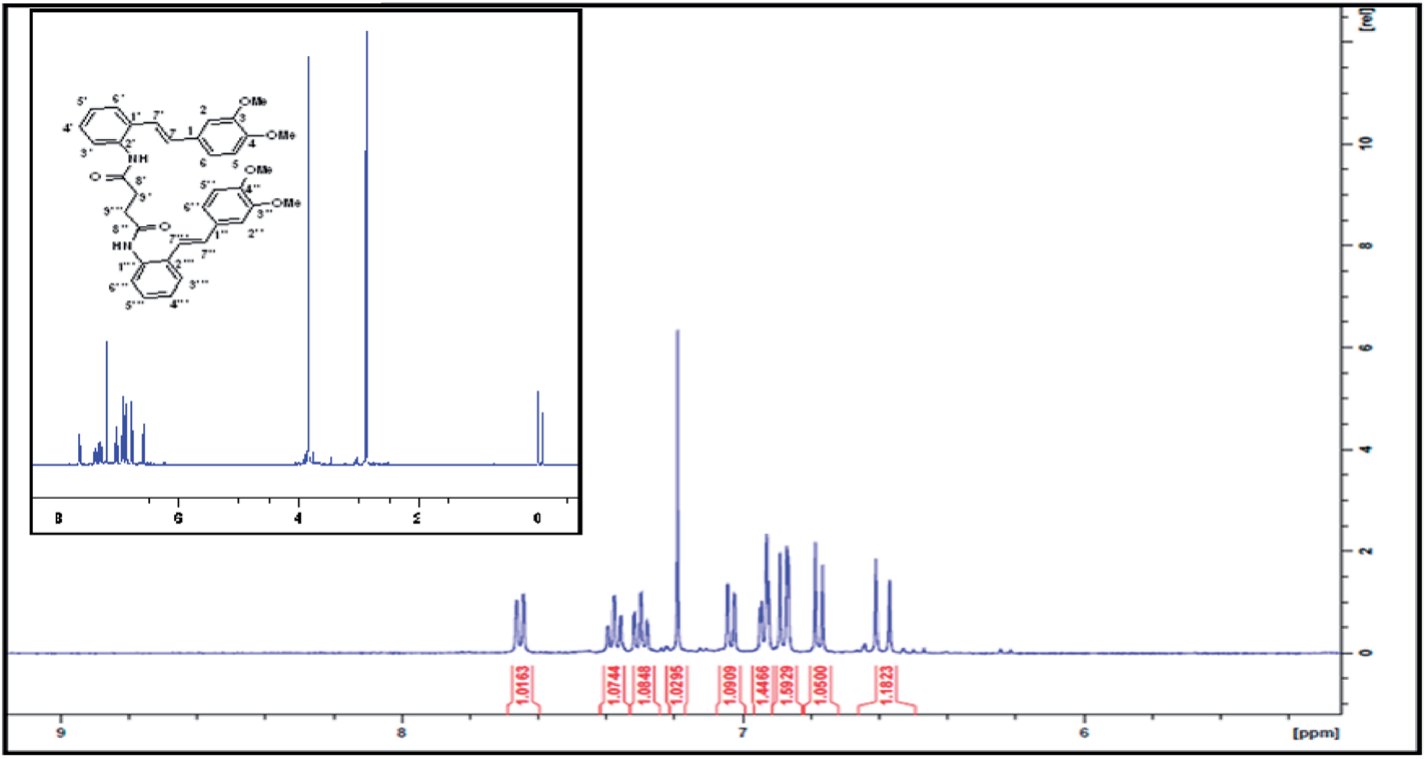
^1^H NMR (CDCl_3_, 400 MHz) spectrum of compound bis(2((*E*)-(3,4-dimethoxystyryl)phenyl)succinamide).

**Fig. 3:**
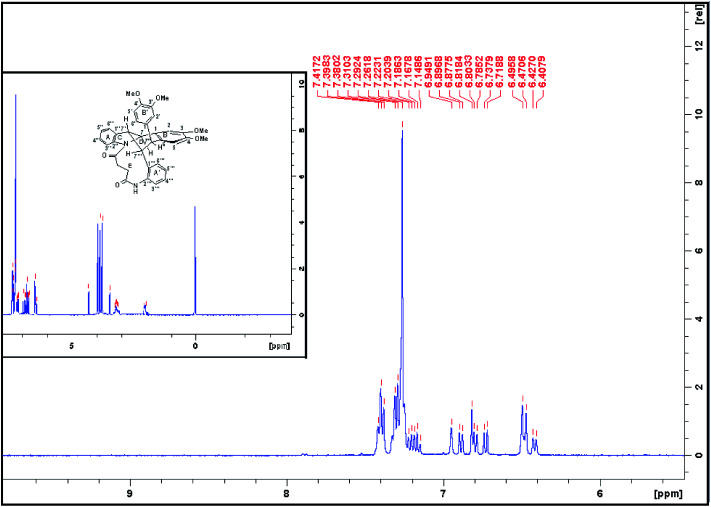
^1^H NMR (CDCl_3_, 400 MHz) spectrum of compound (73) Scheme 17.

We ruled out a structure to 57 (Scheme 13) as this is clearly a symmetrical structure which would yield two distinctive methoxy singlets which would not fit the NMR data for our macrocycle. The structure 61 (Scheme 14) was also ruled out as apart from the methylenes of the succinyl moiety, only two aliphatic CH signals would be expected (methoxy signals excluded). A physical model demonstrated that 65 (Scheme 15) would be two highly strained and this was subsequently ruled out. Notice that alternative structures 52 and 55 (Scheme 12) can also be excluded as either of these should give rise to only two methoxy signals in the NMR spectrum (Fig. 2 and 3).

## 4. Conclusion

Although the evidence presented is not, at this stage incontrovertible, we believe the proposed structure 73 is the best fit for the spectroscopic evidence and the evidence of the physical model. Therefore in the circumstances in which we find ourselves, we are compelled to present these significant results which suggest that we have here the first example of a macrocyclic bridged indoline generated by exposure of a hitherto unknown bis amino stilbene succinamide 72 to the single electron oxidant FeCl_3_. We hope, in more favourable circumstances, to present the results of a more comprehensive investigation in the future.

## 5. Experimental

### General remarks

Unless otherwise noted, materials were purchased from commercial suppliers and used without purification. Column chromatography was performed using Merck silica gel (0.040–0.063). IR spectra were recorded on a perkinElmer spectrum 400 FTIR/FT-FIRspectrophotometer were used; for centrifugal chromatography, Merck silica gel 60 PF_254_ containing gypsum were used. Nuclear magnetic resonance (NMR) spectra were obtained on a JEOL FT-NMR BRUKER AVN 400 and JEOL FT-NMR LA400 spectra are reported in units of ppm on the scale, relative to chloroform and the coupling constants are given in Hz. Mass spectra were measured using Agilent 6530 Accurate Mass Q-TOFLC/MS system.

### Phase I: preparation of (*E*)-2-(3,4-dimethoxystyryl)aniline (71) (see Table 1)

In a dry, two necked flask, the desired 2-iodo aniline (1 g, 4.56 × 10^−3^ mol) was dissolved in dry CH_3_CN (8 ml) and stirred under nitrogen. The solution was heated at 80 °C and refluxed for a few minutes. Palladium(ii) acetate (0.0102 g, 4.56 × 10^−5^ mol) was added, followed by triethylamine (2.5 ml, 4.56 × 10^−3^ mol) and 2-furyl phosphine (0.042 g, 1.82 × 10^−4^ mol). 3-4 Dimethoxystyrene (0.93 g, 5.9802 × 10^−3^) was then added to the reaction flask. The reaction mixture was heated under reflux for 3 h and stirring continued overnight at room. When the TLC indicated the complete consumption of starting material, the mixture was extracted with ethyl acetate (3 × 30 ml) and washed distilled water (3 × 30 ml). The resulting organic extracts were combined and solvent was removed under reduced pressure to yield crude product. Purification by column chromatography (7 : 3 hexane : ethyl acetate) afforded the desired product (71% yield).

**Table 1:** ^1^H NMR [400 Hz] and ^13^C DEPT135, CH_3_/CH NMR [100 Hz] in CDCl_3_

	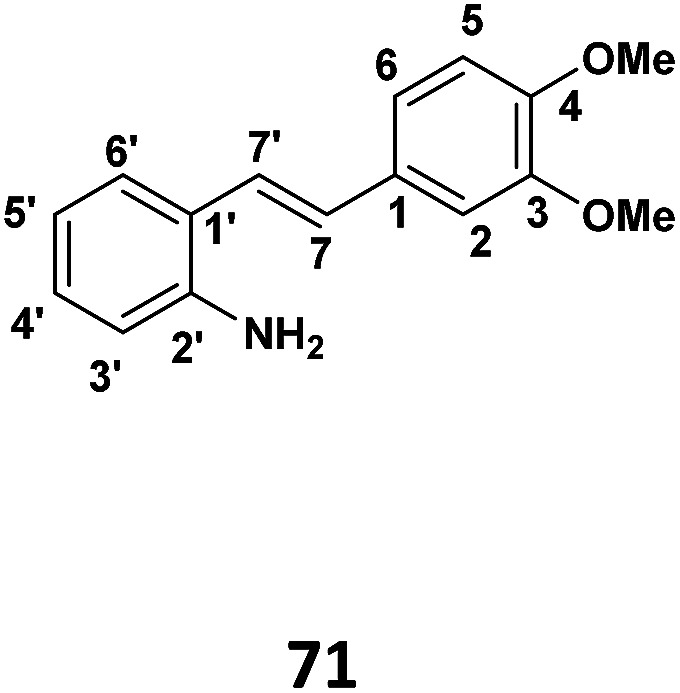		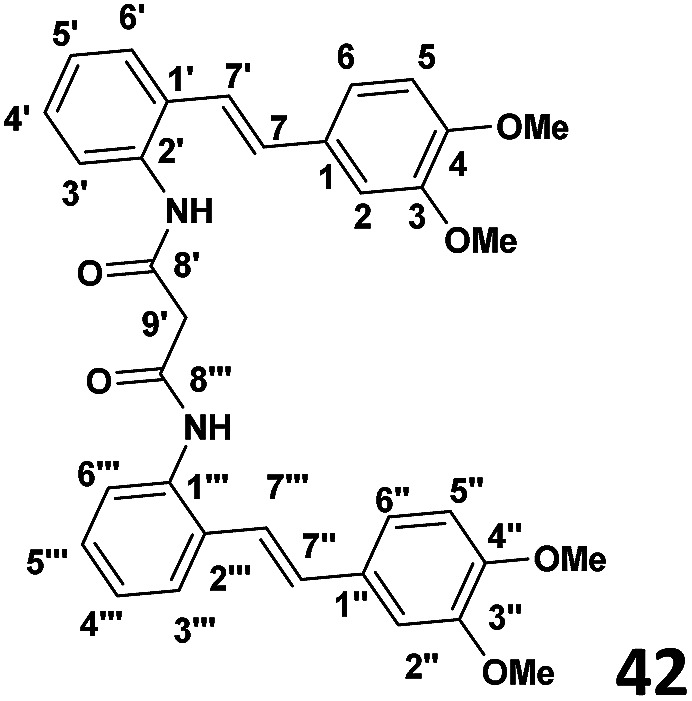		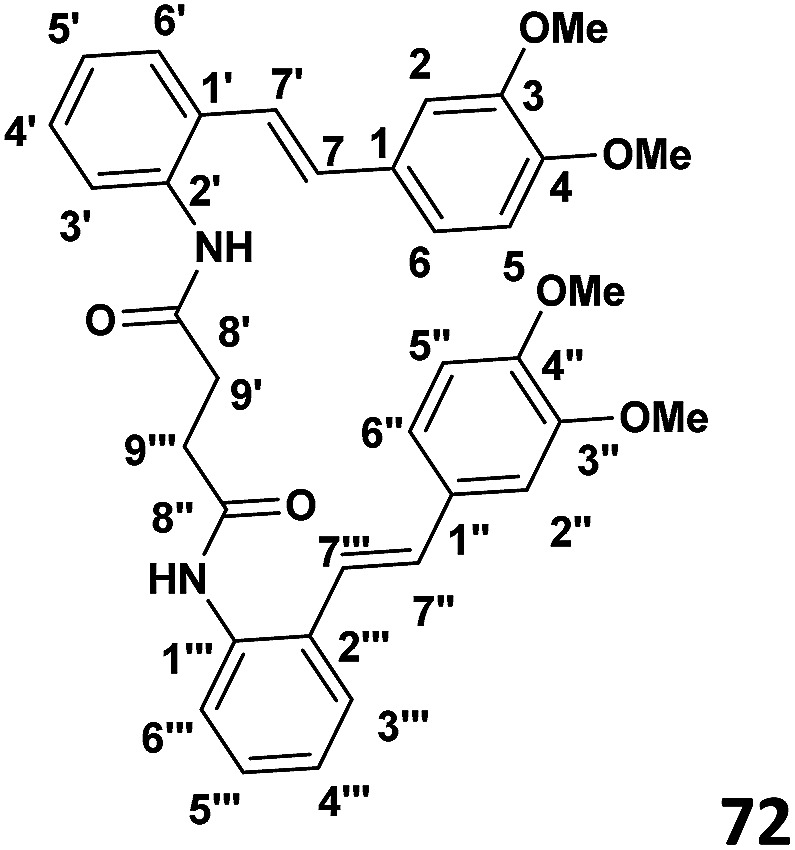		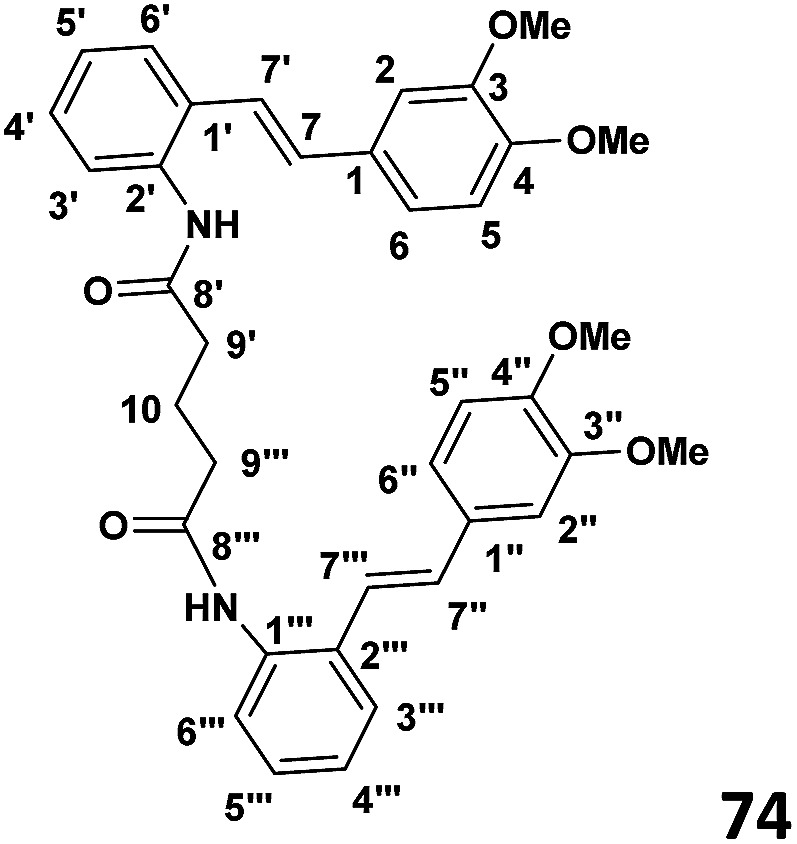		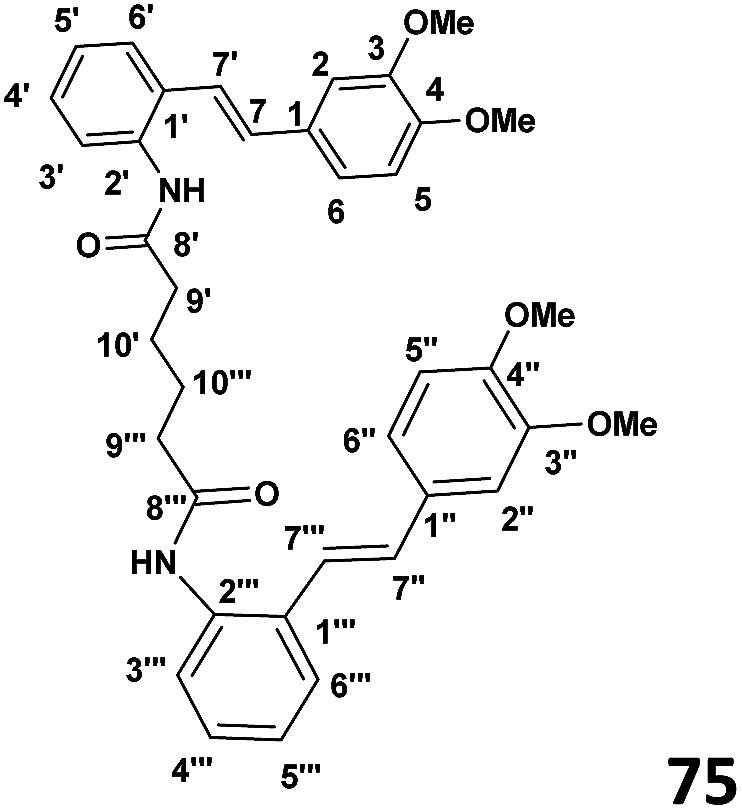	
	Chemical formula: C_16_H_17_NO_2_ molecular weight: 255.32		Chemical formula: C_35_H_34_N_2_O_6_ molecular weight: 578.67		Chemical formula: C_36_H_36_N_2_O_6_ molecular weight: 592.69		Chemical formula: C_37_H_38_N_2_O_6_ molecular weight: 606.72		Chemical formula: C_37_H_38_N_2_O_5_ molecular weight: 620.75	
	^1^H	^13^C	^1^H	^13^C	^1^H	^13^C	^1^H	^13^C	^1^H	^13^C
1	—	143.8	—	133.7	—	135.6	—	135.6	—	138.2
2	7.05 (s)	111	7.04 (S)	108.6	6.86 (S)	109.8	6.92 (S)	109.6	7.06 (S)	121.7
3	—	149.2	—	140.2	—	149.1	—	149.2	—	149.2
4	—	149	—	141.9	—	149.2	—	149.2	—	150.1
5	6.86 (d, *J* = 8.2 Hz)	116.2	6.69 (d, *J* = 8.2 Hz)	110.9	6.76 (d, *J* = 8.2 Hz)	111.2	6.83 (d, *J* = 8.2 Hz)	111.3	6.87 (d, *J* = 8.2 Hz)	111.3
6	7.04 (d, *J* = 8.2 Hz)	117.7	6.93 (d, *J* = 8.2 Hz)	120.5	6.92 (d, *J* = 8.2 Hz)	119.8	6.96 (d, *J* = 8.2 Hz)	121.1	7.06 (d, *J* = 8.2 Hz)	119.9
7	6.94 (d, *J* = 16 Hz)	130.2	7.05 (d, *J* = 16 Hz)	120.3	6.89 (d, *J* = 16 Hz)	121.2	6.93 (d, *J* = 16 Hz)	130.2	6.91 (d, *J* = 16 Hz)	132.5
1′	—	130.2	—	130.0	—	128.8	—	128.8	—	130.3
2′	—	130.8	—	130.0	—	128.9	—	128.9	—	133.7
3′	6.72 (d, *J* = 7.8 Hz)	124.1	7.52 (d, *J* = 7.8 Hz)	124.2	7.02 (d, *J* = 7.8 Hz)	128.5	7.03 (d, *J* = 7.7 Hz)	129.2	7.48 (d, *J* = 7.8 Hz)	126.9
4′	7.09 (t, *J* = 7.8 Hz)	128.4	7.19 (t, *J* = 7.8 Hz)	126.1	7.31 (t, *J* = 7.8 Hz)	128.2	7.32 (t, *J* = 7.7 Hz)	129.1	7.17 (t, *J* = 7.8 Hz)	125.5
5′	6.81 (t, *J* = 7.56 Hz)	124.2	7.23 (t, *J* = 7.8 Hz)	127.7	7.28 (t, *J* = 7.7 Hz)	129.7	7.37 (t, *J* = 7.7 Hz)	128.2	7.28 (t, *J* = 7.8 Hz)	128.1
6′	7.38 (d, *J* = 7.8 Hz)	127.3	7.75 (d, *J* = 7.8 Hz)	130.0	7.64 (d, *J* = 7.7 Hz)	127.1	7.68 (d, *J* = 7.7 Hz)	126.7	7.82 (d, *J* = 7.8 Hz)	124
7′	7.03 (d, *J* = 16 Hz)	128.6	6.84 (d, *J* = 16 Hz)	131.1	6.56 (d, *J* = 16 Hz)	132.4	6.62 (d, *J* = 16 Hz)	123.6	7.0 (d, *J* = 16 Hz)	132.5
8′	—	—	—	165.6	—	172.3	—	172.3	—	172.3
8′′′	—	—	—	165.6	—	172.3	—	172.3	—	172.3
9′	—	—	3.62 (S)	44.8	2.87 (s)	28.6	2.84 (t)	33.1	2.37 (dt)	33.9
9′′′	—	—	—	—	—	—	—	—	—	—
10	—	—	—	—	—	—	2.1 (m)	27.5	—	—
10′	—	—	—	—	—	—	—	—	2.42 (t)	25.1
10′′′	—	—	—	—	—	—	—	—	—	—
3-Ome	3.93 (s)	55.98	3.65 (S)	55.69	3.82 (S)	55.98	3.87 (S)	55.94	3.91 (S)	55.98
4-Ome	3.90 (s)	55.93	3.84 (S)	55.87	3.83 (S)	55.98	3.90 (S)	55.98	3.94 (S)	55.99

#### (*E*)-2-(3,4-Dimethoxystyryl)aniline

^1^H NMR (CDCl_3_, 400 MHz) *δ* 3.90 (3H, s), 3.93 (3H, s), 6.72, (1H, d, *J* = 7.8 Hz), 6.81 (1H, t, *J* = 7.8 Hz), 6.86 (2H, d, *J* = 8.2 Hz), 6.94 (1H, d, *J* = 16.0 Hz), 7.03 (1H, d, *J* = 16.0 Hz), 7.09 (1H, t,*J* = 7.8 Hz), 7.04 (2H, d, *J* = 8.2 Hz), and 7.38 (1H, d, *J* = 7.6 Hz); ^13^C{^1^H} NMR (CDCl_3_, 100 MHz) *δ* 55.93, 55.98, 108.9, 111.04, 116.2, 117.73, 128.5, 127.29.5, 124.15, 127.2, 128.39, 130.21, 130.81, 143.81, 148.9.

### Phase II: preparation of bis (2-(*E*)-(3,4-dimethoxystyryl)phenyl)malonamide (42) (see Table 1)

To a stirred solution of the malonyl chloride (0.027 g, 1.95 × 10^−4^ mol) prepared as above, and DMAP (0.052 g, 4.3 × 10^−4^ mol) in CH_2_Cl_2_ (4 ml), was added slowly dropwise amino stilbene (0.1 g, 3.9 × 10^−4^ mol) at 0 °C. The stirring was continued overnight and the reaction allowed warm to room temperature when TLC indicated complete consumption was extracted with ethyl acetate (2 × 30 ml) and the washed with distilled water (2 × 30 ml). The resulting organic extracts were combined and evaporated under reduced pressure to yield crude product. Purification by column chromatography (7 : 3 hexane : ethyl acetate) afforded the desired product (65% yield).

#### ((*E*)-2-(3,4-Dimethoxystyryl)phenyl)malonamide

^1^H NMR (CDCl_3_, 400 MHz) *δ* ppm: 7.04 (s), 6.69 (d, *J* = 8.2 Hz), 6.93 (d, *J* = 8.2 Hz), 7.05 (d, *J* = 16 Hz), 7.52 (d, *J* = 7.8 Hz), 7.19 (t, *J* = 7.8 Hz), 7.23 (t, *J* = 7.8 Hz), 7.75 (d, *J* = 7.8 Hz), 6.84 (d, *J* = 16 Hz). ^13^C NMR (CDCl_3_, 100 MHz) *δ* ppm: 133.7, 108.6, 140.0, 141.9, 120.57, 120.34, 130.02, 130.04, 124.21, 126.16, 127.7, 130.04, 131.12, 165.62, 44.83, 55.69, 55.87.

### Phase III: preparation of bis(2((*E*)-(3,4-dimethoxystyryl)phenyl)succinamide) (72) (see Table 1)

To a stirred solution of the succinyl chloride (0.030 g, 1.95 × 10^−4^ mol) and DMAP (0.052 g, 4.3 × 10^−4^ mol) in CH_2_Cl_2_ (4 ml) was added amino stilbene (0.1 g, 3.9 × 10^−4^ mol) drop wise at 0 °C. The stirring was continued overnight and the reaction allowed to warm to room temperature. When the TLC indicated the complete consumption of starting material, the reaction mixture was extracted with ethyl acetate (2 × 30 ml) and the washed with distilled water (2 × 30 ml). The resulting organic extracts were combined and evaporated under reduced pressure to yield crude product. Purification by column chromatography (7 : 3 hexane : ethyl acetate) afforded the desired product (67% yield).

#### ((*E*)-2-(3,4-Dimethoxystyryl)phenyl)succinamide

^1^H NMR (CDCl_3_, 400 MHz) *δ* ppm 6.86 (s), 6.76 (d, *J* = 8.2 Hz), 6.92 (d, *J* = 8.2 Hz), 6.89 (d, *J* = 16 Hz), 7.02 (d, *J* = 7.8 Hz), 7.31 (t, *J* = 7.8 Hz), 7.28 (t, *J* = 7.8 Hz), 7.64 (d, *J* = 7.8 Hz), 6.56 (d, *J* = 16 Hz), 2.87 (d, *J* = 6.68 Hz), 3.82 (S), 3.83 (S). ^13^C NMR (CDCl_3_, 100 MHz) *δ* ppm: 135.6, 109.8, 149.0, 111.2, 119.8, 128.8, 128.9, 128.5, 128.2, 129.7, 127.0, 172.3, 28.68, 55.98.

### Phase 4: preparation of bis(2((*E*)-(3,4-dimethoxystyryl)phenyl)glutaramide) (74) (see Table 1)

To a stirred solution of the glutaryl chloride (0.030 g, 1.95 × 10^−4^ mol) and DMAP (0.052 g, 4.3 × 10^−4^ mol) in CH_2_Cl_2_ (4 ml) was added slowly dropwise amino stilbene (0.1 g, 3.9 × 10^−4^ mol) drop wise at 0 °C. The stirring was continued overnight and the reaction allowed to warm to room temperature. When the TLC indicated the complete consumption of starting material, the reaction mixture was extracted with ethyl acetate (2 × 30 ml) and then washed with distilled water (2 × 30 ml). The resulting organic extracts were combined and evaporated under reduced pressure to yield crude product. Purification by column chromatography (7 : 3 hexane : ethyl acetate) afforded the desired product (62% yield).

#### ((*E*)-2-(3,4-Dimethoxystyryl)phenyl)glutaramide

^1^H NMR (CDCl_3_, 400 MHz) *δ* 6.92 (S), 6.83 (d, *J* = 8.2 Hz), 6.96 (d, *J* = 8.2 Hz), 6.93 (d, *J* = 16 Hz), 7.03 (d, *J* = 7.7 Hz), 7.32 (t, *J* = 7.7 Hz), 7.37 (t, *J* = 7.7 Hz), 7.68 (d, *J* = 7.7 Hz), 6.62 (d, *J* = 16 Hz), 2.84 (t, *J* = 6.52 Hz), 3.87 (S), 3.90 (S), ^13^C{^1^H} NMR (CDCl_3_, 100 MHz) *δ* 135.6, 109.6, 149.0, 149.2, 111.3, 121.1, 130.2, 128.8, 128.9, 129.17, 129.01, 129.01, 128.19, 126.74, 123.6, 172.3, 33.14, 27.5, 55.94, 55.98.

### Preparation of bis(2((*E*)-(3,4-dimethoxystyryl)phenyl)adipamide) (75) (see Table 1)

To a stirred solution of adipoyl chloride (0.030 g, 1.95 × 10^−4^ mol) and DMAP (0.052 g, 4.3 × 10^−4^ mol) in CH_2_Cl_2_ (4 ml) was added slowly dropwise amino stilbene (0.1 g, 3.9 × 10^−4^ mol) at 0 °C. The stirring was continued overnight and the reaction allowed to warm to room temperature. When the TLC indicated the complete consumption of starting material, the reaction mixture was extracted with ethyl acetate (2 × 30 ml) and the washed with distilled water (2 × 30 ml). The resulting organic extracts were combined and evaporated under reduced pressure to yield crude product. Purification by column chromatography (7 : 3 hexane : ethyl acetate) afforded the desired product (63% yield).

#### (*E*)-2-(3,4-Dimethoxystyryl)adipamide amides

^1^H NMR (CDCl_3_, 400 MHz) 7.06 (S), 6.87 (d, *J* = 8.2 Hz), 7.06 (d, *J* = 8.2 Hz), 6.91 (d, *J* = 16 Hz), 7.48 (d, *J* = 7.8 Hz), 7.17 (t, *J* = 7.8 Hz), 7.28 (t, *J* = 7.8 Hz), 7.82 (d, *J* = 7.8 Hz), 7.0 (d, *J* = 16 Hz), 2.37 (d, *J* = 6.56 Hz), 2.42 (d, *J* = 84 Hz), 3.91 (S), 3.94 (S). ^13^C{^1^H} NMR (CDCl_3_, 100 MHz) *δ* 138.2, 121.69, 149.0, 150.0, 111.33, 119.89, 132.53, 130.3, 133.7, 126.91, 125.46, 123.98, 132.53, 172.3, 33.93, 25.17, 25.17, 55.98, 55.99.

### The FeCl_3_ promoted oxidative cascade reactions of the aminostilbene succinamide dimer (73)

Bis(2((*E*)-(3,4-dimethoxystyryl)phenyl)succinamide (0.080 g, 1.58 × 10^−4^ mol) was dissolved in CH_2_Cl_2_ (25 ml). FeCl_3_·6H_2_O (0.042 g, 1.58 × 10^−4^ mol) was added to the mixture under nitrogen. The mixture was stirred at room temperature and monitored by TLC. After the consumption of the starting malenal, saturated ammonium chloride was added to the reaction mixture followed by extracted with ethyl acetate (3 × 25 ml). The combined organic fractions were dried over anhydrous sodium sulphate and evaporated under reduced pressure. Purification of the crude product by column chromatography (7 : 3 hexane : ethyl acetate) gave rise to a major product in 61% yield.

#### Aminostilbene succinamide dimer (73)

^1^H NMR (CDCl_3_, 400 MHz) 6.94 (S), 6.40 (d, *J* = 7.80 Hz), 6.71 (d, *J* = 7.80 Hz), 7.38 (d, *J* = 7.64 Hz), 6.78 (d, *J* = 7.64 Hz), 7.15 (t, *J* = 7.64 Hz), 7.26 (t, *J* = 7.64 Hz), 6.87 (d, *J* = 7.64 Hz), 6.85 (S), 6.48 (S). ^13^C{^1^H} NMR (CDCl_3_, 100 MHz) *δ* 176.3, 132.6, 132.44, 130.28, 129.9, 129.5, 128.6, 128.3, 127.9, 126.8, 122.0, 122.8, 112.8, 110.7, 109.9, 55.0, 55.9, 55.8, 55.9, 51.8, 49.7, 37.1, 37.1, 33.9.

## Conflicts of interest

There are no conflicts to declare.

## Supplementary Material

RA-008-C7RA12534H-s001
