# Supplementary material for: The synthesis of some novel stilbene dimers incorporating diamide tethers: studies in single electron transfer oxidation (FeCl3)
Source: RSC Adv. 2018 Jan 11;8(5):2506–20. doi: 10.1039/c7ra12534h (PMC9077395; doi:10.1039/c7ra12534h)
Supplement: RA-008-C7RA12534H-s001 [file RA-008-C7RA12534H-s001.pdf]

Electronic Supplementary Material (ESI) for RSC Advances.
This journal is © The Royal Society of Chemistry 2017

he synthesis of some novel stilbene dimers incorporating diamide tethers: studies in single electron transfer oxidation ($FeCl_3$)

Maryam Sadat Alehashem* [a], Azhar Bin Ariffin* [a], Amjad Ayad Qatran Al-Khdhairawi [b] and Noel F Thomas[a]*

[a]Department of Chemistry, Faculty of Science , University of Malaya, 50603 Kuala Lumpur, Malaysia

[b]Atta-ur-Rahman Institute for Natural Product Discovery and Faculty of Pharmacy, Universiti Teknologi MARA Selangor Branch, 42300 Puncak Alam, Selangor, Malaysia

*Corresponding authors: Maryam Sadat Alehashem, Email: maryam.alehashem@yahoo.com

Azhar Bin Ariffin , Email: azhar70@um.edu.my

Noel Francis Thomas, Email: noelfthomas@um.edu.my & noelfthomas@yahoo.com

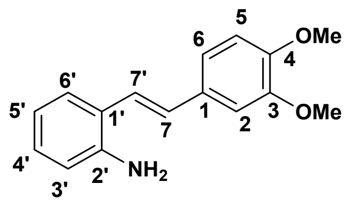

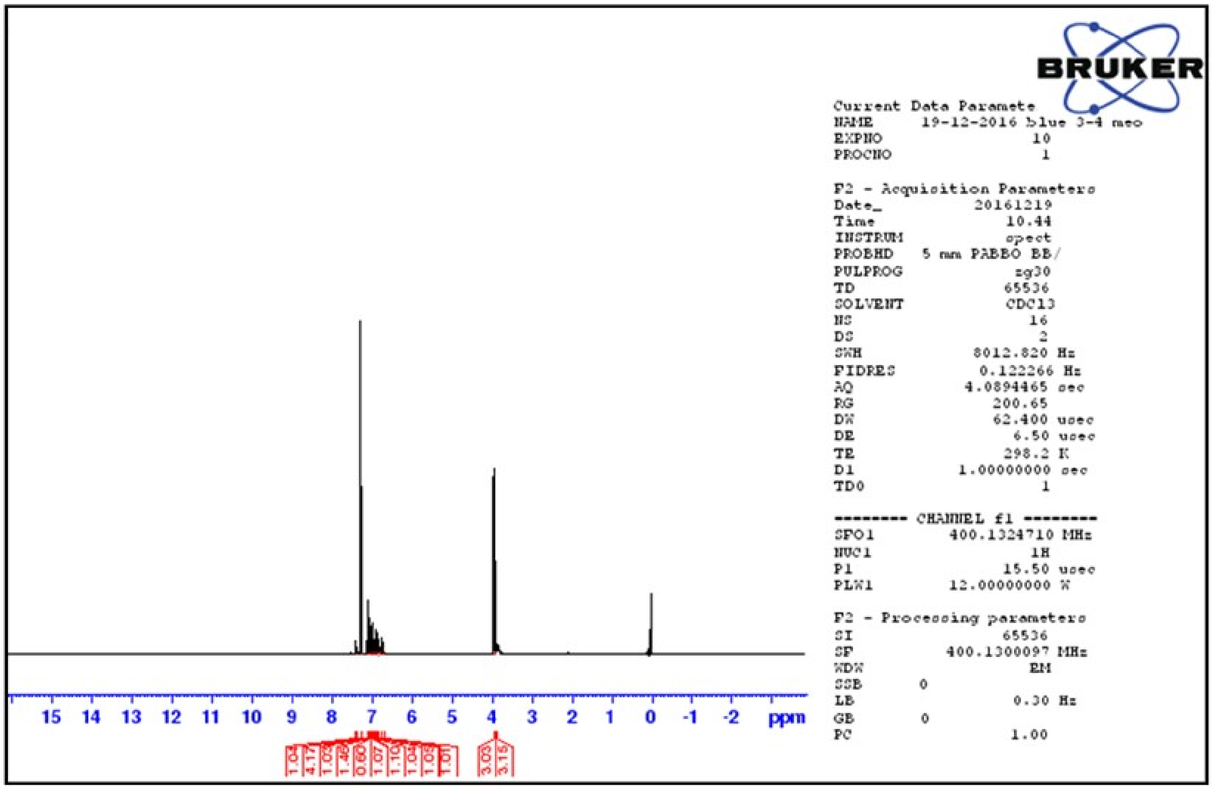

Figure S1: ^{1}H NMR ($CDCl_3$, 400MHz) spectrum of compound (E)-2-(3,4-dimethoxystyryl)aniline.

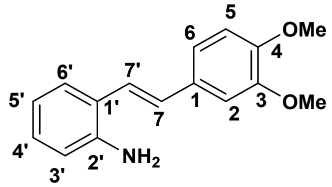

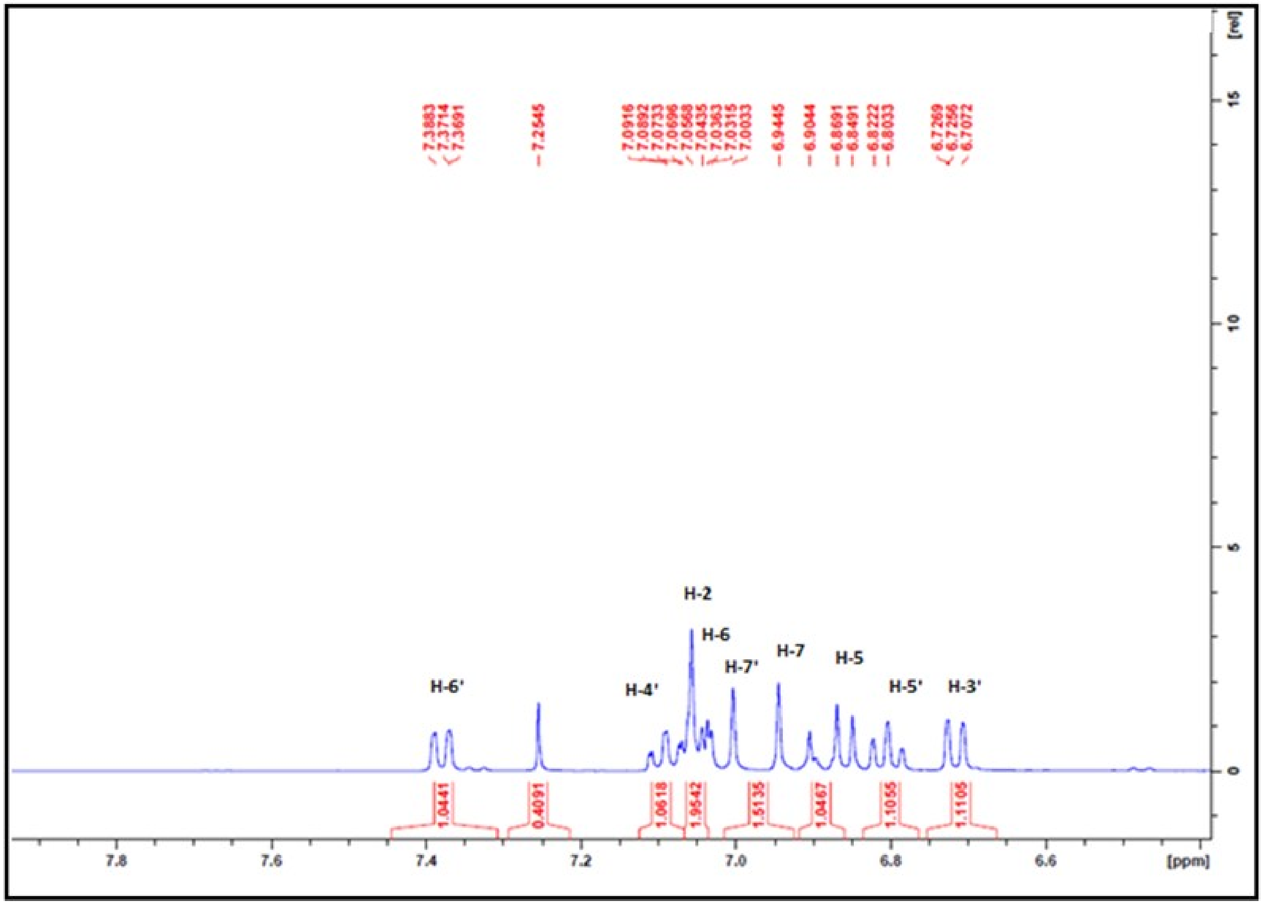

Figure S2: ^{1}H NMR ($CDCl_3$, 400MHz) spectrum of compound (E)-2-(3,4-dimethoxystyryl)aniline (EXPANSION).

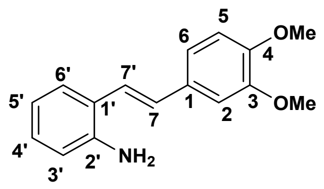

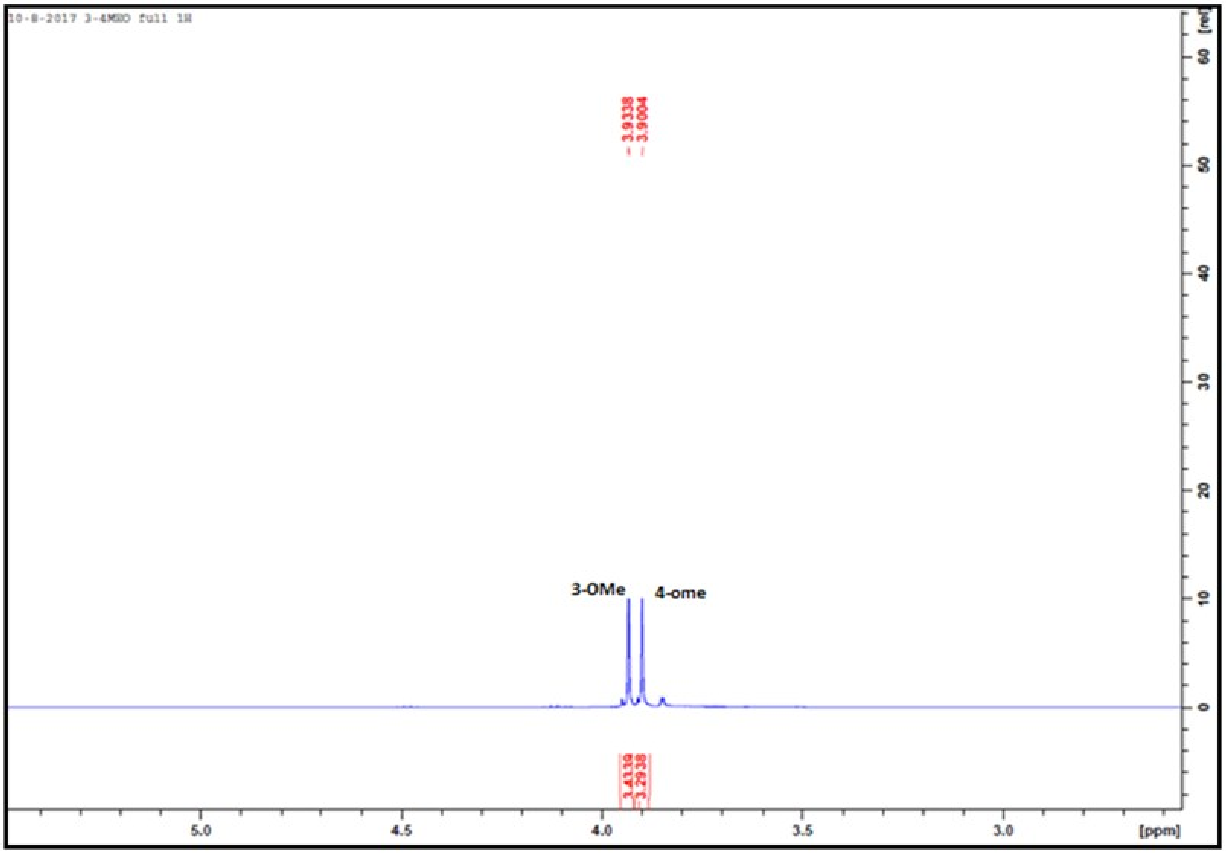

Figure S3: ^{1}H NMR ($CDCl_3$, 400MHz) spectrum of compound (E)-2-(3,4-dimethoxystyryl)aniline (EXPANSION).

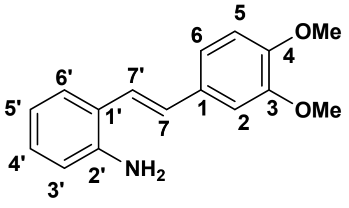

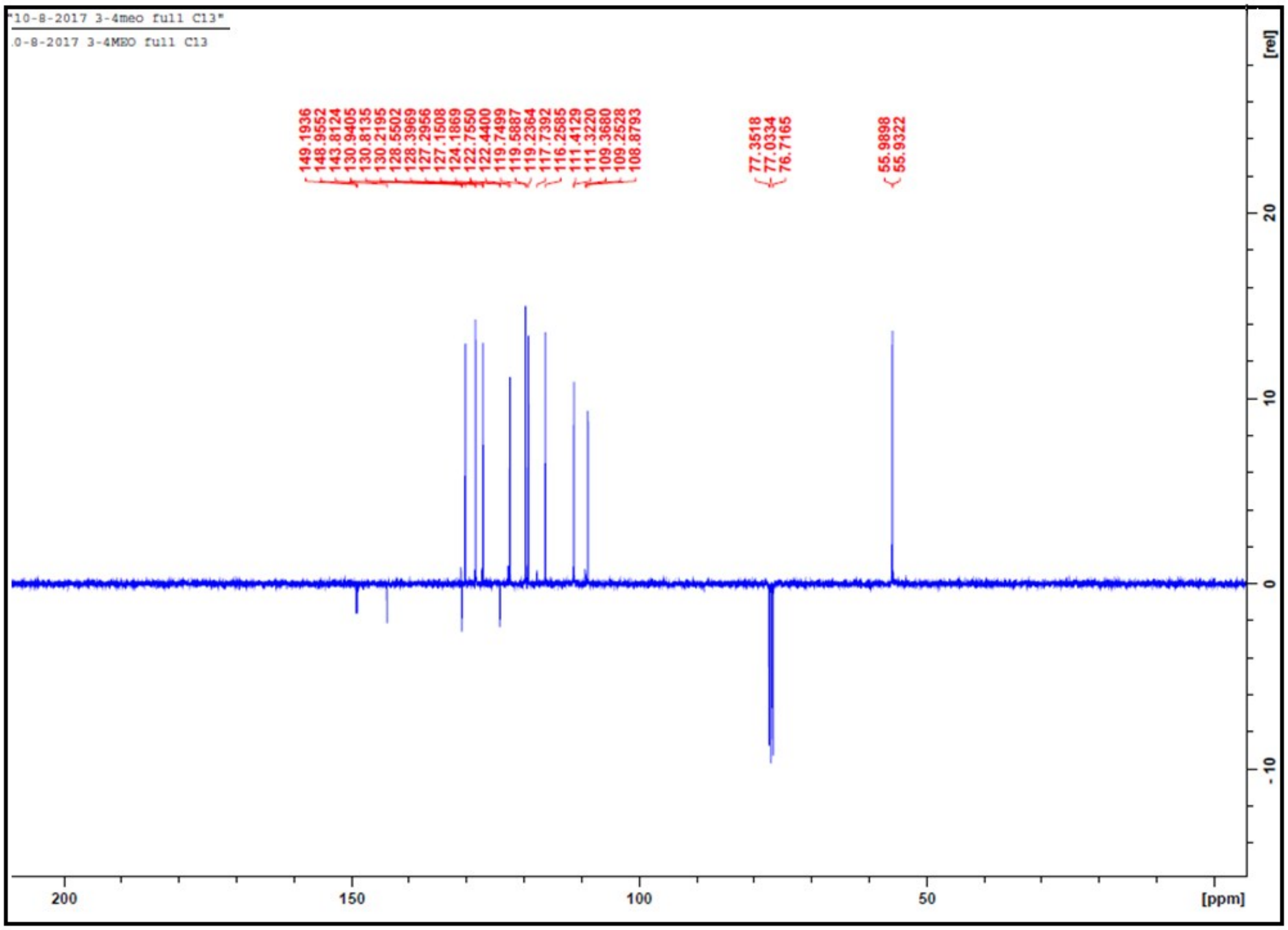

Figure S4: ^{13}C DEPT135,CH_3/CH po {^{1}H}($CDCl_3$, 400MHz) spectrum of compound (E)-2-(3,4-dimethoxystyryl)aniline.

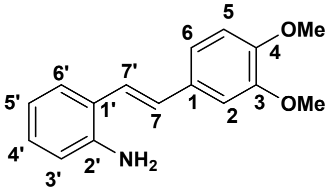

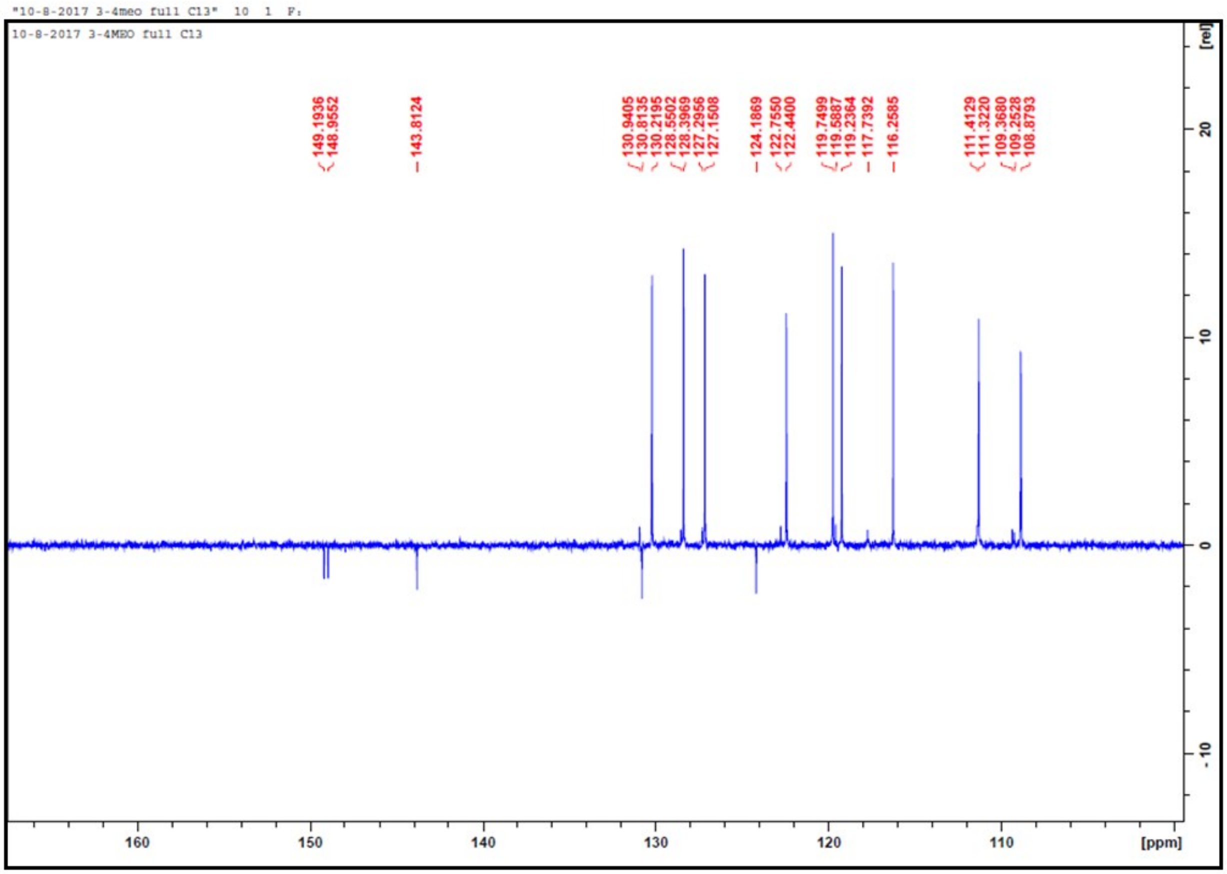

Figure S5: ^{13}C DEPT135,CH$_3$/CH po {^{1}H}(CDCl$_3$, 400MHz) spectrum of compound (E)-2-(3,4-dimethoxystyryl)aniline (EXPANSION).

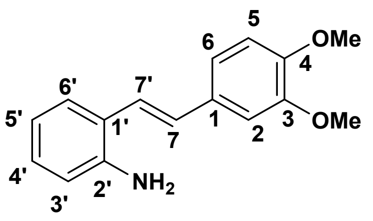

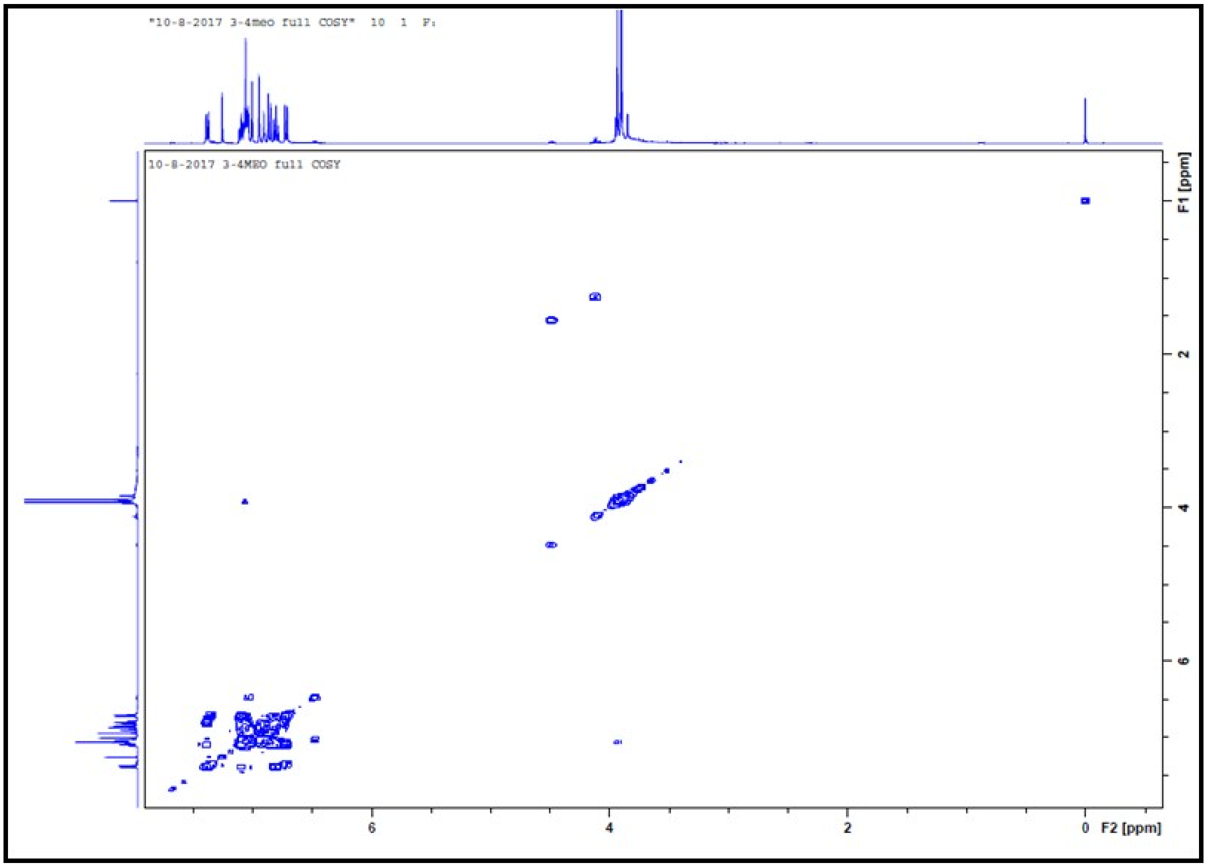

Figure S6: C COSY GPSW {^{1}H}($CDCl_3$, 400MHz) spectrum of compound (E)-2-(3,4-dimethoxystyryl)aniline.

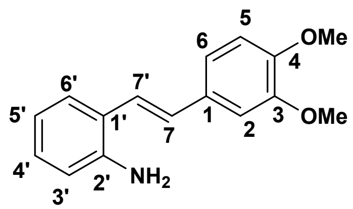

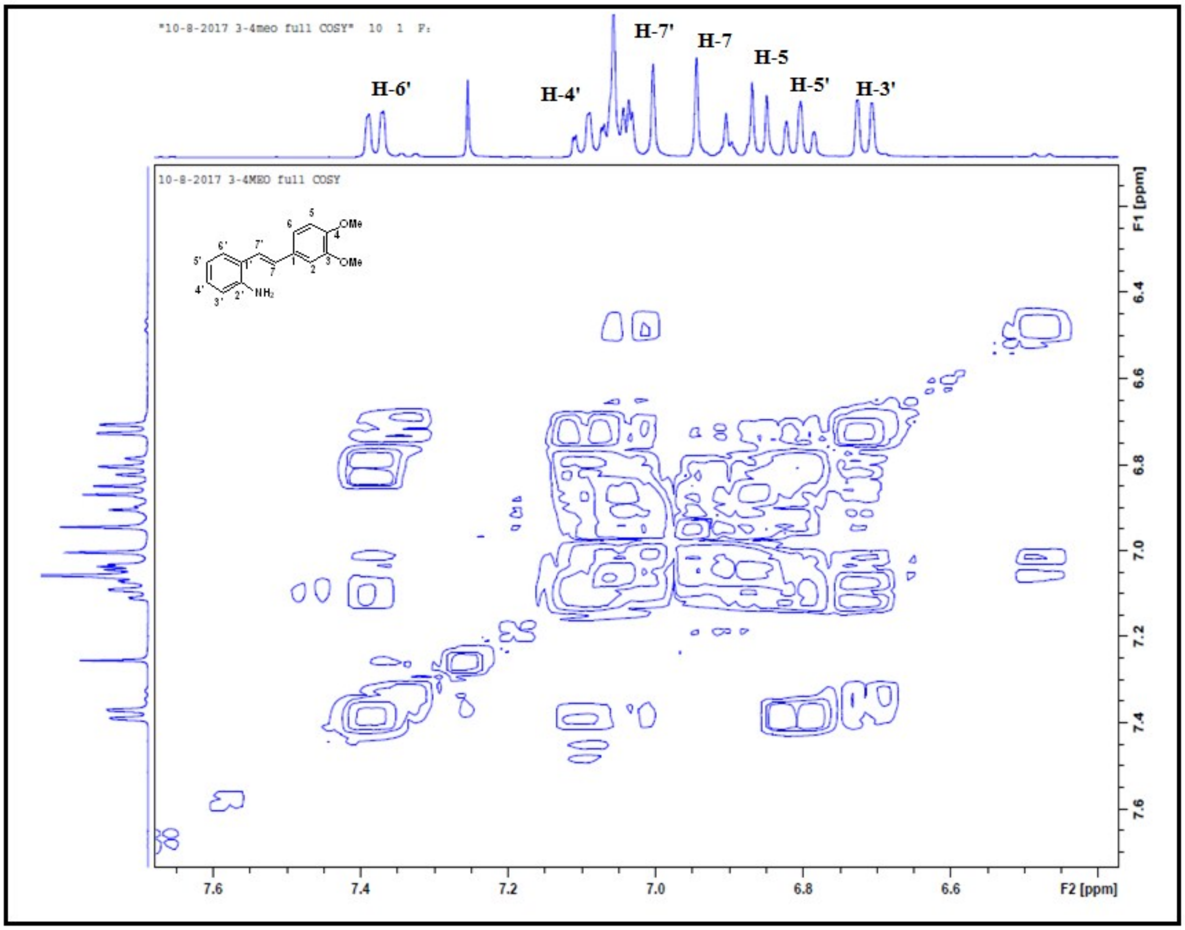

Figure S7: C COSY GPSW {^{1}H}($CDCl_3$, 400MHz) spectrum of compound (E)-2-(3,4-dimethoxystyryl)aniline (EXPANSION).

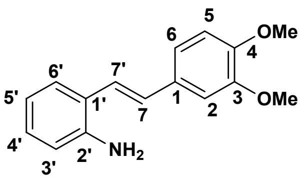

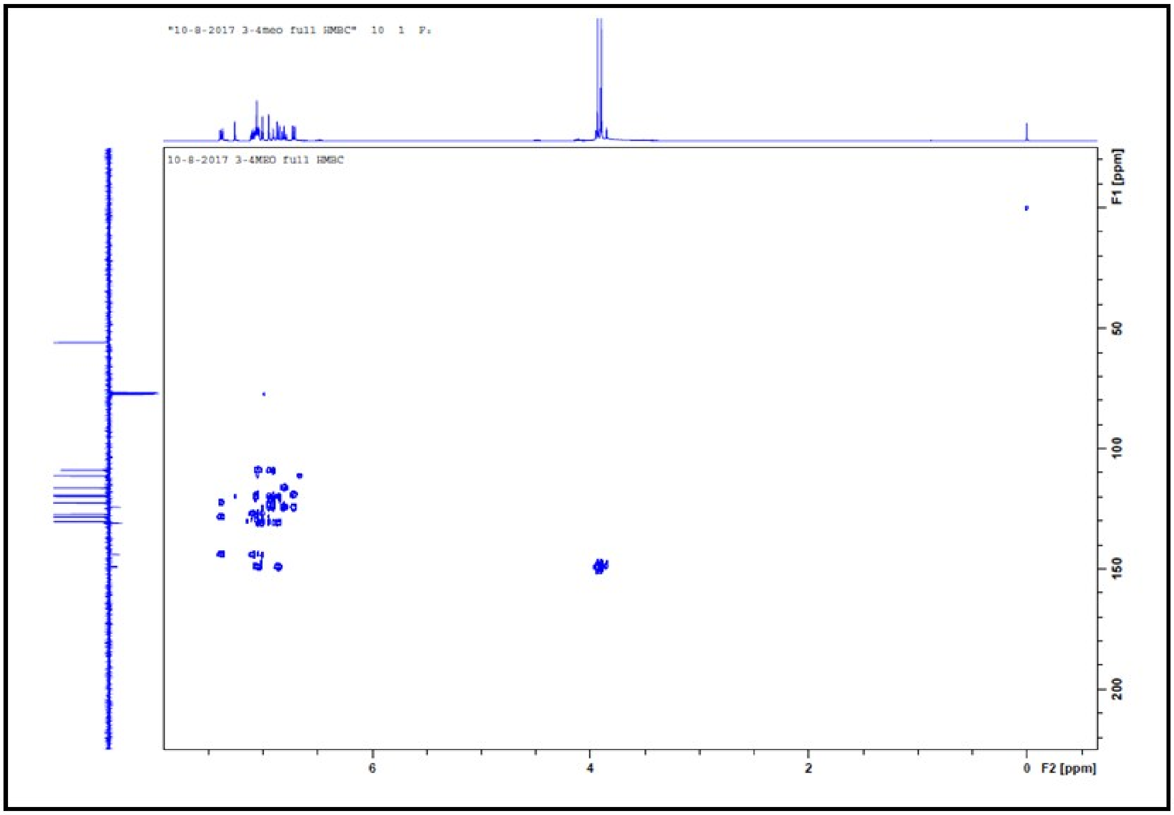

Figure S8: C HMBC GP {^{1}H- ^{13}C}(CDCl$_3$, 400MHz) spectrum of compound (E)-2-(3,4-dimethoxystyryl)aniline.

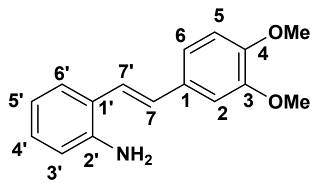

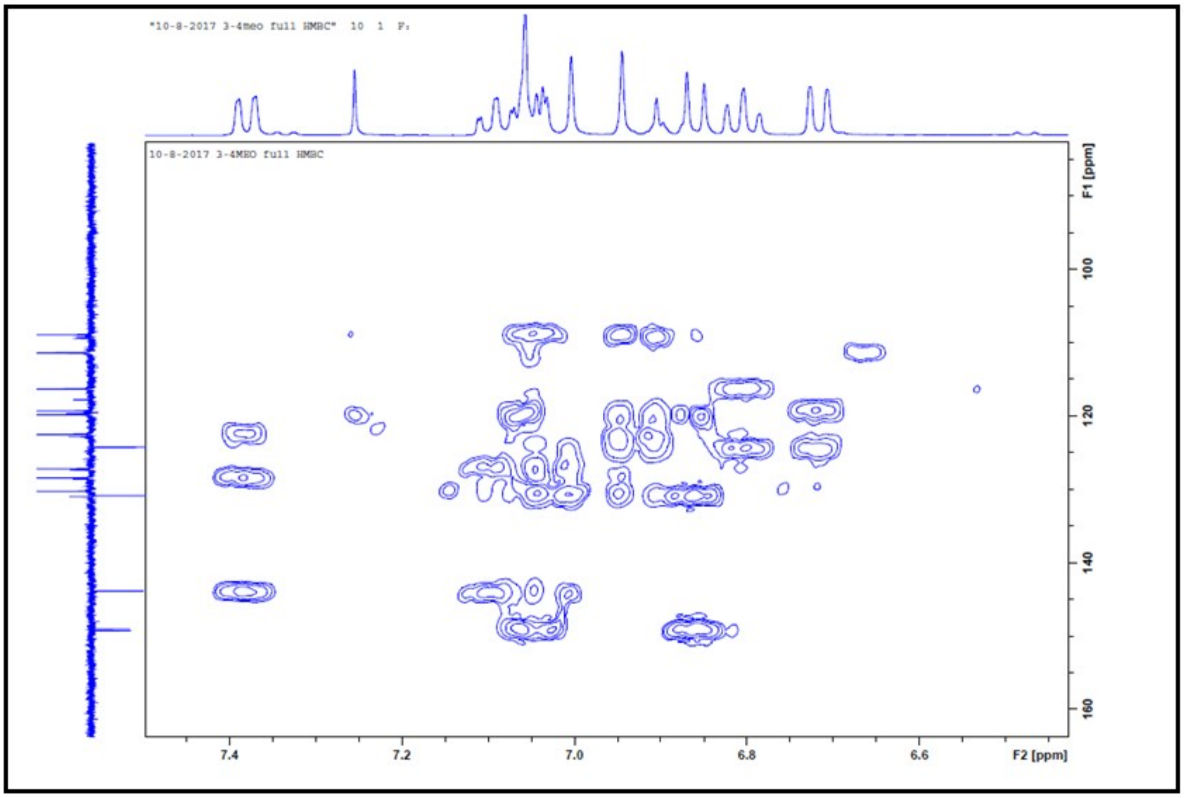

Figure S9: C HMBC GP {^{1}H- ^{13}C}($CDCl_3$, 400MHz) spectrum of compound (E)-2-(3,4-dimethoxystyryl)aniline (EXPANSION).

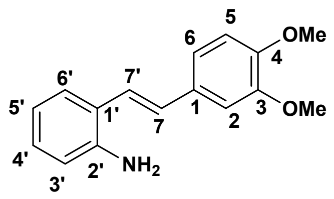

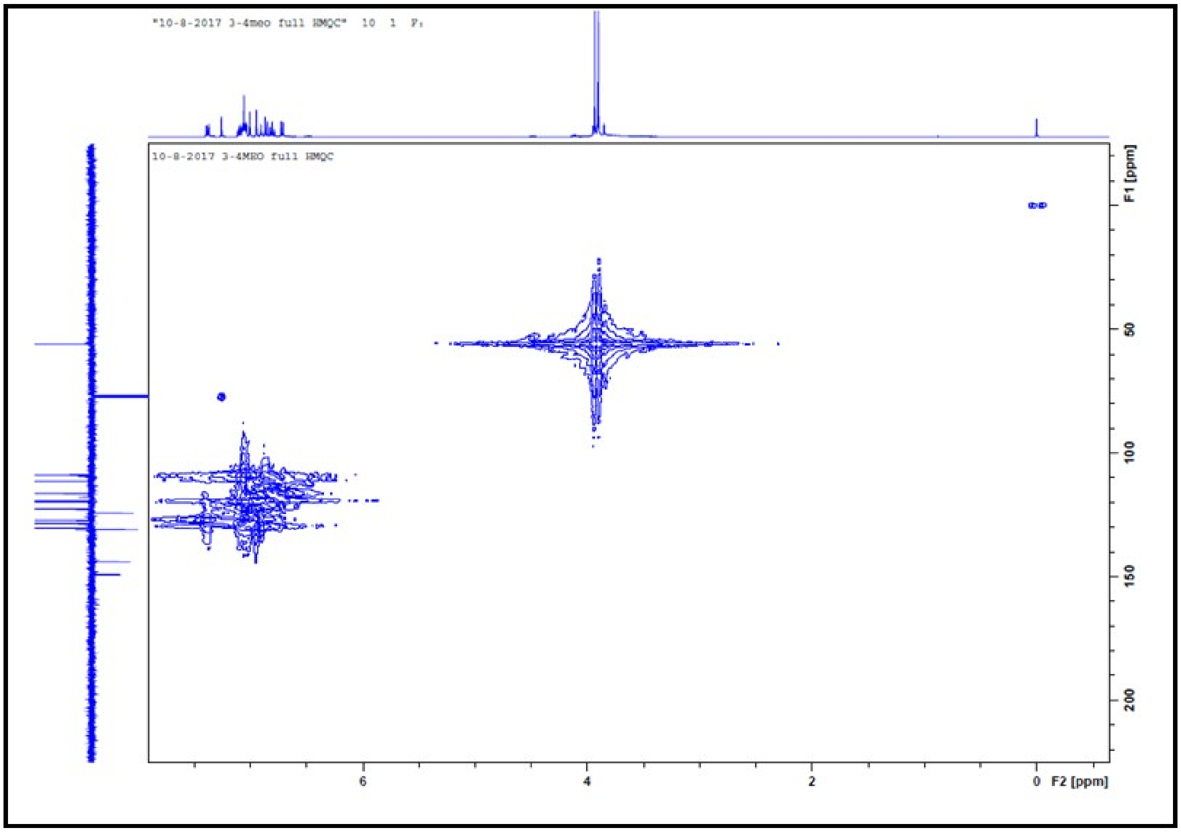

Figure S10: C HMQC GP {^{1}H-^{13}C}($CDCl_3$, 400MHz) spectrum of compound (E)-2-(3,4-dimethoxystyryl)aniline.

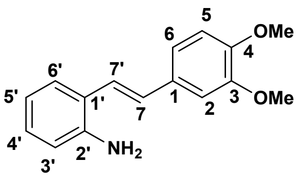

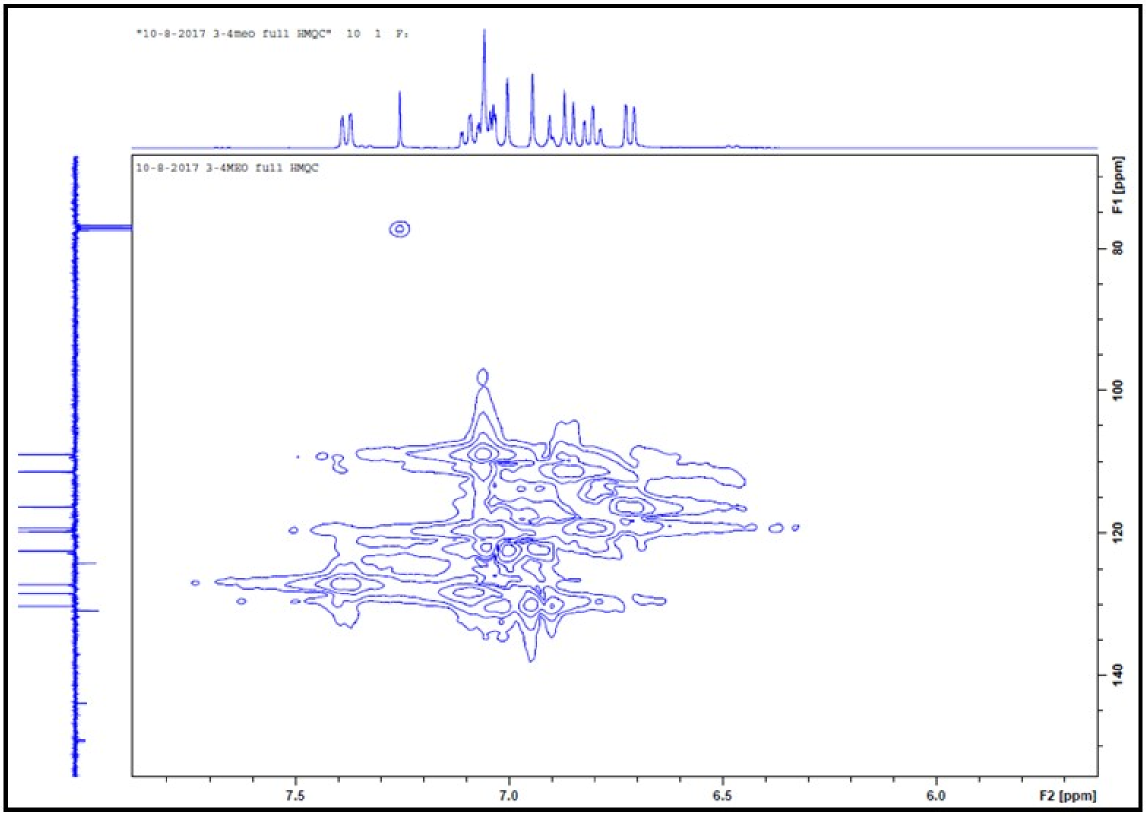

Figure S11: C HMQC GP {^{1}H-^{13}C}($CDCl_3$, 400MHz) spectrum of compound (E)-2-(3,4-dimethoxystyryl)aniline (EXPANSION).

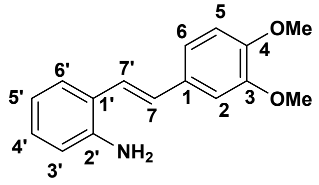

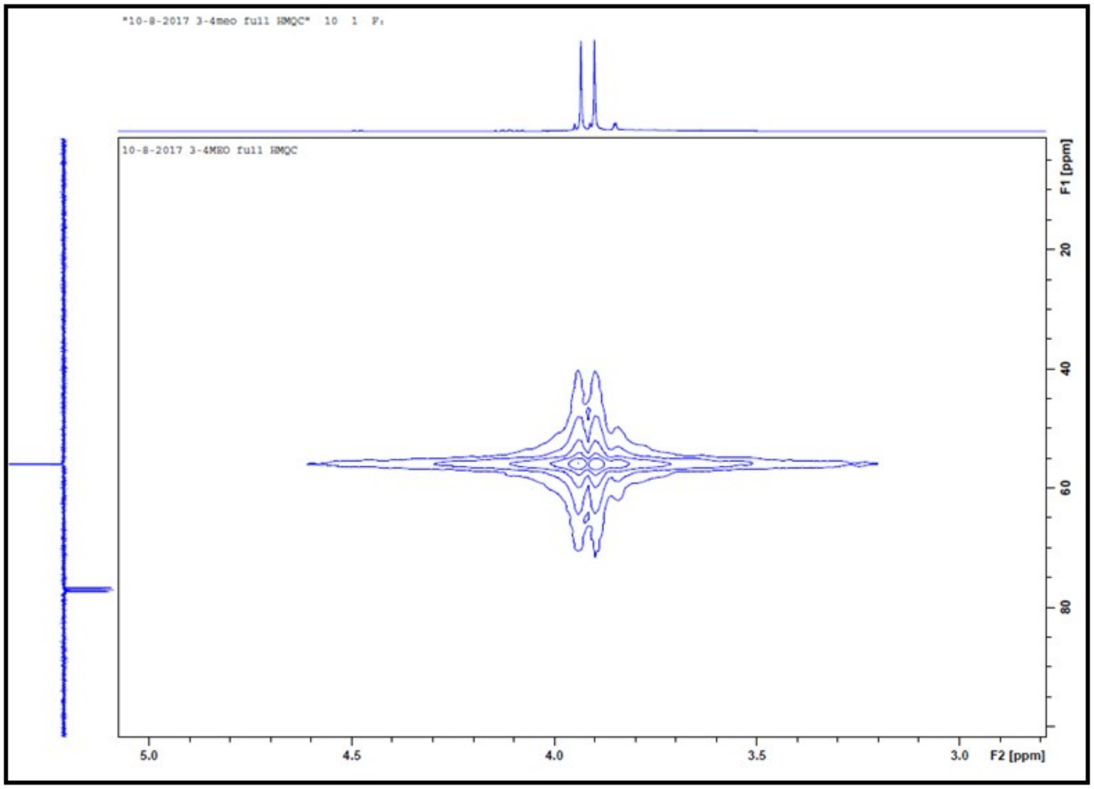

Figure S12: C HMQC GP {^{1}H-^{13}C}(CDCl$_3$, 400MHz) spectrum of compound (E)-2-(3,4-dimethoxystyryl)aniline (EXPANSION).

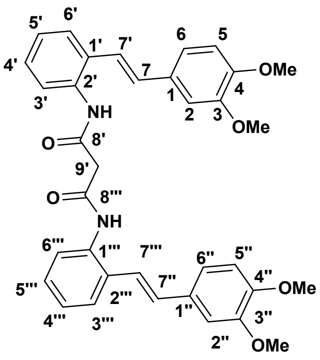

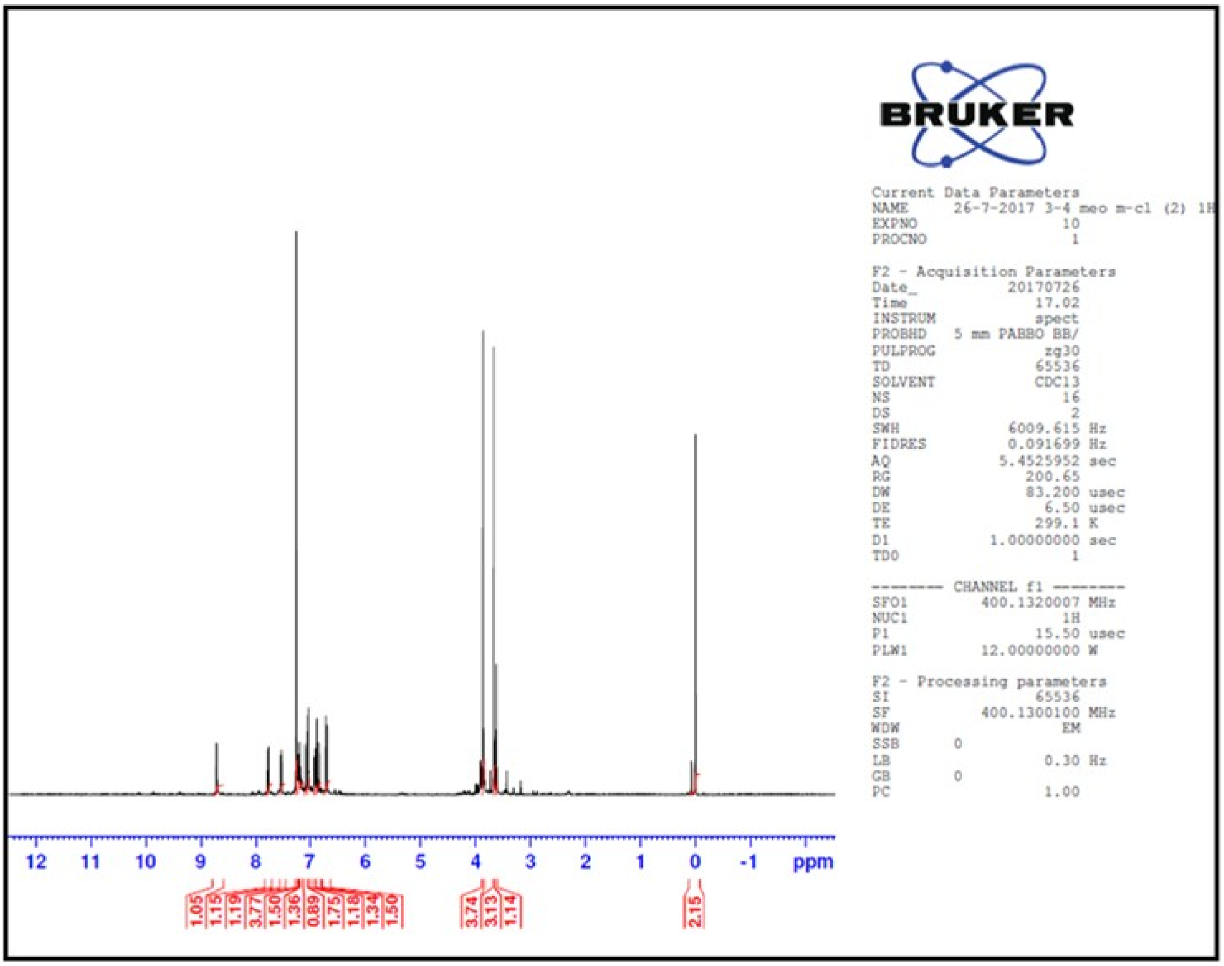

Figure S13: **^{1}H NMR ($CDCl_3$, 400MHz) spectrum of compound bis (2-(E))-3,4-dimethoxystyryl)phenyl) malonamide.**

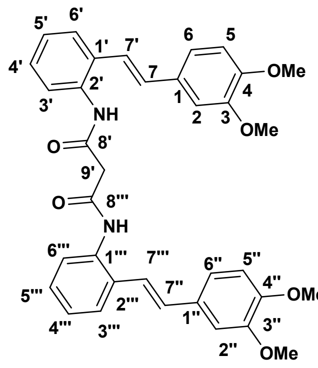

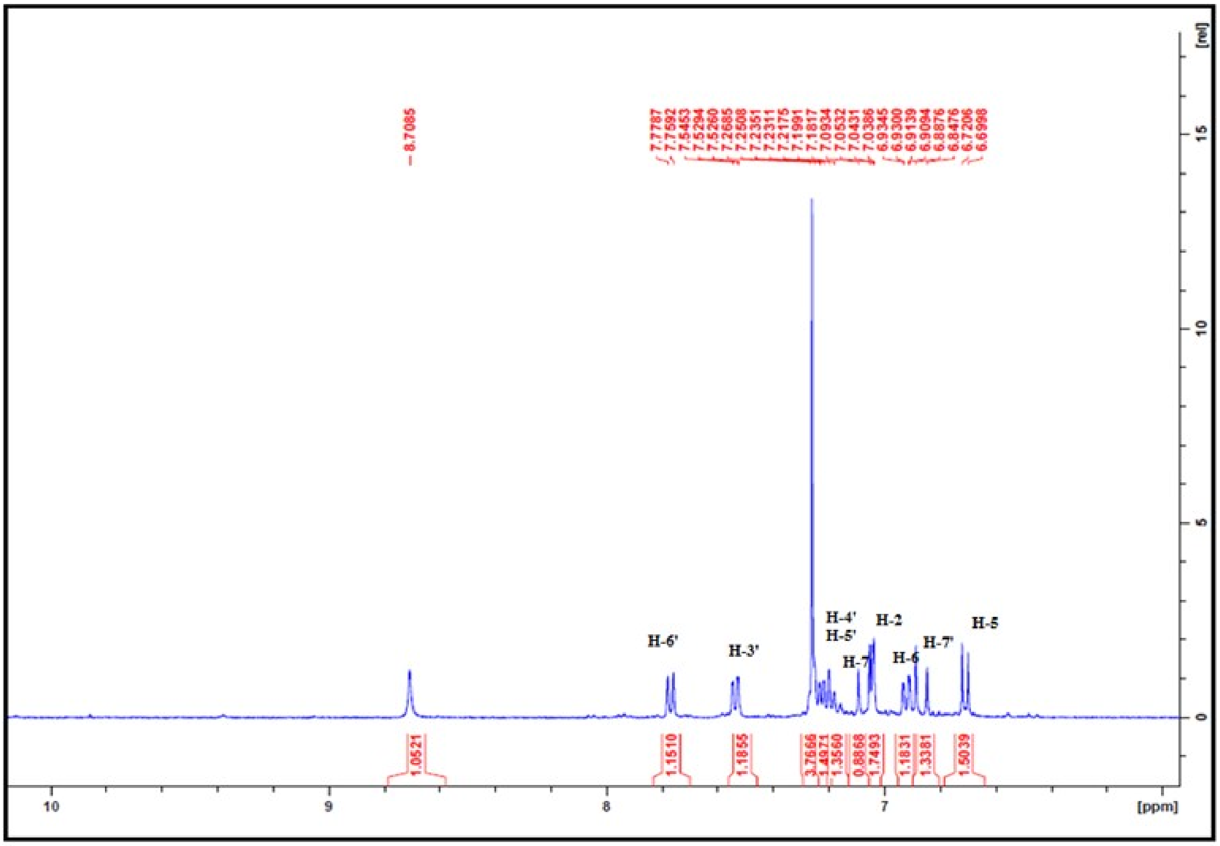

Figure S14: ¹H NMR ($CDCl_3$, 400MHz) spectrum of compound bis (2-(E))-3,4-dimethoxystyryl)phenyl) malonamide (EXPANSION).

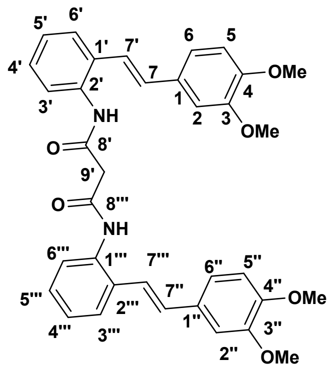

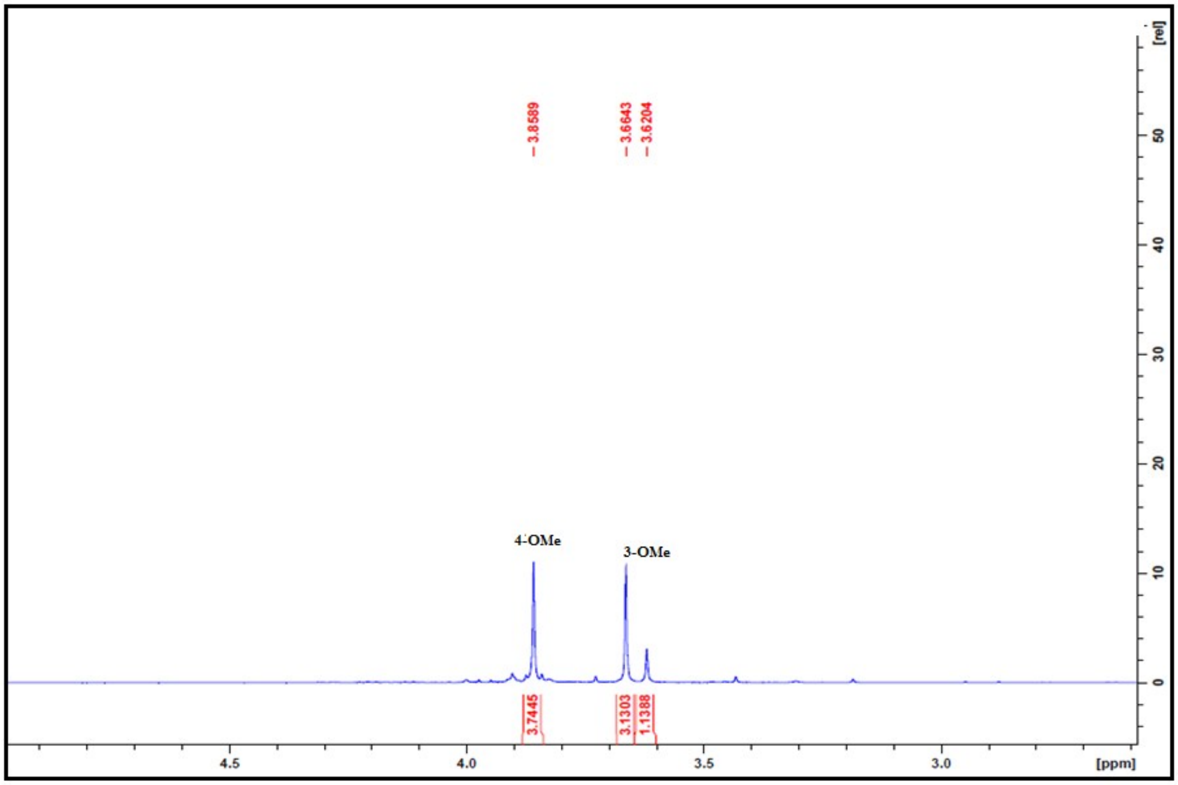

Figure S15: ^{1}H NMR (CDCl$_3$, 400MHz) spectrum of compound bis (2-(E))-3,4-dimethoxystyryl)phenyl) malonamide (EXPANSION).

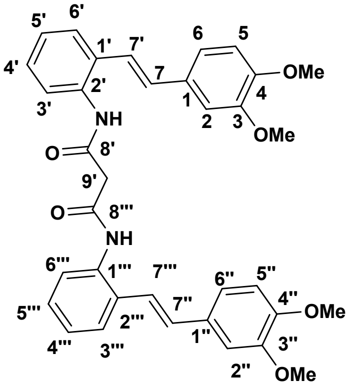

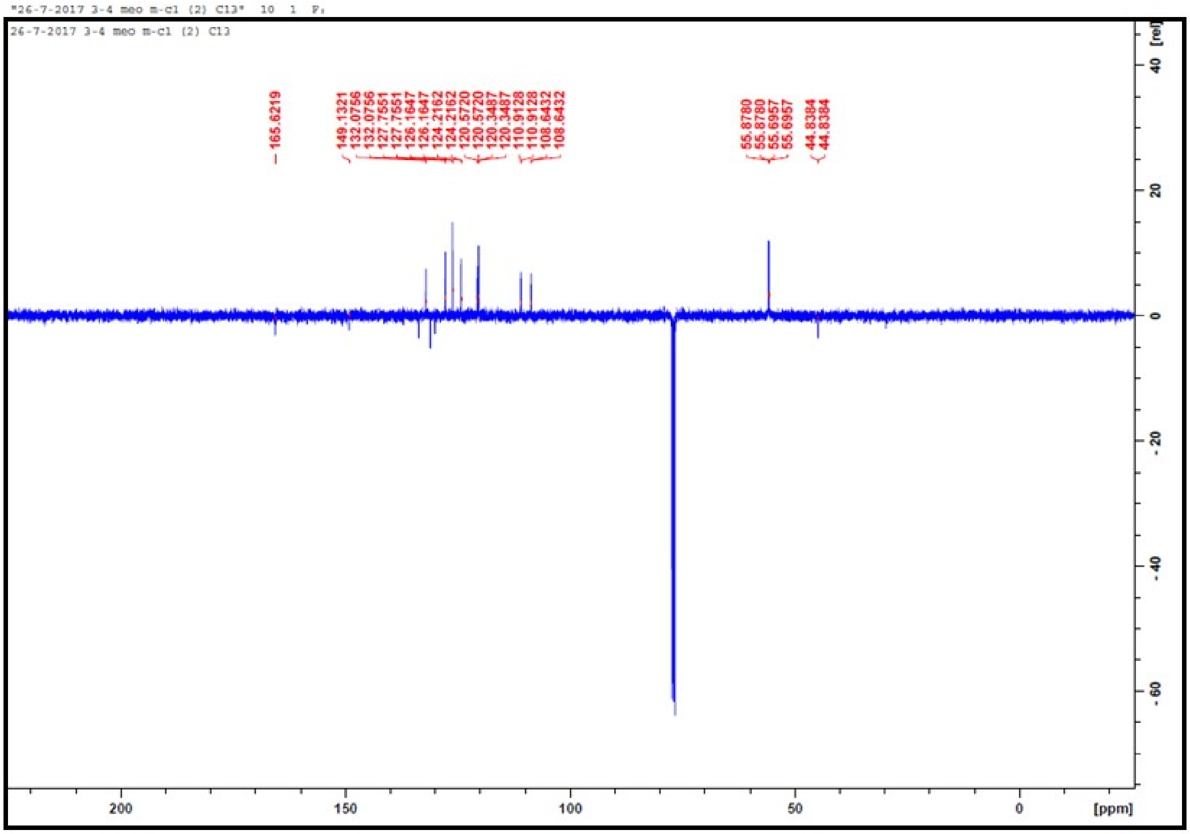

Figure S16: ^{13}C DEPT135,CH_3/CH po {^{1}H}($CDCl_3$, 400MHz) spectrum of compound bis (2-(E))-3,4-dimethoxystyryl)phenyl) malonamide.

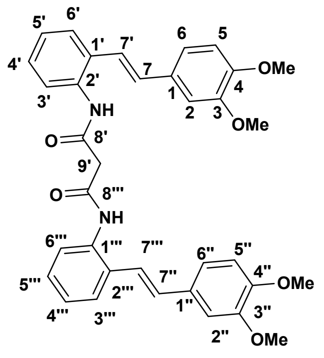

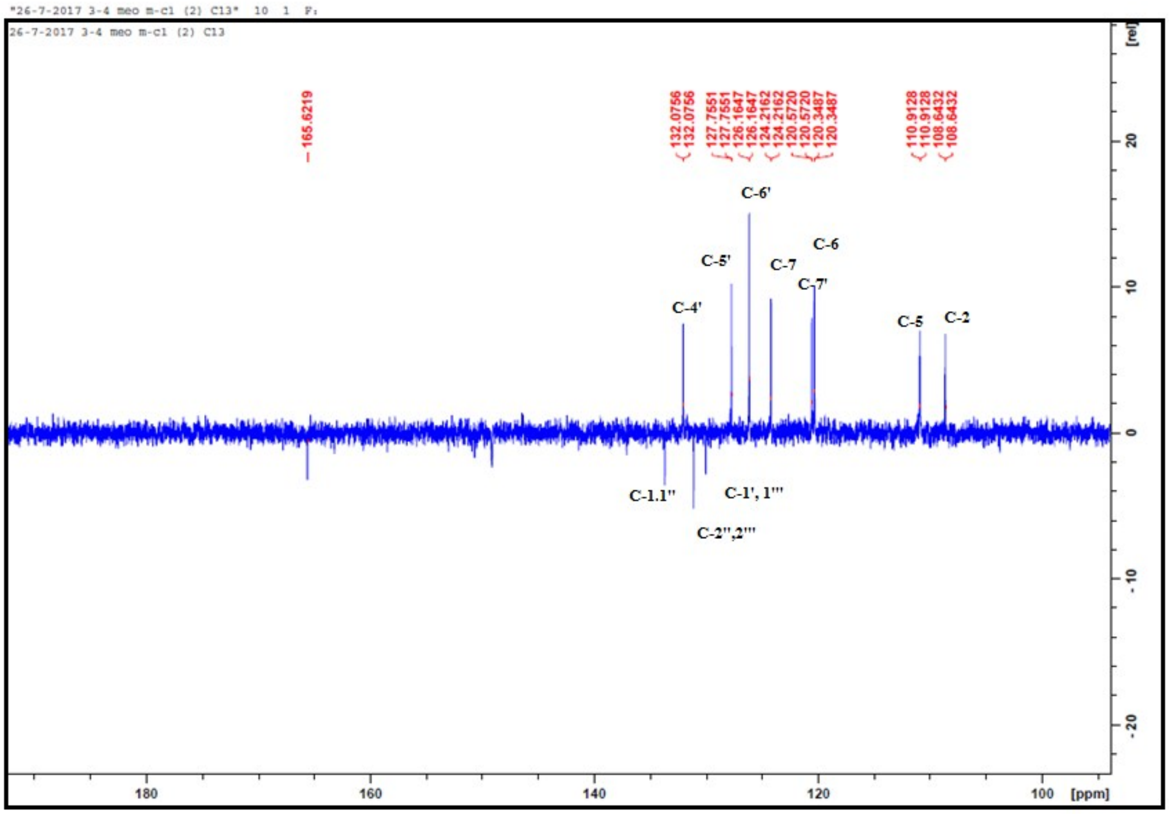

Figure S17: ^{13}C DEPT135,CH_3/CH po {^{1}H}($CDCl_3$, 400MHz) spectrum of compound bis (2-(E))-3,4-dimethoxystyryl)phenyl) malonamide (EXPANSION).

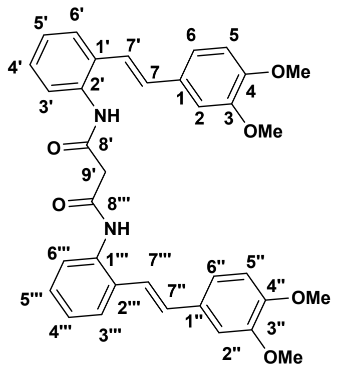

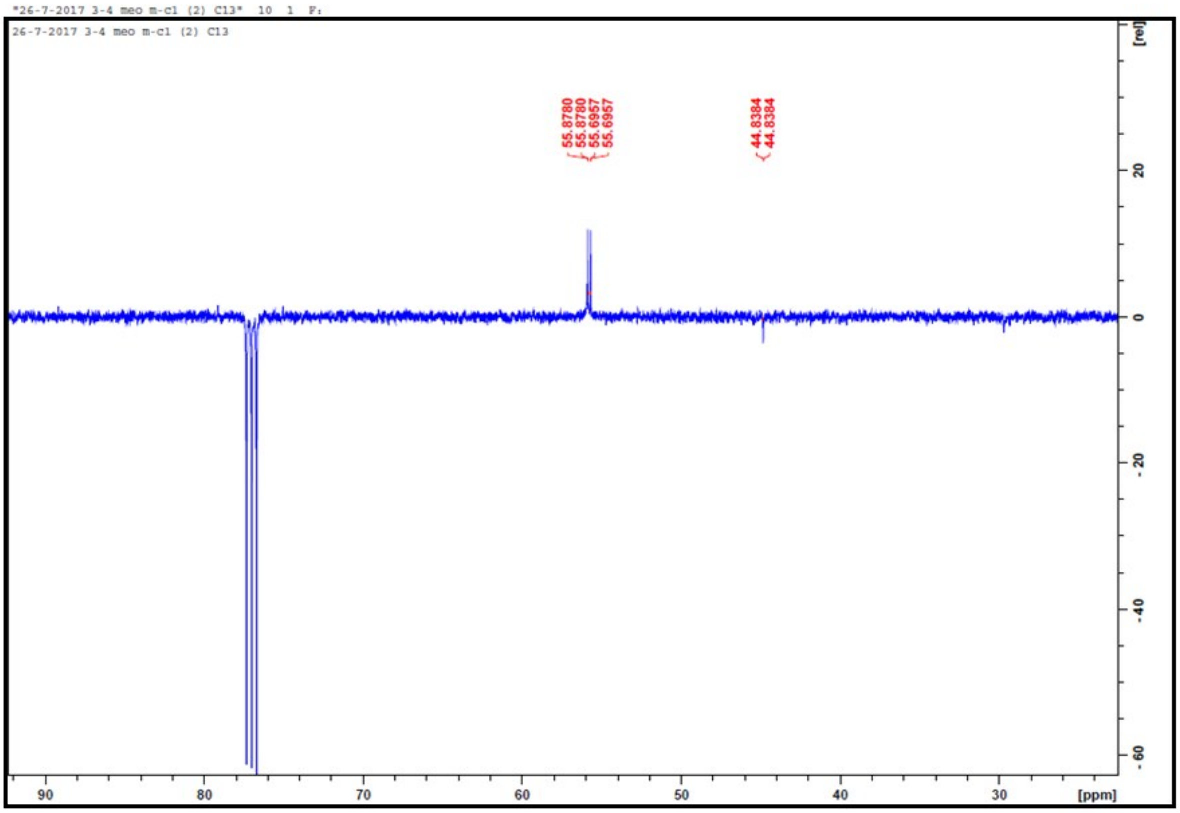

Figure S18: ^{13}C DEPT135,CH_3/CH po {^{1}H}($CDCl_3$, 400MHz) spectrum of compound bis (2-(E))-3,4-dimethoxystyryl)phenyl) malonamide (EXPANSION).

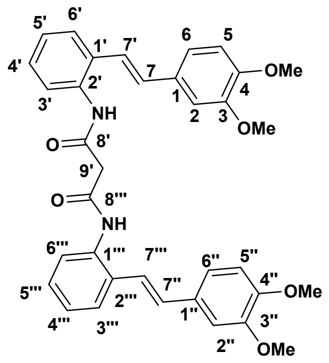

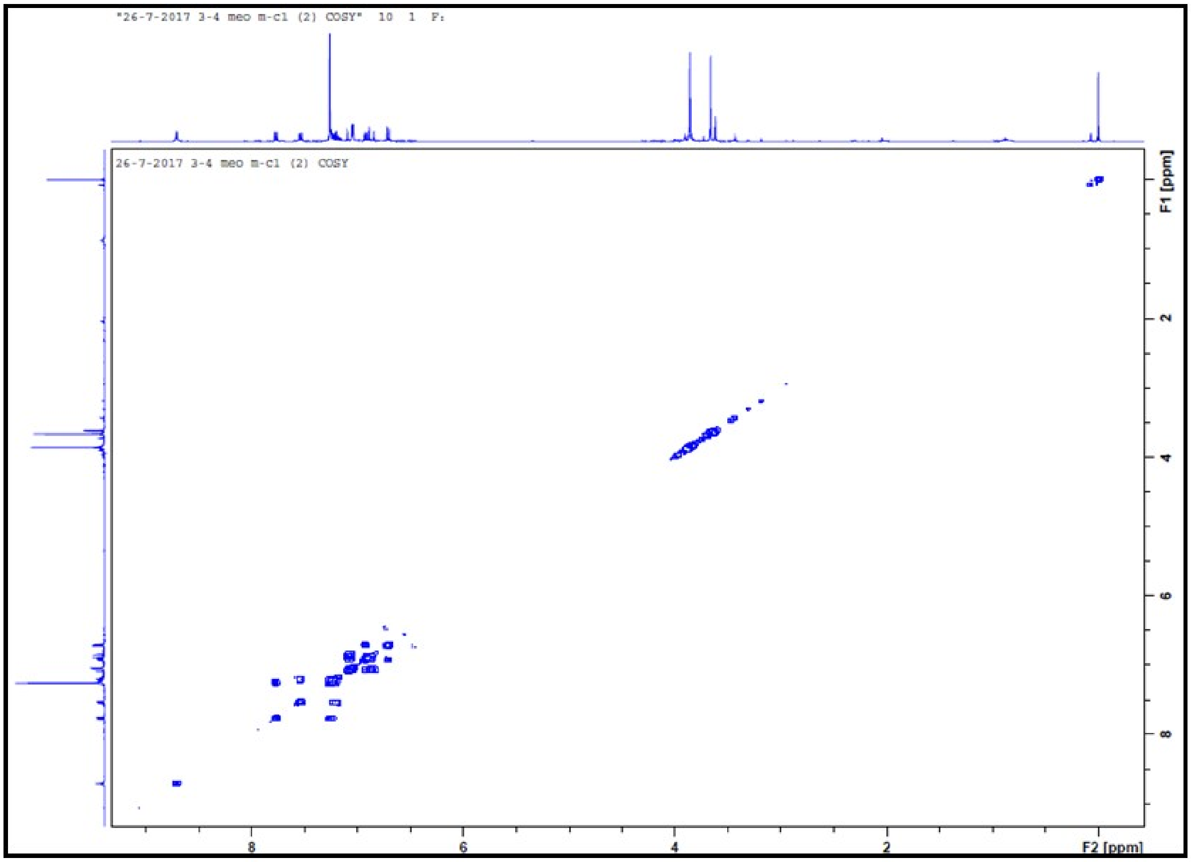

Figure S19: C COSY GPSW {^{1}H}($CDCl_3$, 400MHz) spectrum of compound bis (2-(E))-3,4-dimethoxystyryl)phenyl) malonamide.

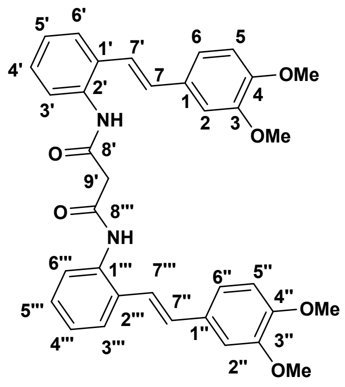

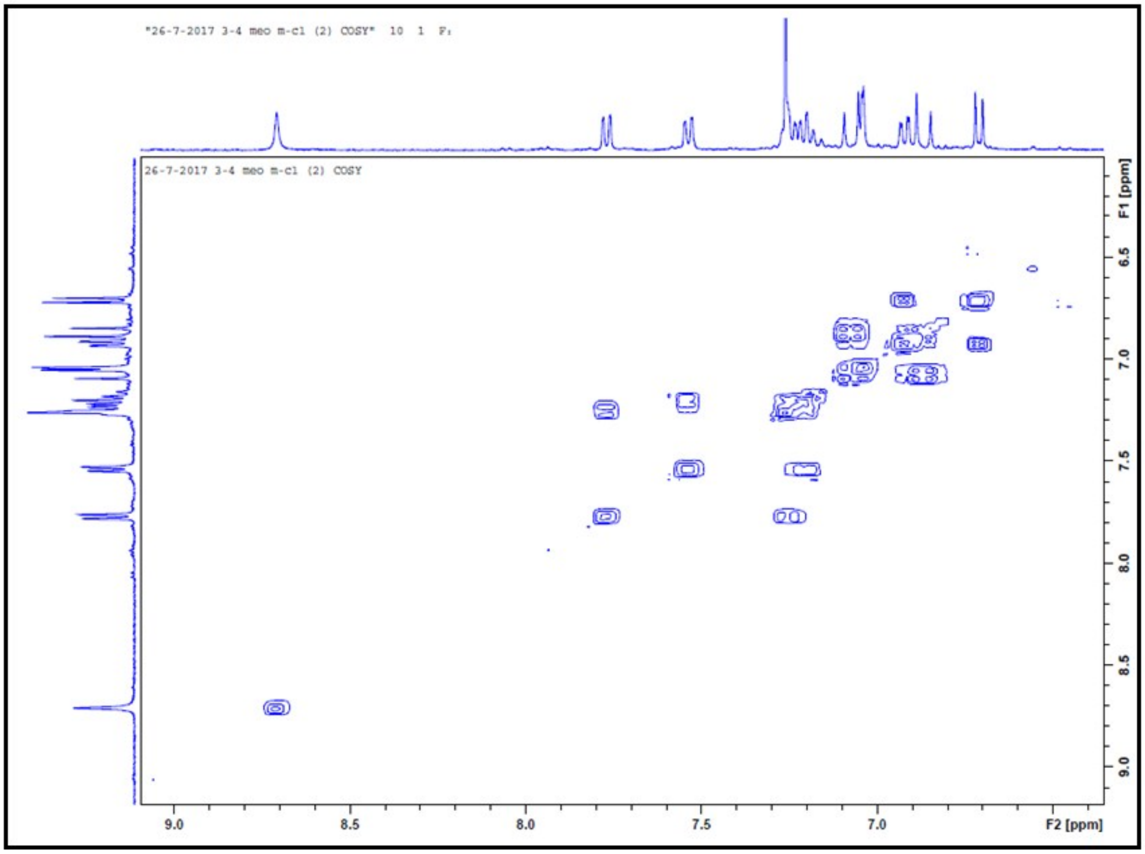

Figure S20: C COSY GPSW {^{1}H}($CDCl_3$, 400MHz) spectrum of compound bis (2-(E))-3,4-dimethoxystyryl)phenyl) malonamide (EXPANSION).

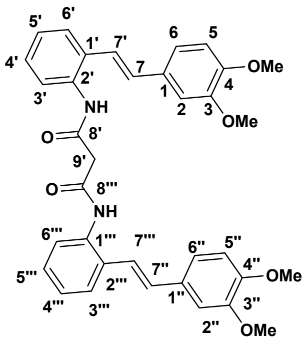

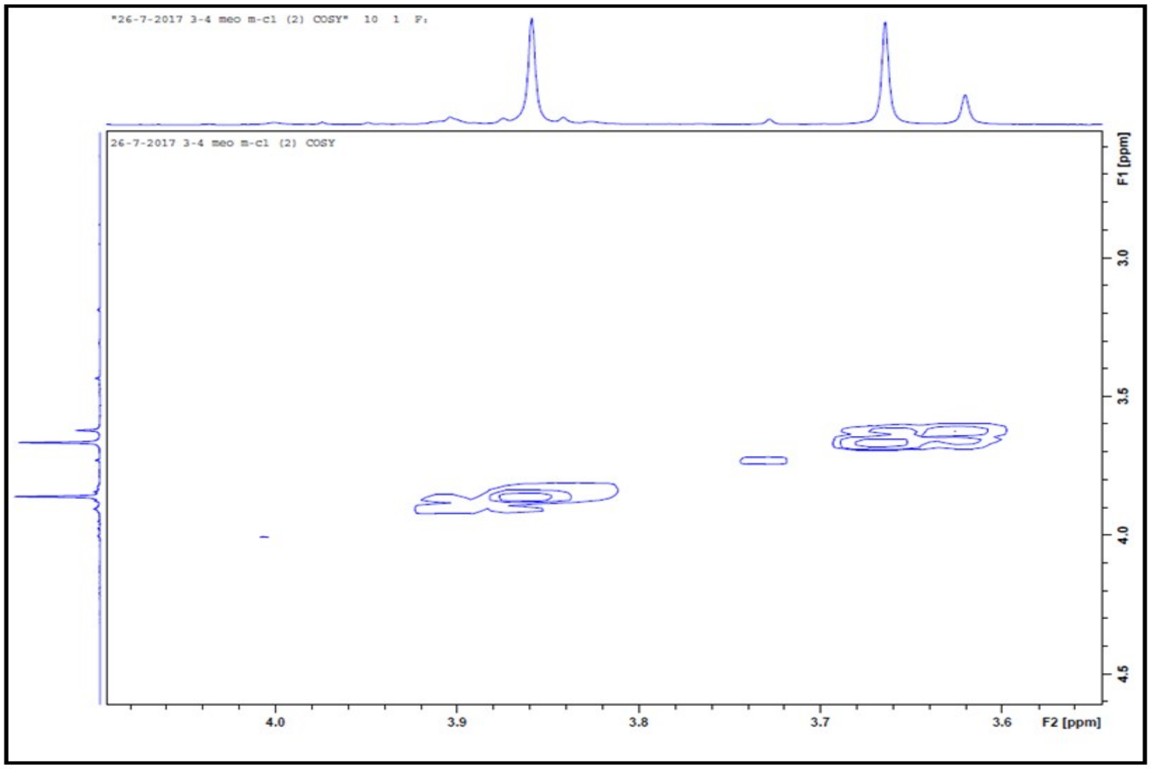

Figure S21: C COSY GPSW {^{1}H}($CDCl_3$, 400MHz) spectrum of compound bis (2-(E))-3,4 dimethoxystyryl)phenyl) malonamide (EXPANSION).

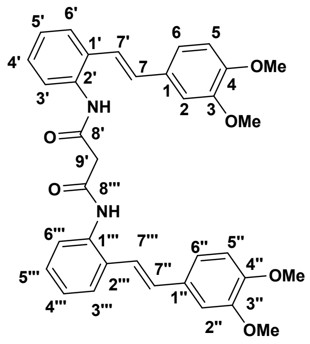

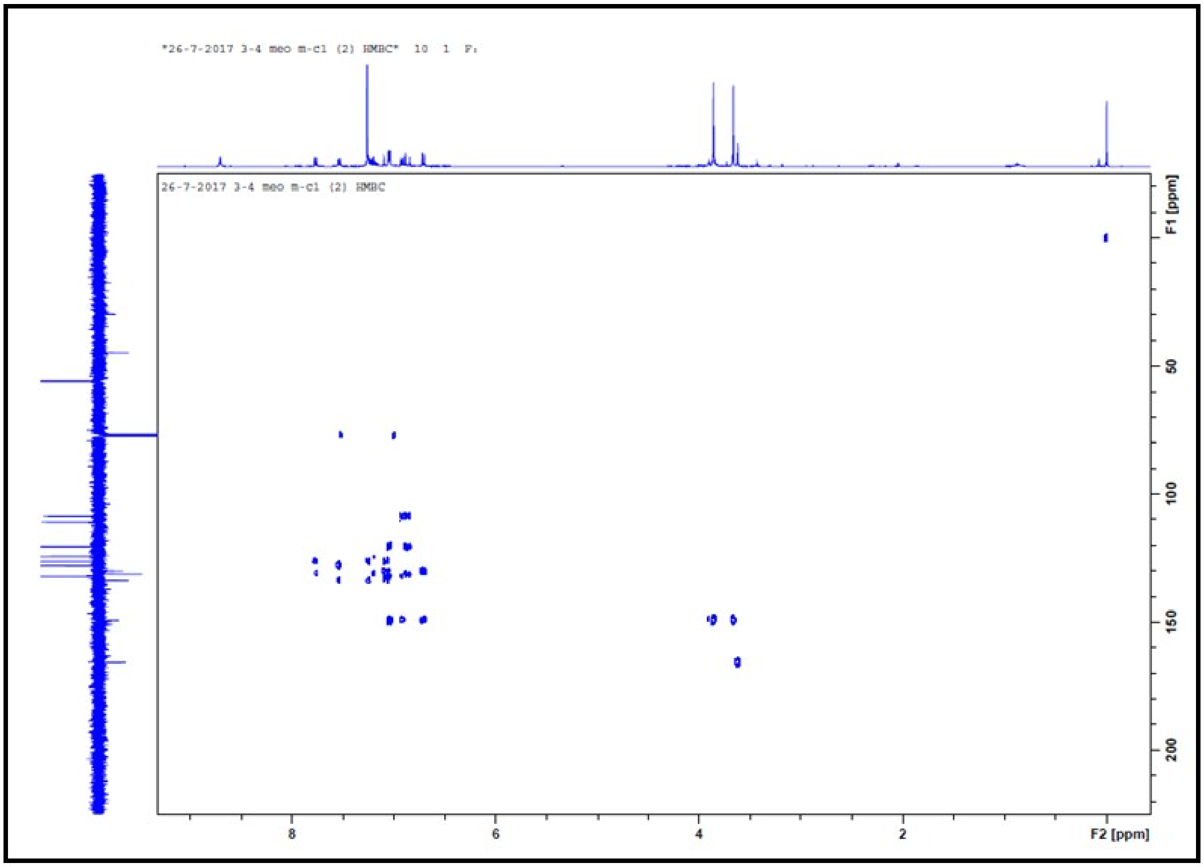

Figure S22: C HMBC GP {^{1}H- ^{13}C}($CDCl_3$, 400MHz) spectrum of compound bis (2-(E))-3,4-dimethoxystyryl)phenyl) malonamide.

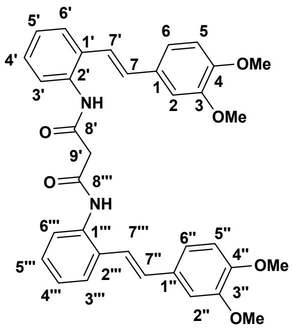

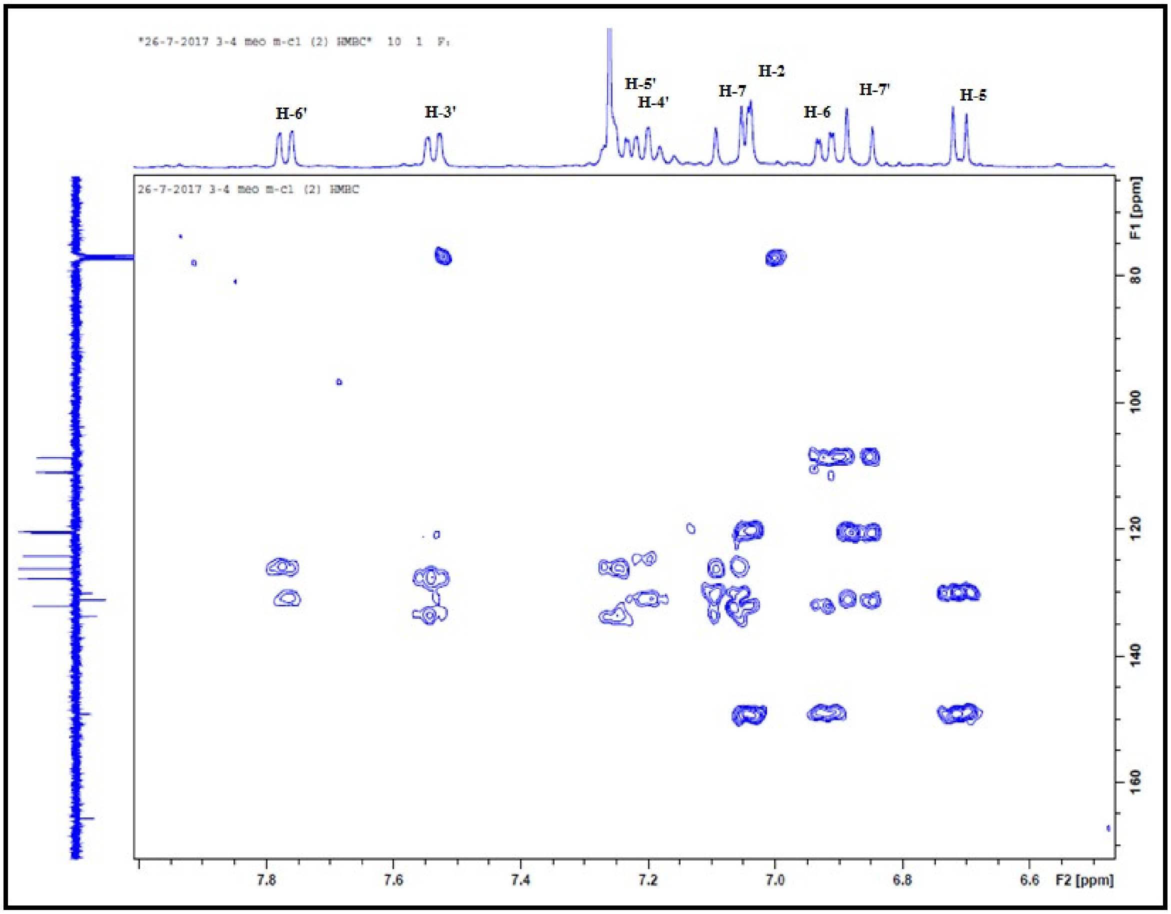

Figure S23: C HMBC GP {^{1}H- ^{13}C}($CDCl_3$, 400MHz) spectrum of compound bis (2-(E))-3,4-dimethoxystyryl)phenyl) malonamide (EXPANSION).

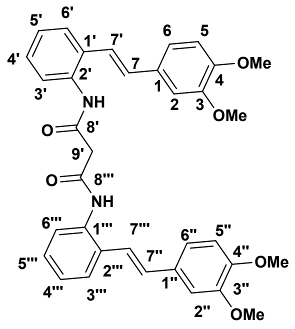

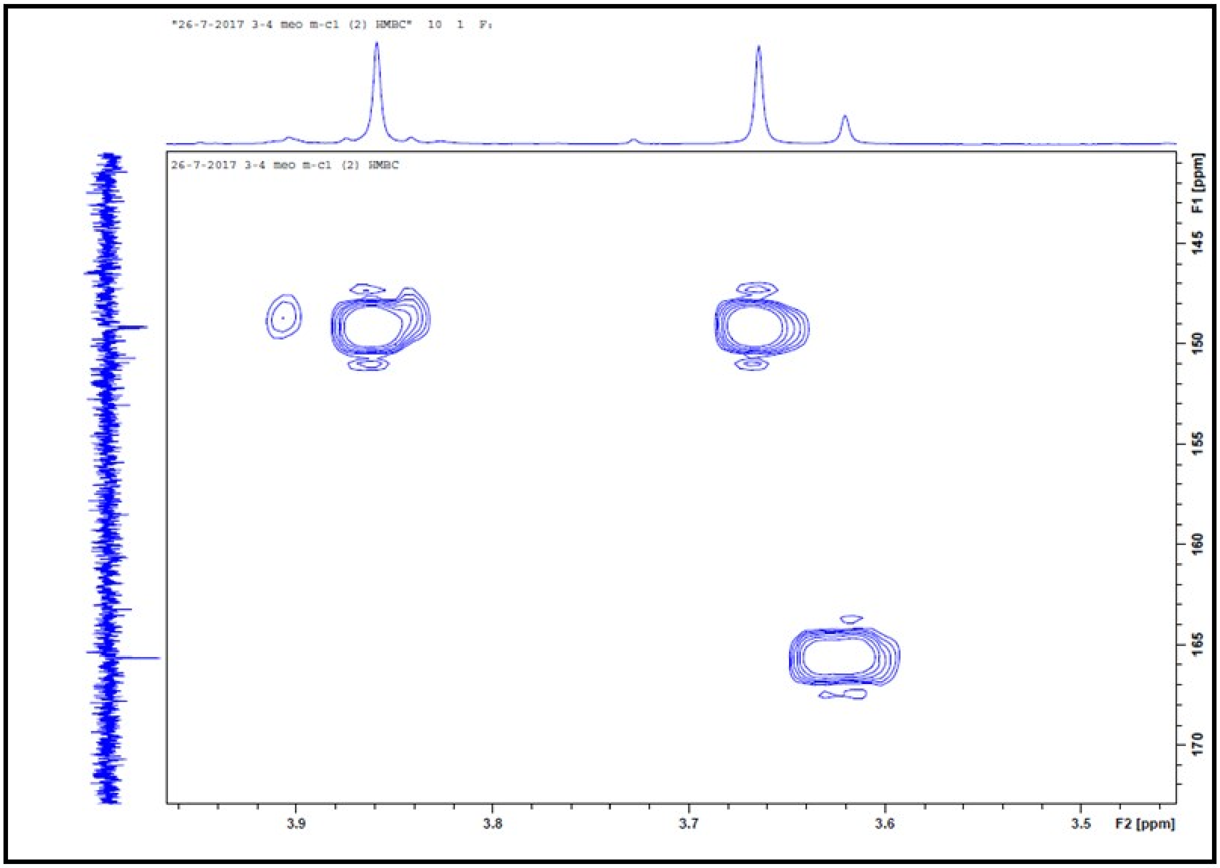

Figure S24: C HMBC GP {^{1}H- ^{13}C}(CDCl$_3$, 400MHz) spectrum of compound bis (2-(E))-3,4-dimethoxystyryl)phenyl) malonamide (EXPANSION).

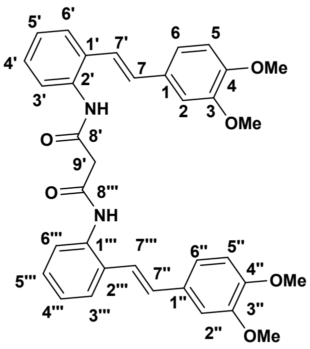

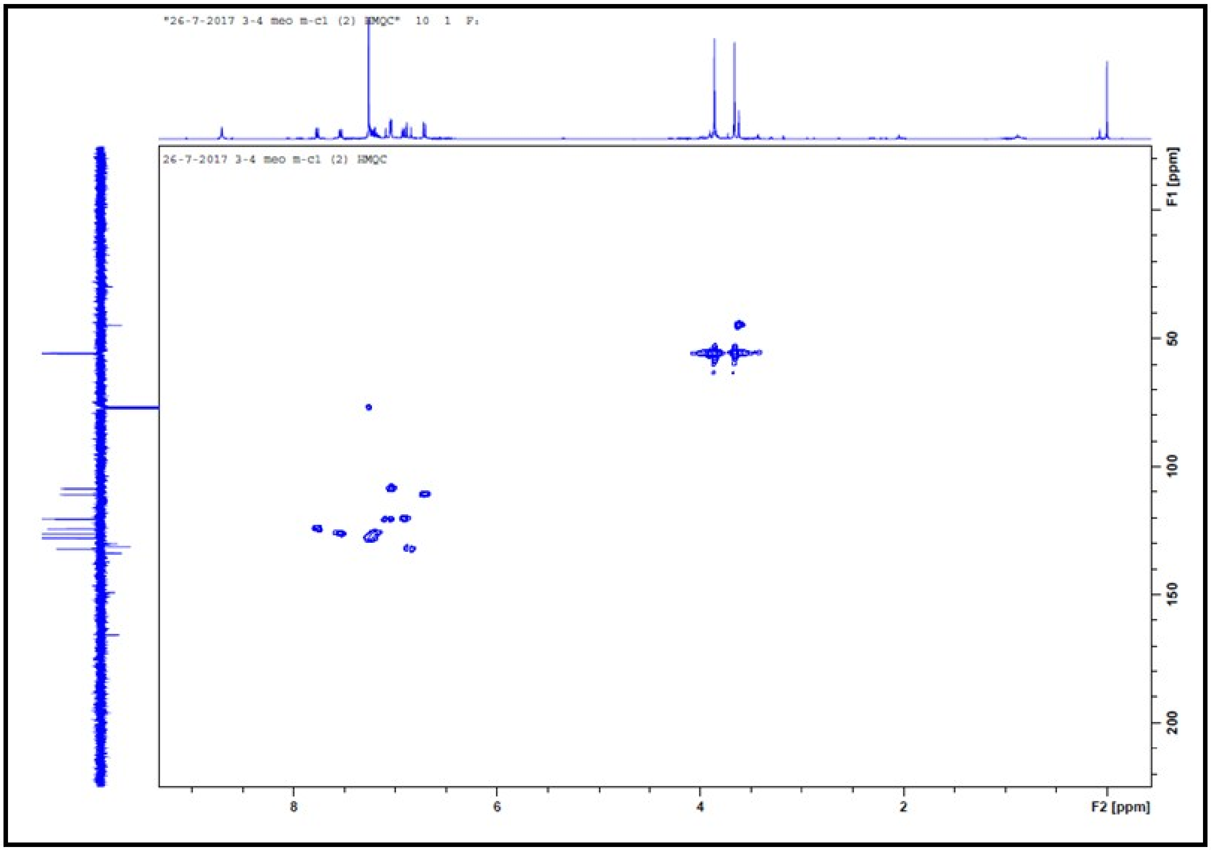

Figure S25: C HMQC GP {^{1}H-^{13}C}($CDCl_3$, 400MHz) spectrum of compound bis (2-(E))-3,4-dimethoxystyryl)phenyl) malonamide.

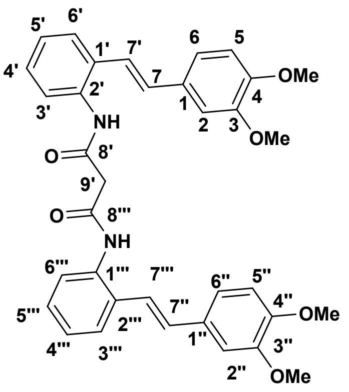

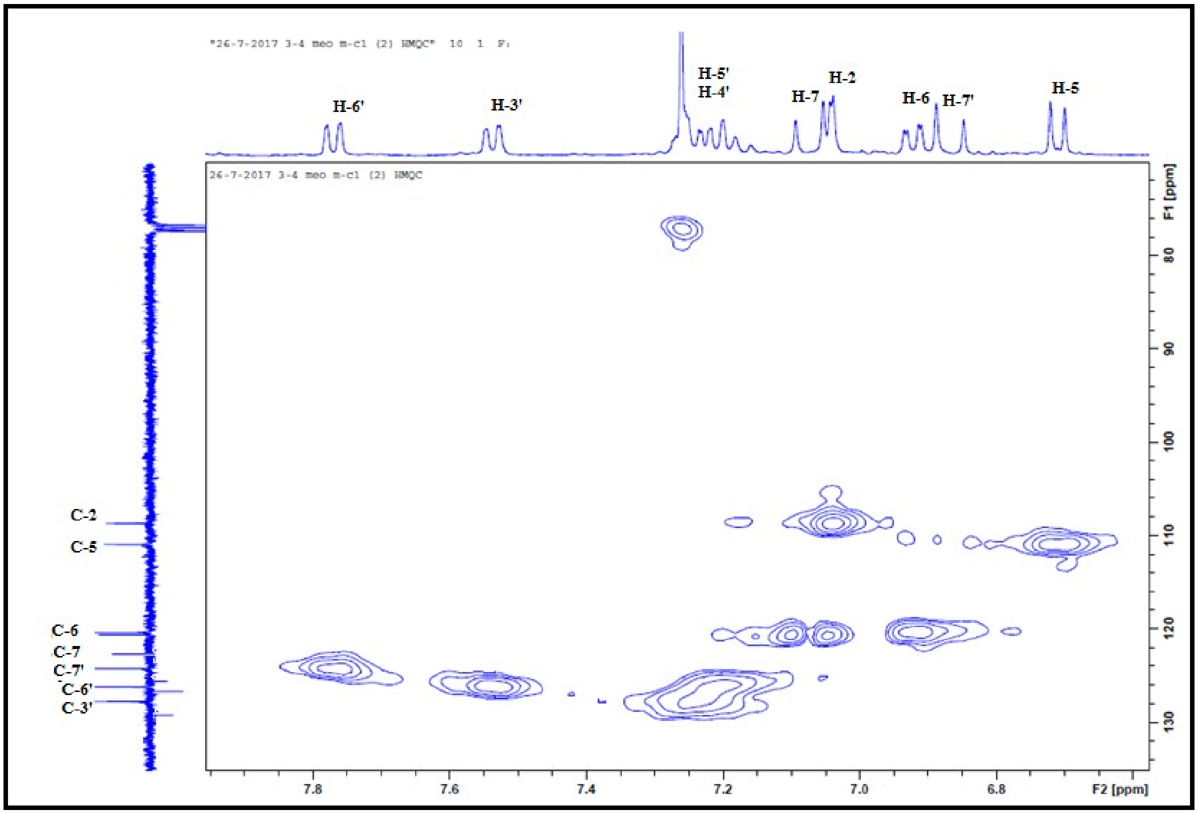

Figure S26: C HMQC GP {^{1}H-^{13}C}($CDCl_3$, 400MHz) spectrum of compound bis (2-(E))-3,4-dimethoxystyryl)phenyl) malonamide (EXPANSION).

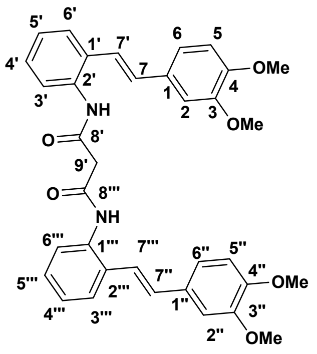

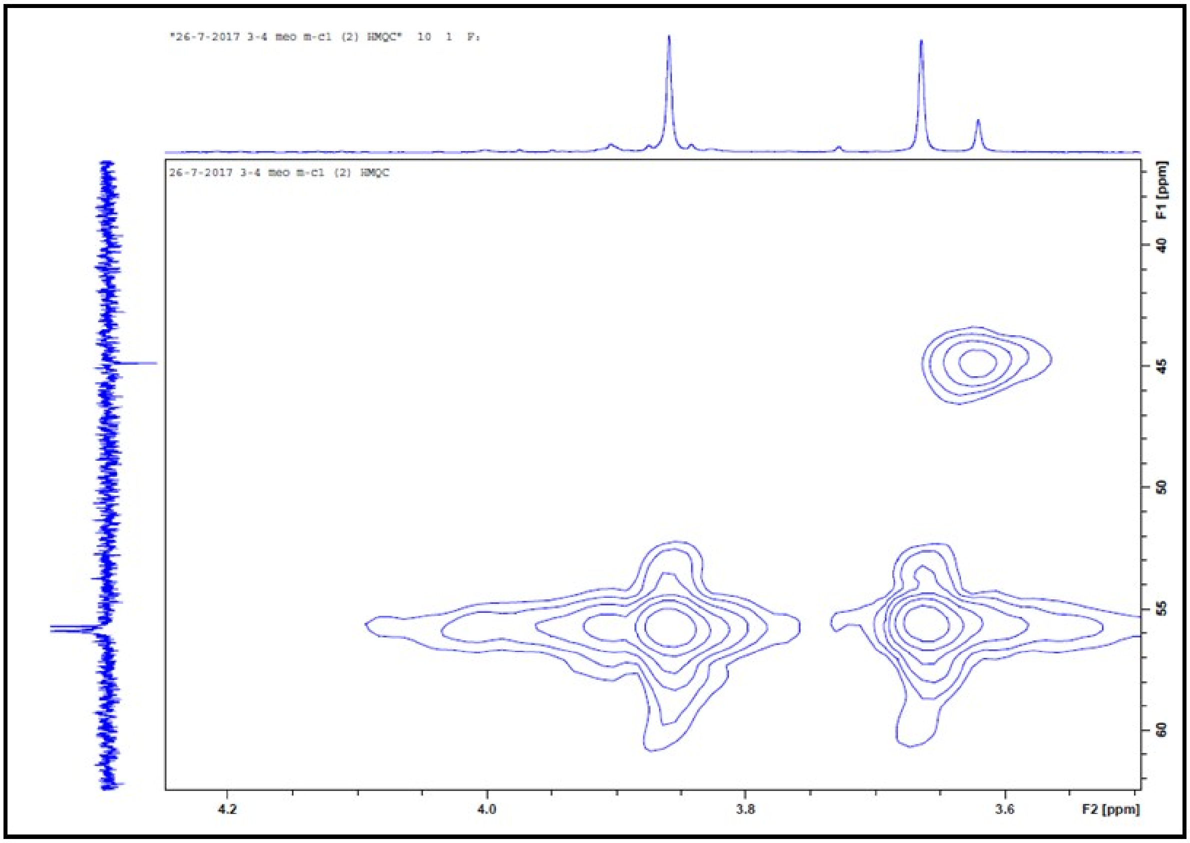

Figure S27: C HMQC GP {^{1}H-^{13}C}(CDCl$_3$, 400MHz) spectrum of compound bis (2-(E))-3,4-dimethoxystyryl)phenyl) malonamide (EXPANSION).

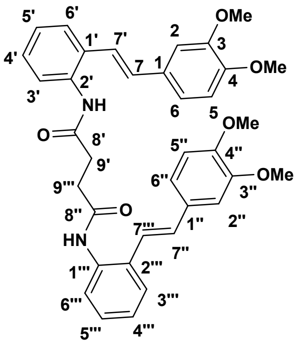

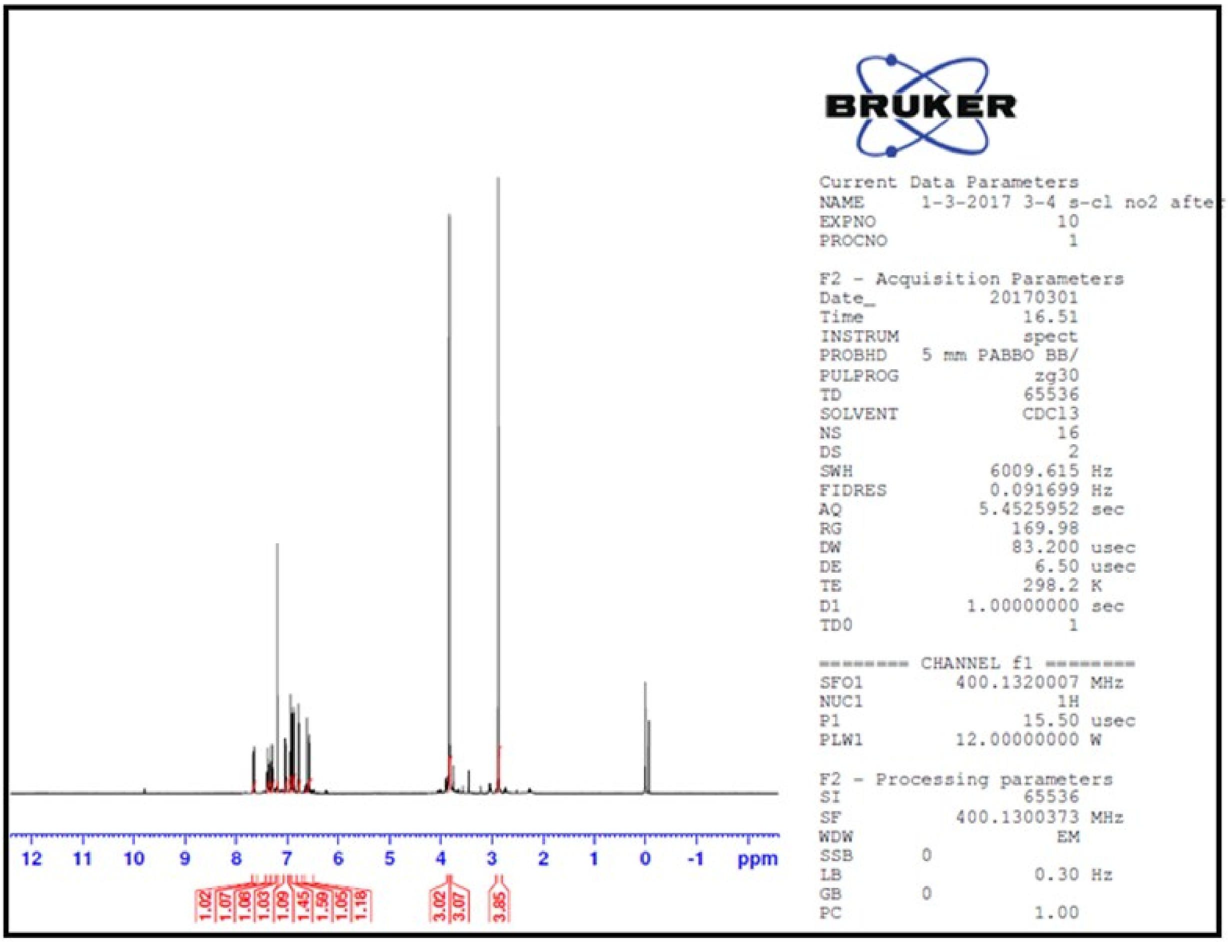

Figer28: ^{1}H NMR ($CDCl_3$, 400MHz) spectrum of compound bis(2((E)-(3,4-dimethoxystyryl)phenyl)succinamide.

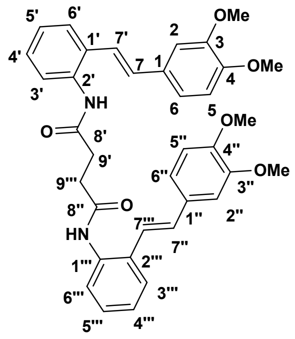

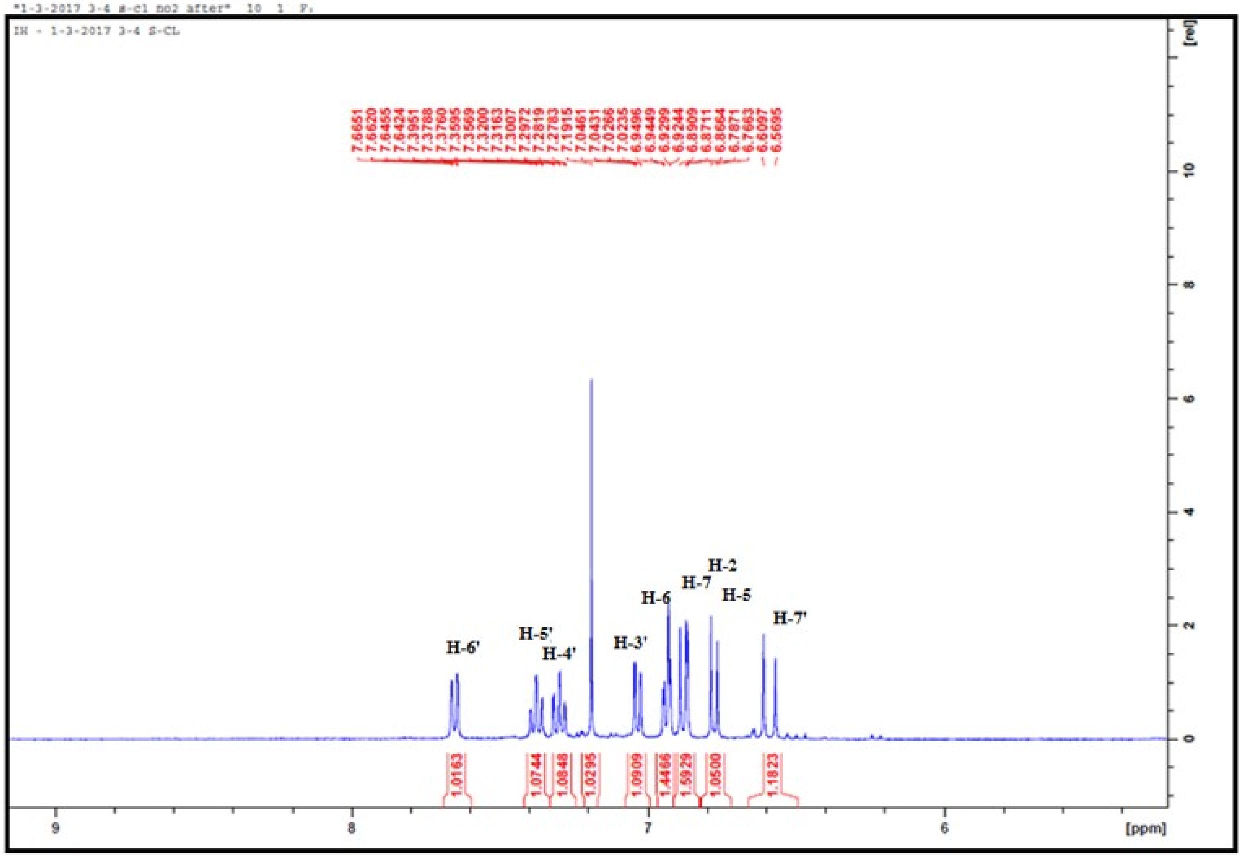

Figure S29: ¹H NMR ($CDCl_3$, 400MHz) spectrum of compound bis(2((E)-(3,4-dimethoxystyryl)phenyl)succinamide (EXPANSION).

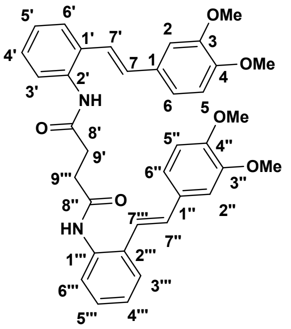

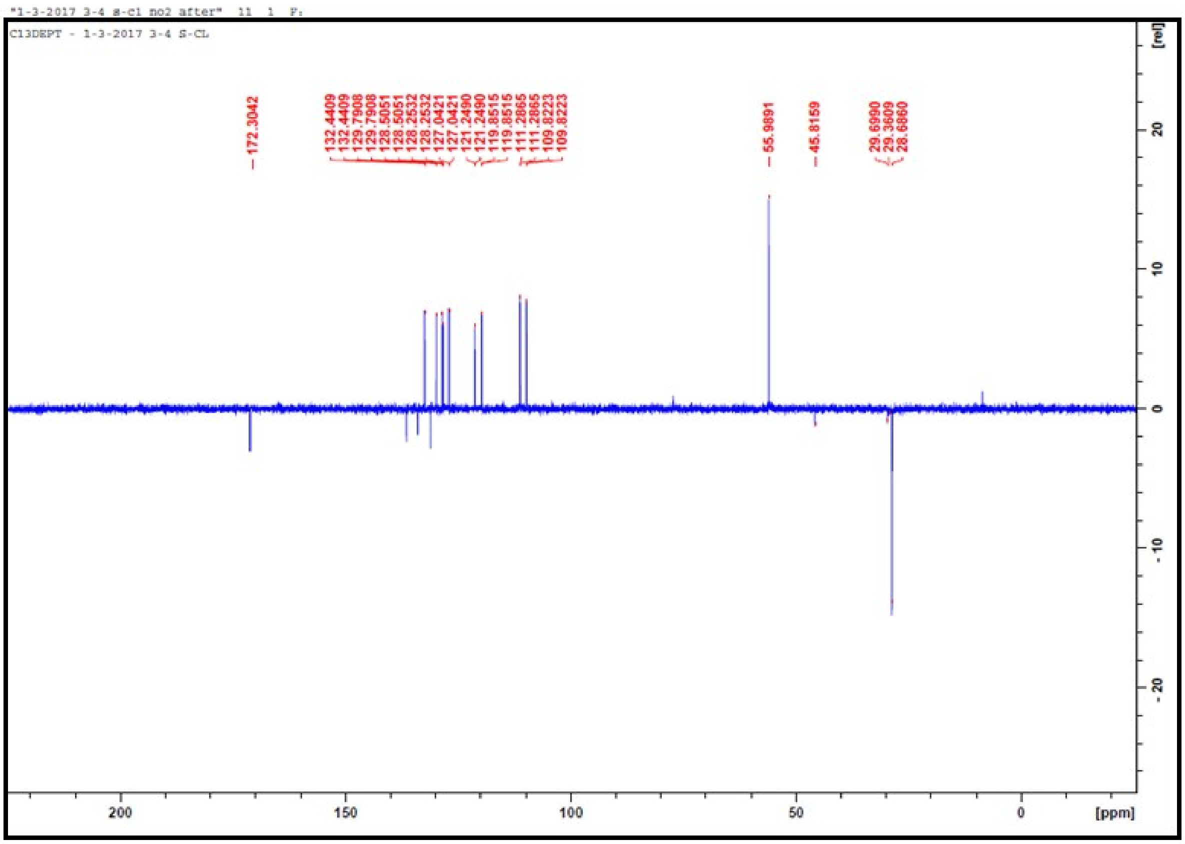

Figure S30: ^{13}C DEPT135,CH_3/CH po {^{1}H}($CDCl_3$, 400MHz) spectrum of compound bis(2((E)-(3,4-dimethoxystyryl)phenyl)succinamide.

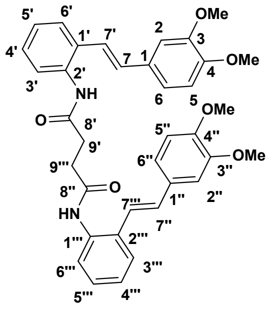

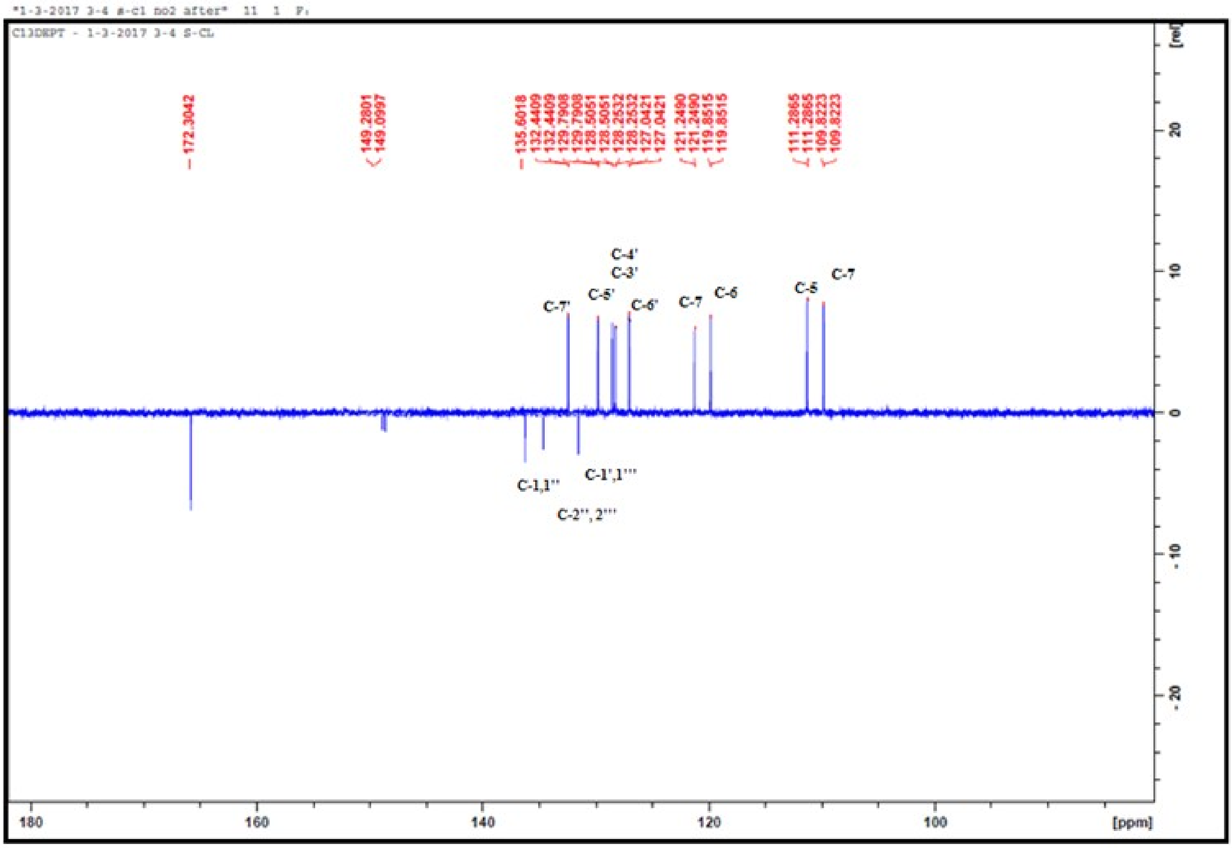

Figure S31: ^{13}C DEPT135,CH_3/CH po {^{1}H}($CDCl_3$, 400MHz) spectrum of compound bis(2((E)-(3,4-dimethoxystyryl)phenyl)succinamide (EXPANSION).

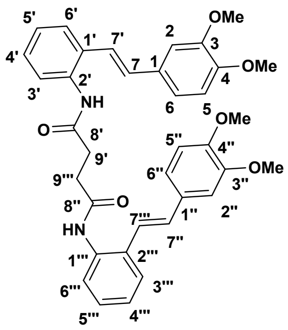

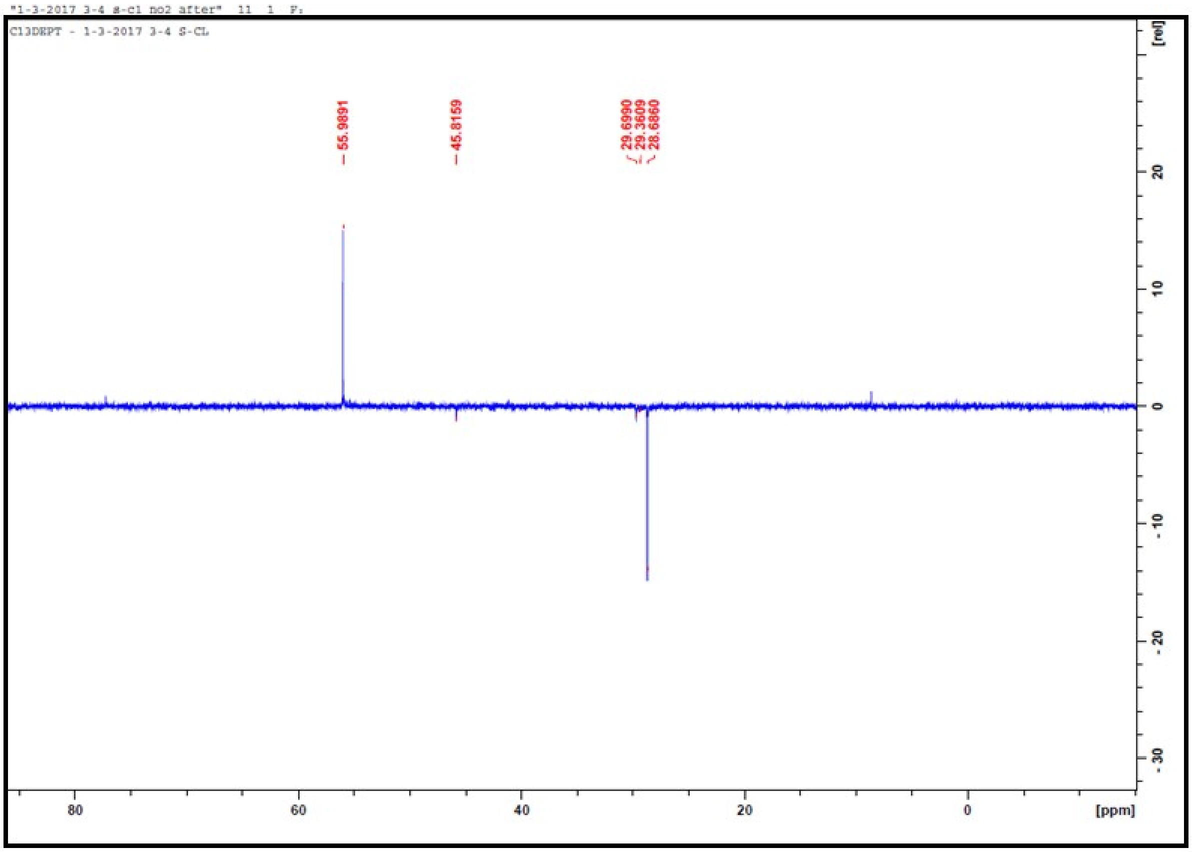

Figure S32: ^{13}C DEPT135,CH_3/CH po {^{1}H}($CDCl_3$, 400MHz) spectrum of compound bis(2((E)-(3,4-dimethoxystyryl)phenyl)succinamide (EXPANSION).

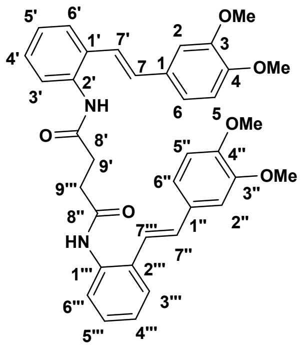

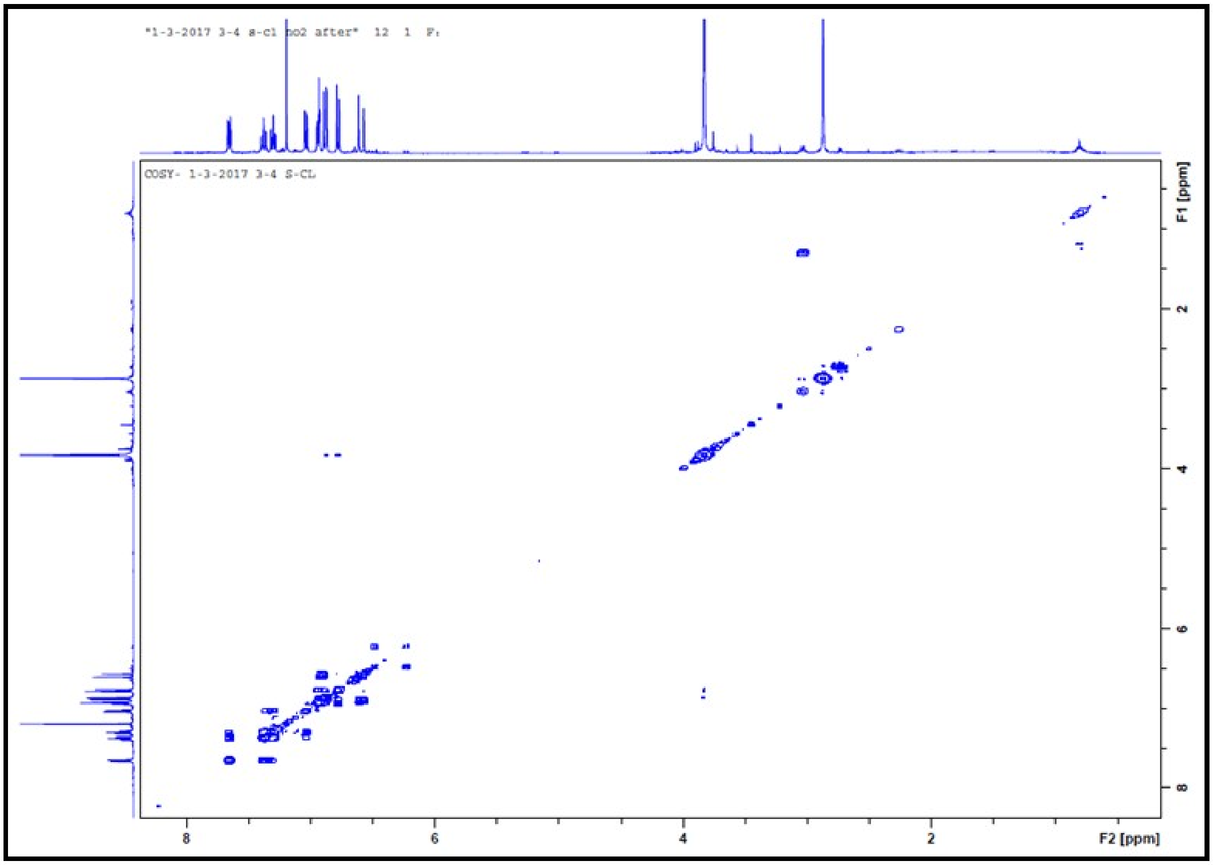

Figure S33: C COSY GPSW {^{1}H}(CDCl$_3$, 400MHz) spectrum of compound bis(2((E)-(3,4-dimethoxystyryl)phenyl)succinamide.

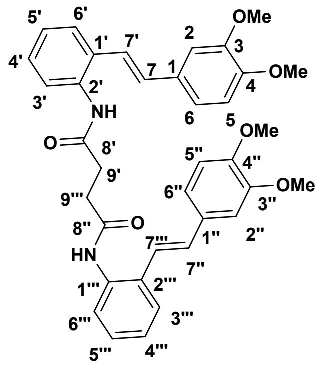

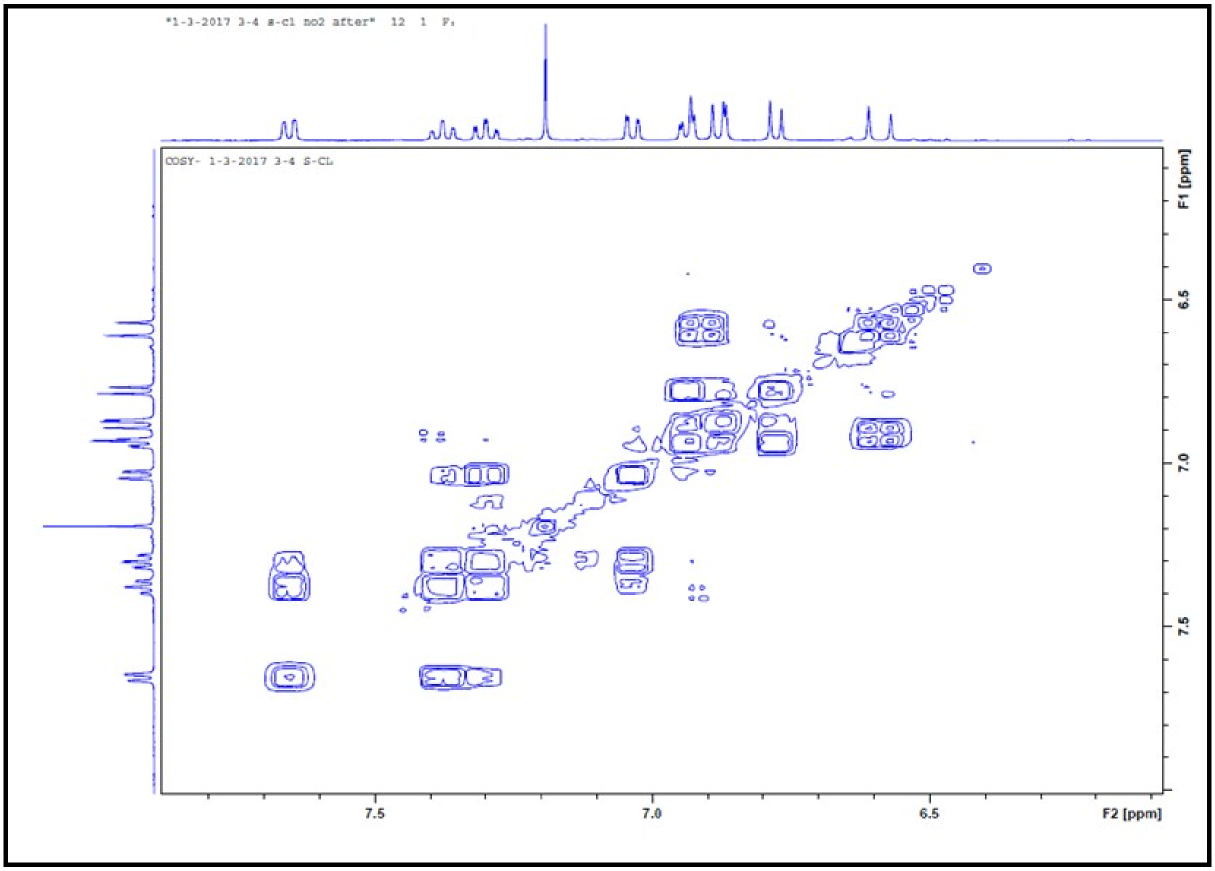

Figure S34: C COSY GPSW {^{1}H}($CDCl_3$, 400MHz) spectrum of compound bis(2((E)-(3,4-dimethoxystyryl)phenyl)succinamide (EXPANSION).

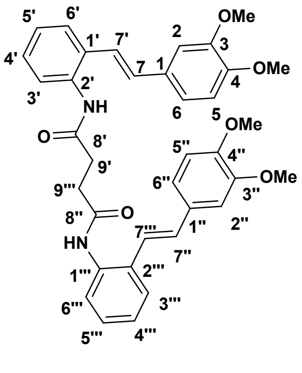

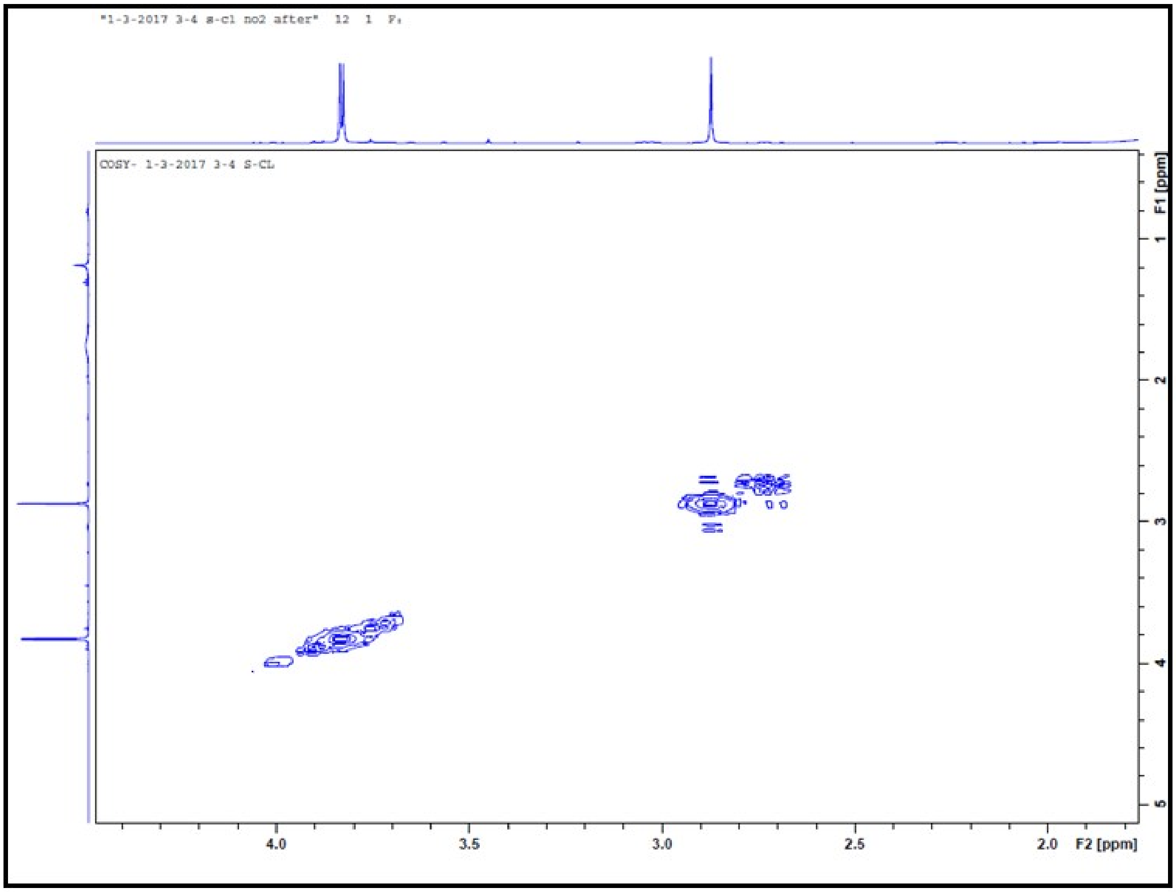

Figure S35: C COSY GPSW {^{1}H}(CDCl$_3$, 400MHz) spectrum of compound bis(2((E)-(3,4-dimethoxystyryl)phenyl)succinamide (EXPANSION).

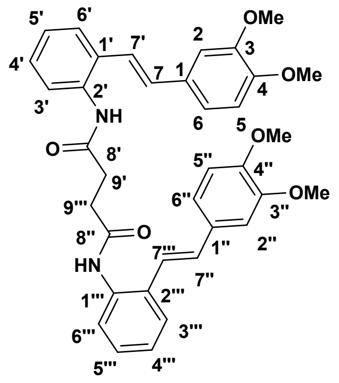

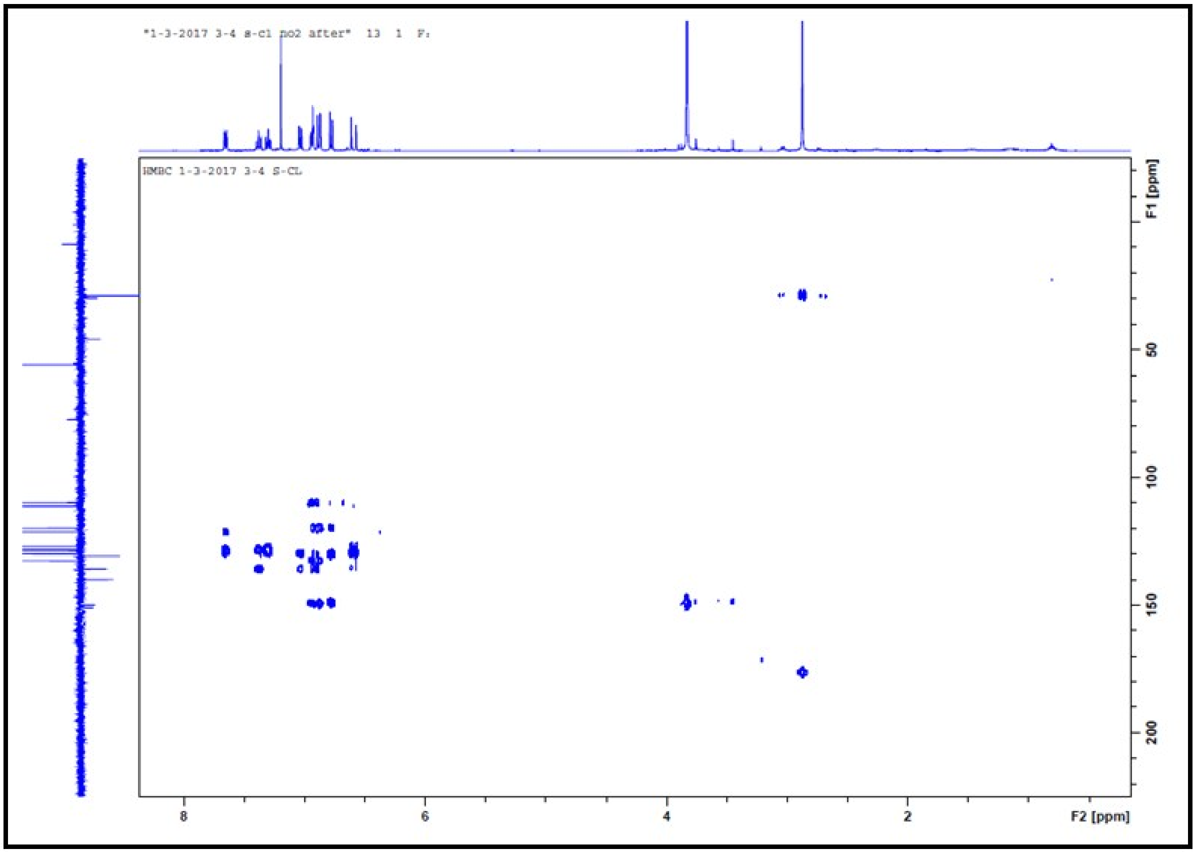

Figure S36: C HMBC GP {^{1}H- ^{13}C}($CDCl_3$, 400MHz) spectrum of compound bis(2((E)-(3,4-dimethoxystyryl)phenyl)succinamide.

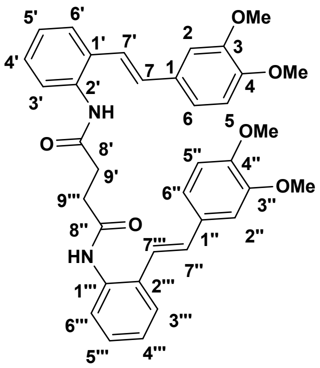

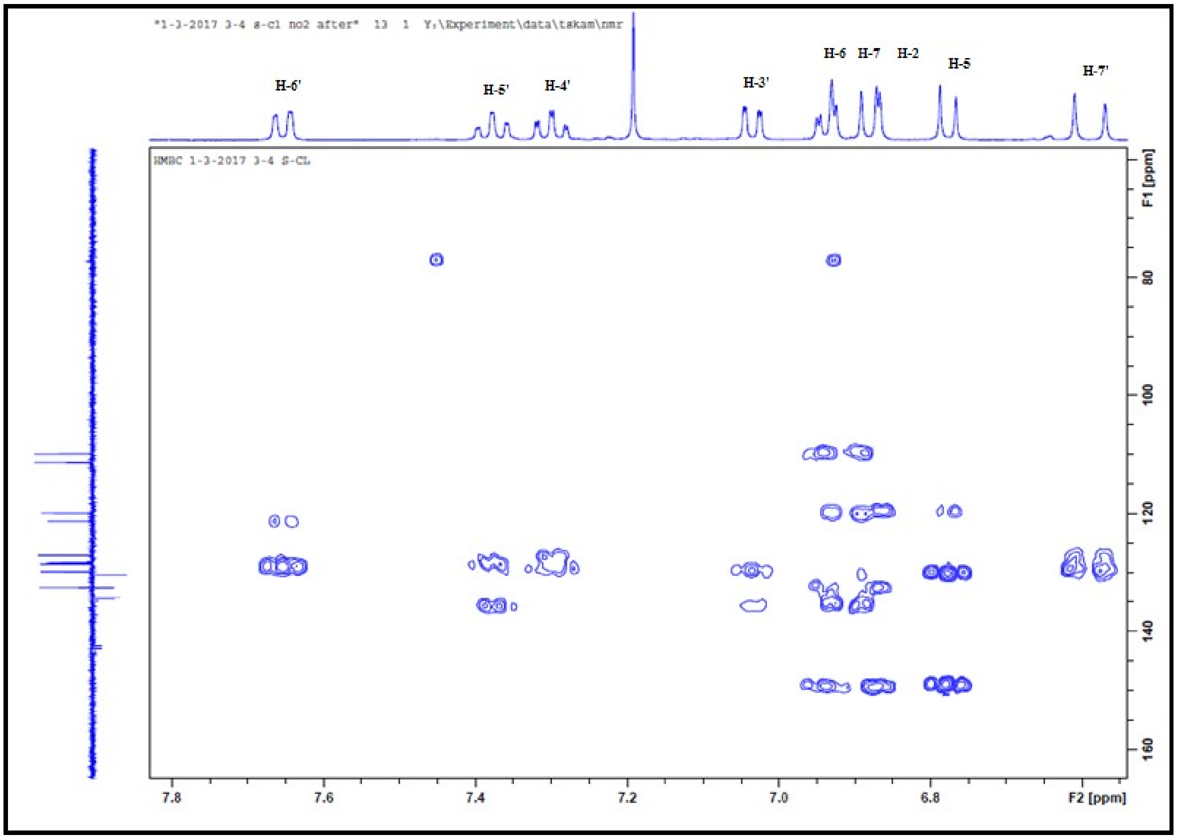

Figure S37: C HMBC GP {^{1}H- ^{13}C}($CDCl_3$, 400MHz) spectrum of compound bis(2((E)-(3,4-dimethoxystyryl)phenyl)succinamide (EXPANSION).

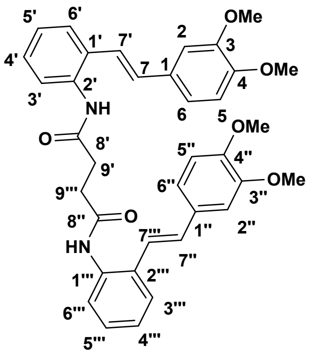

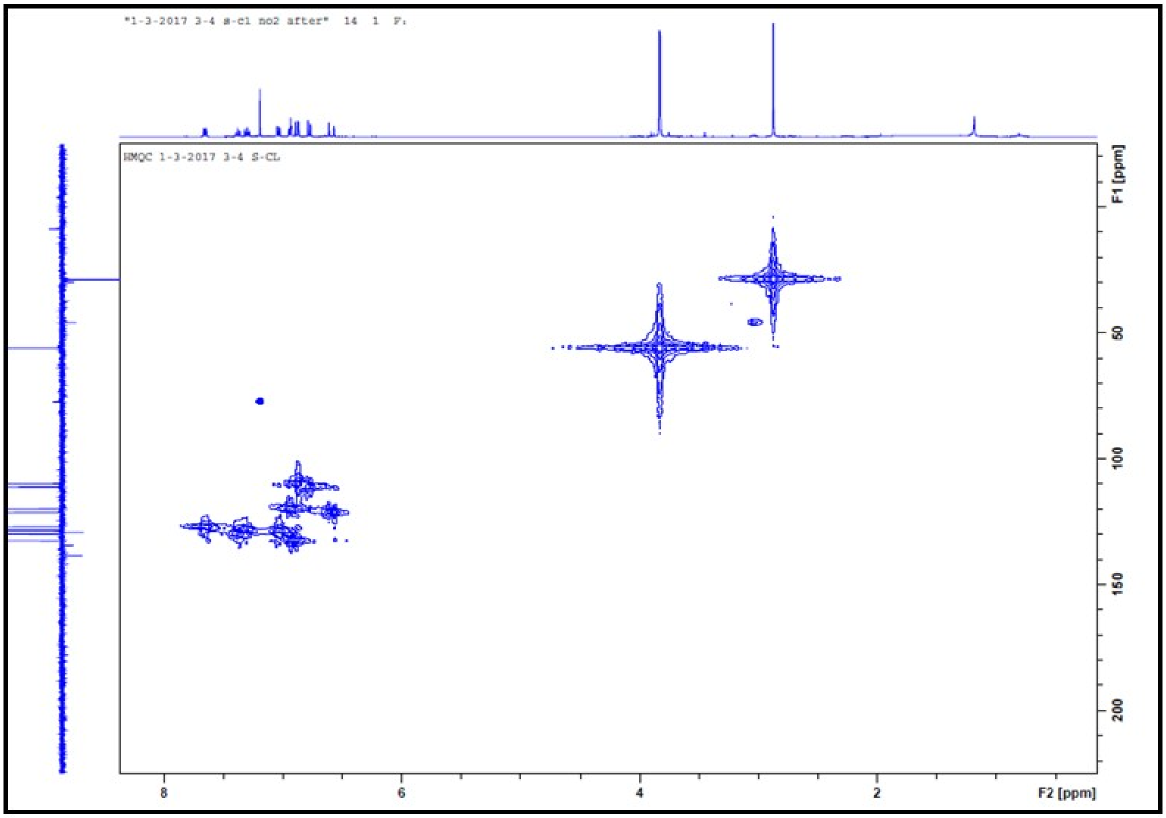

Figure S38: C HMQC GP {^{1}H-^{13}C}($CDCl_3$, 400MHz) spectrum of compound bis(2((E)-(3,4-dimethoxystyryl)phenyl)succinamide.

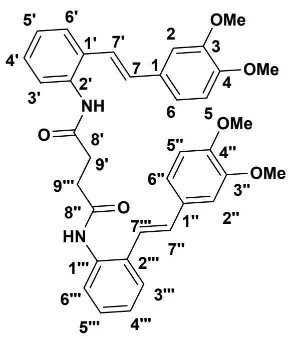

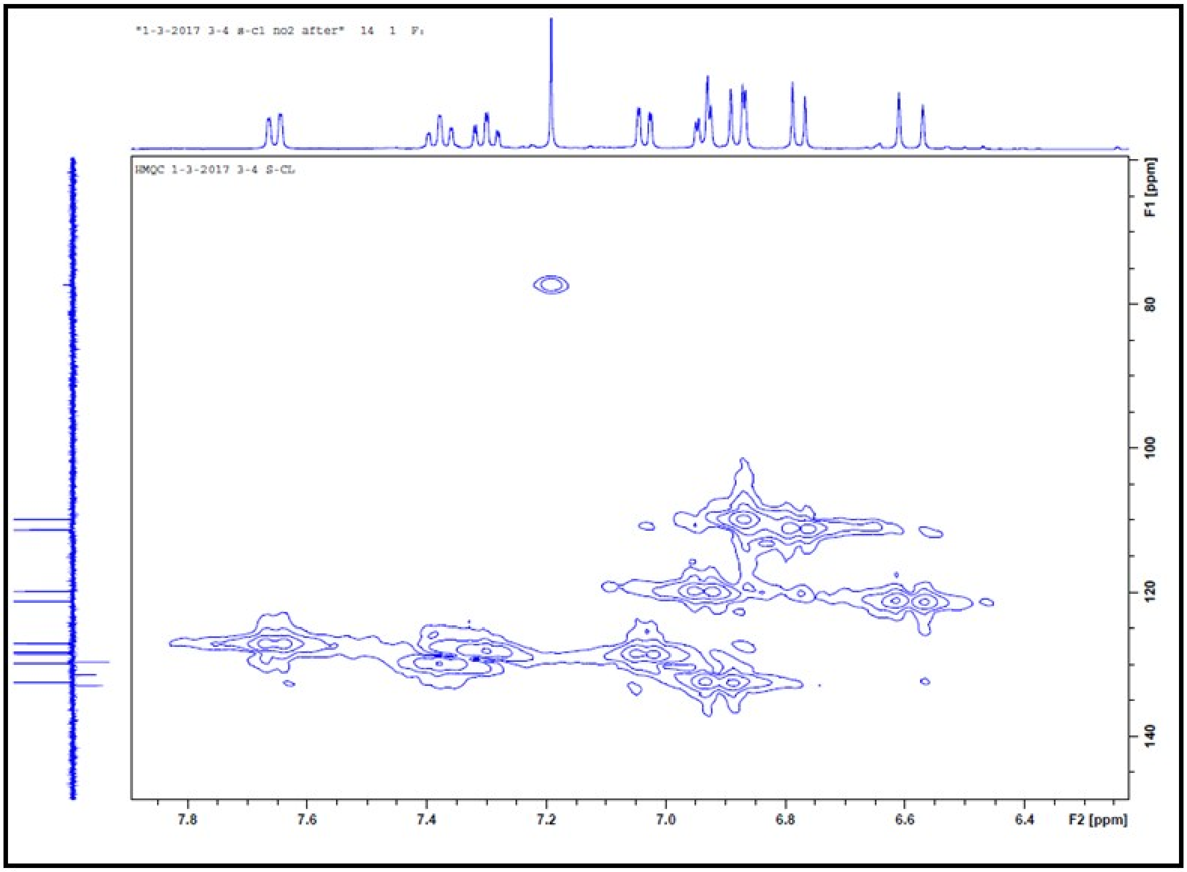

Figure S39: C HMQC GP {^{1}H-^{13}C}($CDCl_3$, 400MHz) spectrum of compound bis(2((E)-(3,4-dimethoxystyryl)phenyl)succinamide (EXPANSION).

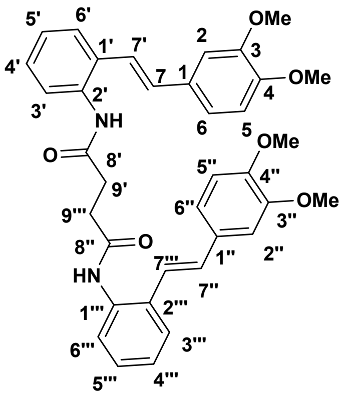

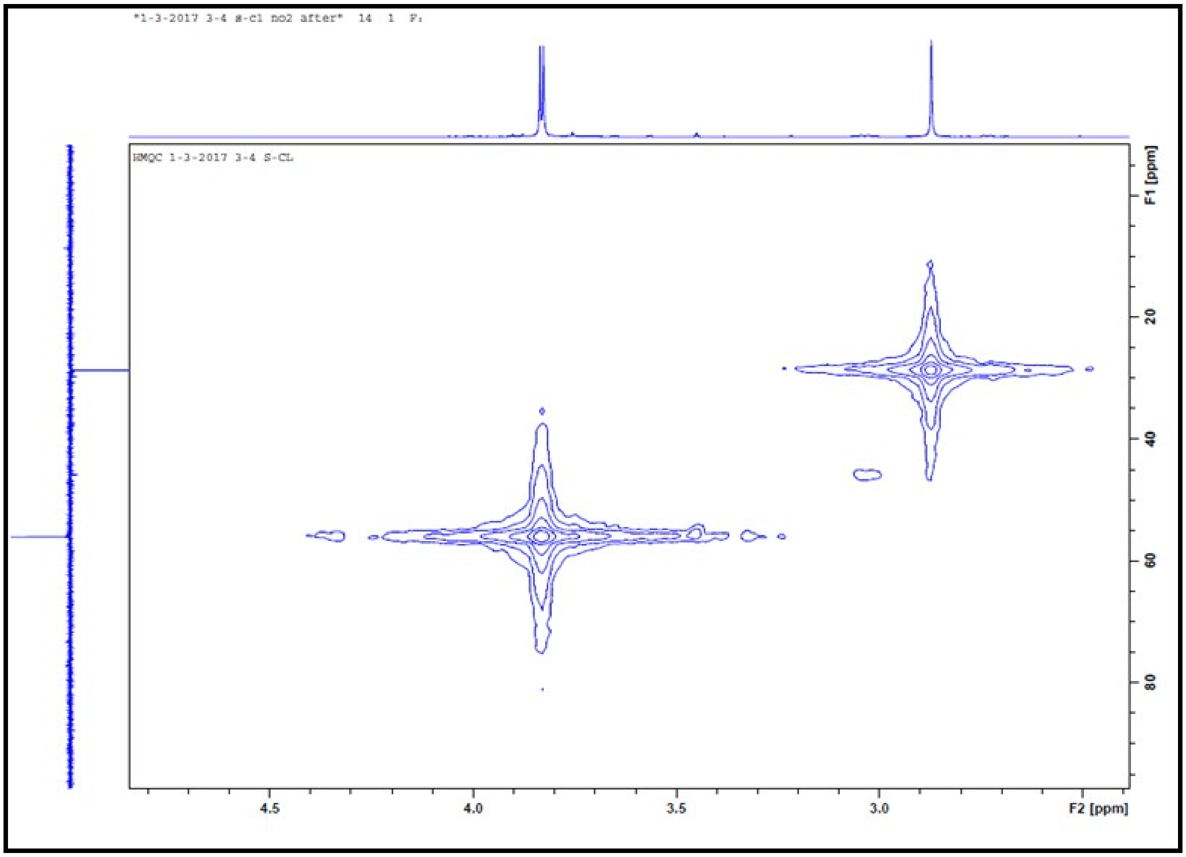

Figure S40: C HMQC GP {^{1}H-^{13}C}($CDCl_3$, 400MHz) spectrum of compound bis(2((E)-(3,4-dimethoxystyryl)phenyl)succinamide (EXPANSION).

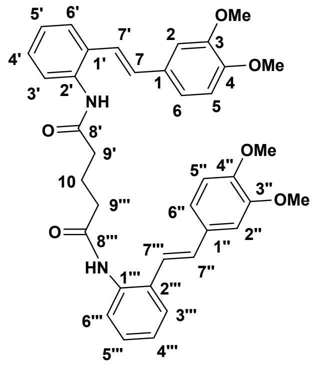

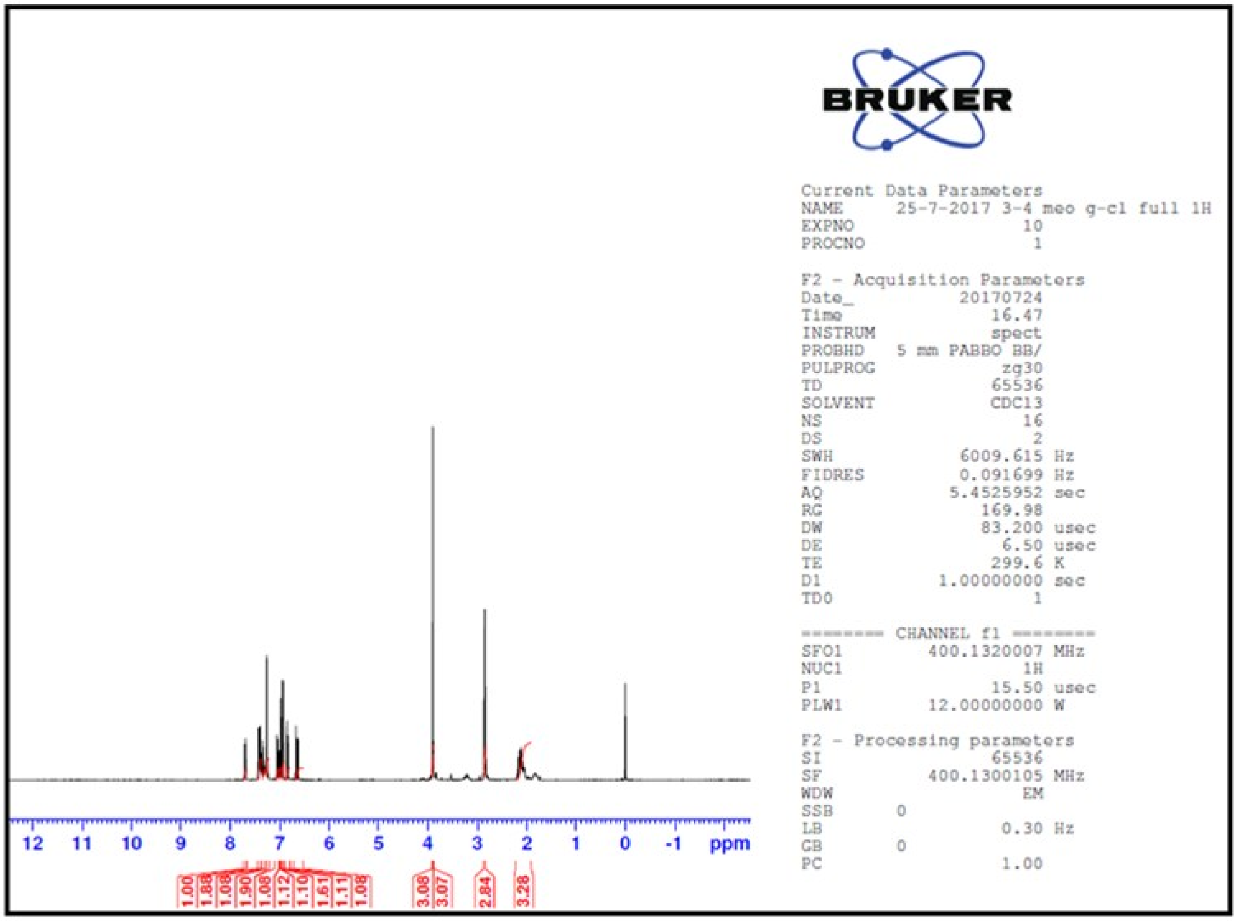

Figure S41: ^{1}H NMR ($CDCl_3$, 400MHz) spectrum of compound bis(2((E)-(3,4-dimethoxystyryl)phenyl) glutaramide.

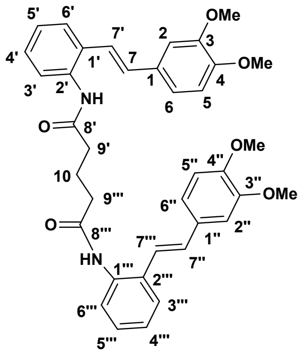

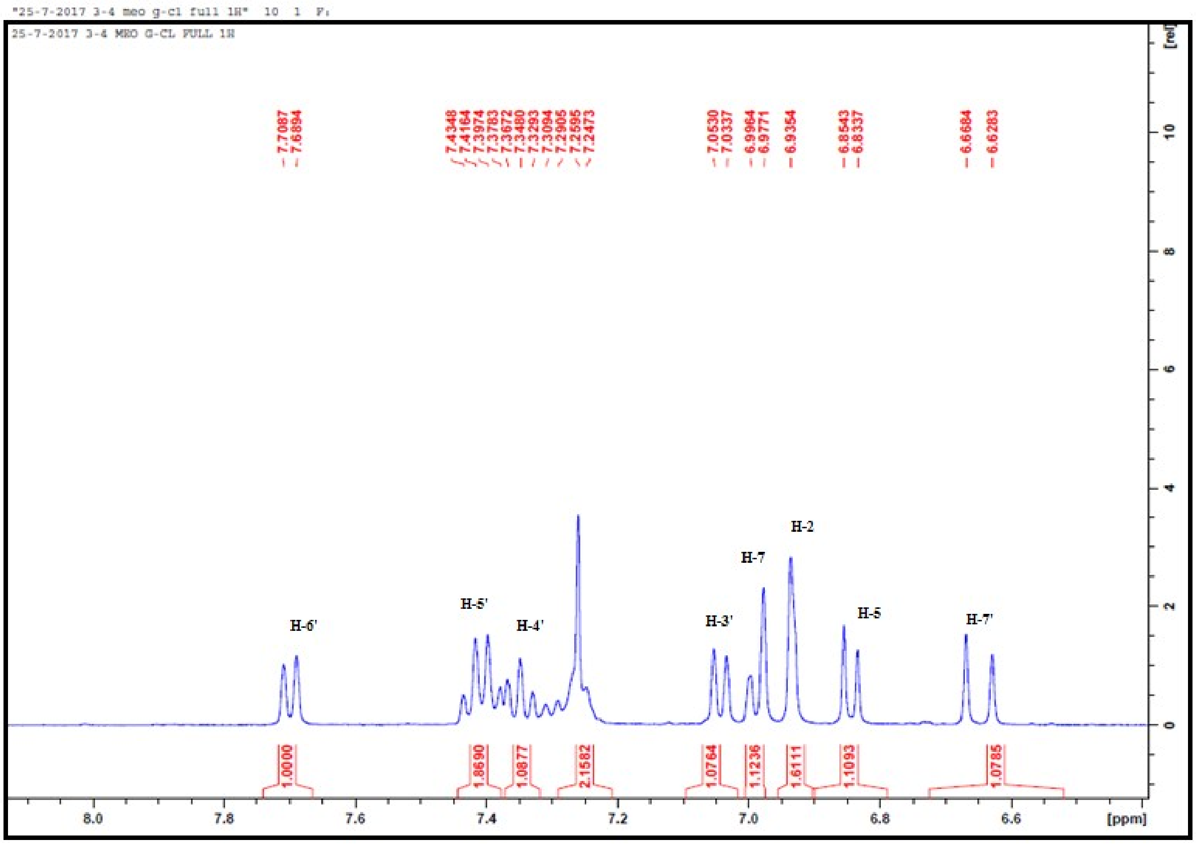

Figure S42: ^{1}H NMR ($CDCl_3$, 400MHz) spectrum of compound bis(2((E)-(3,4-dimethoxystyryl)phenyl) glutaramide (EXPANSION).

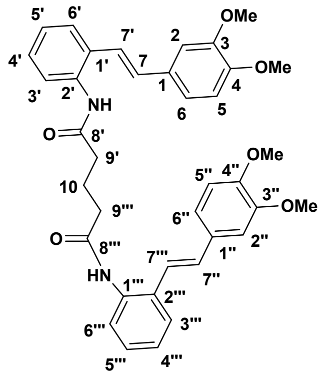

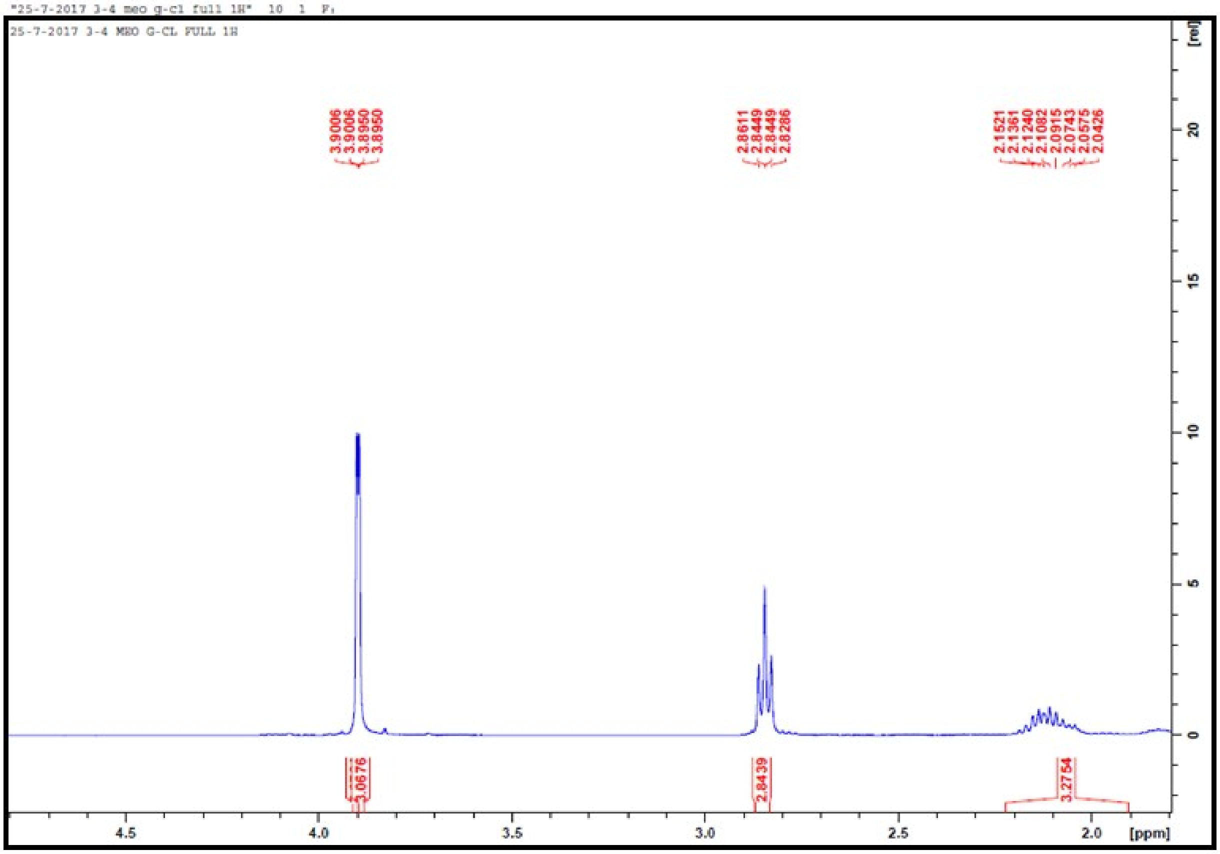

Figure S43: ^{1}H NMR ($CDCl_3$, 400MHz) spectrum of compound bis(2((E)-(3,4-dimethoxystyryl)phenyl) glutaramide (EXPANSION).

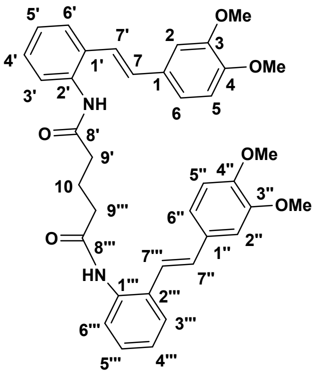

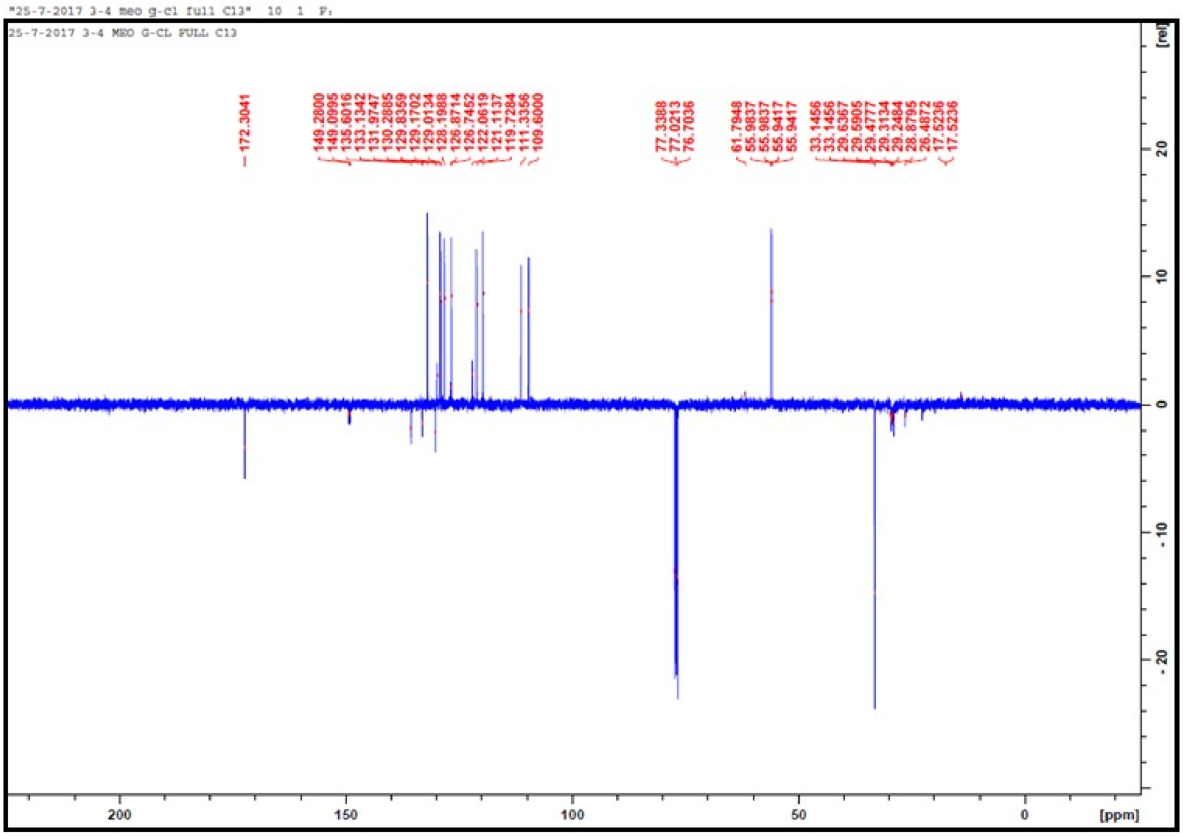

Figure S44: ^{13}C DEPT135,CH_3/CH po {^{1}H}($CDCl_3$, 400MHz) spectrum of compound bis(2((E)-(3,4-dimethoxystyryl)phenyl) glutaramide.

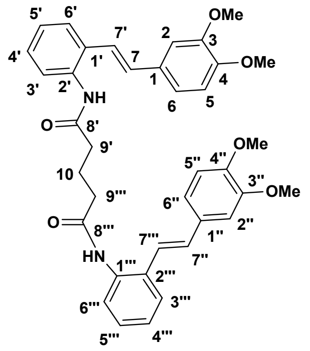

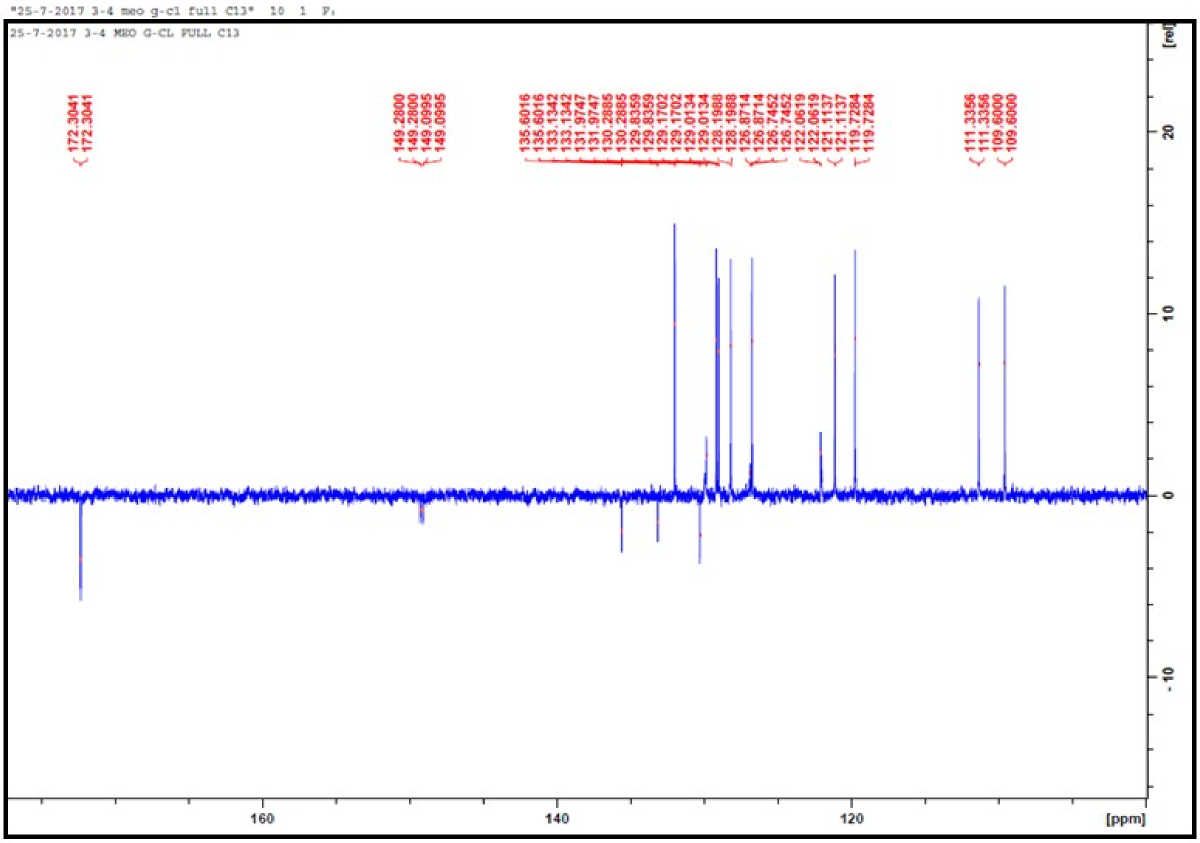

Figure S45: ^{13}C DEPT135,CH_3/CH po {^{1}H}($CDCl_3$, 400MHz) spectrum of compound bis(2((E)-(3,4-dimethoxystyryl)phenyl) glutaramide (EXPANSION).

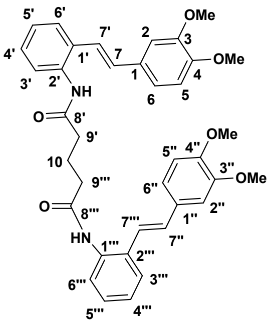

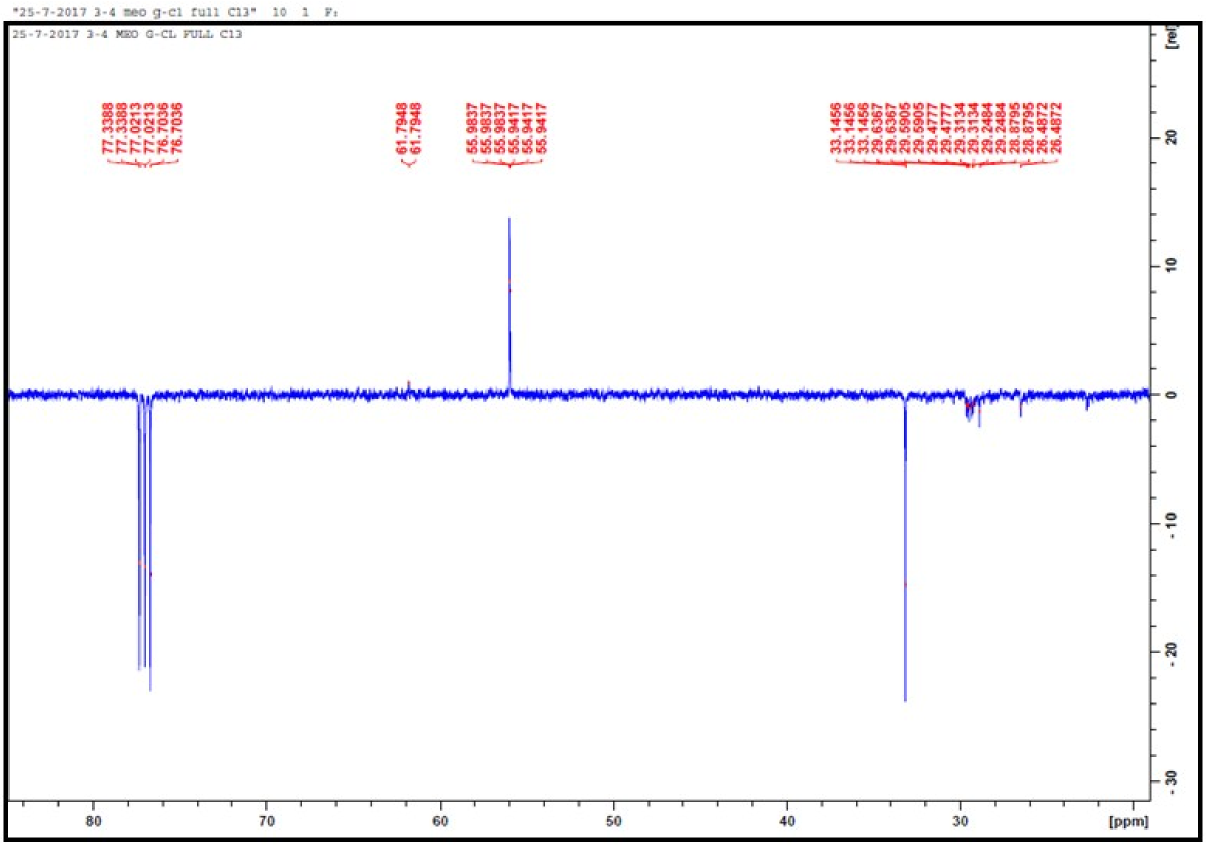

Figure S46: ^{13}C DEPT135,CH$_3$/CH po {^{1}H}(CDCl$_3$, 400MHz) spectrum of compound bis(2((E)-(3,4-dimethoxystyryl)phenyl) glutaramide (EXPANSION).

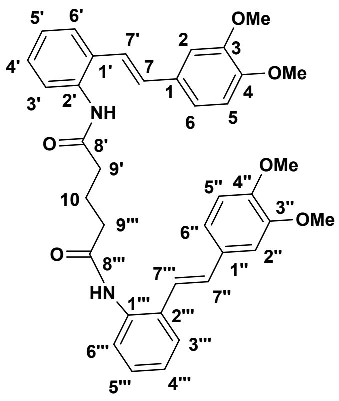

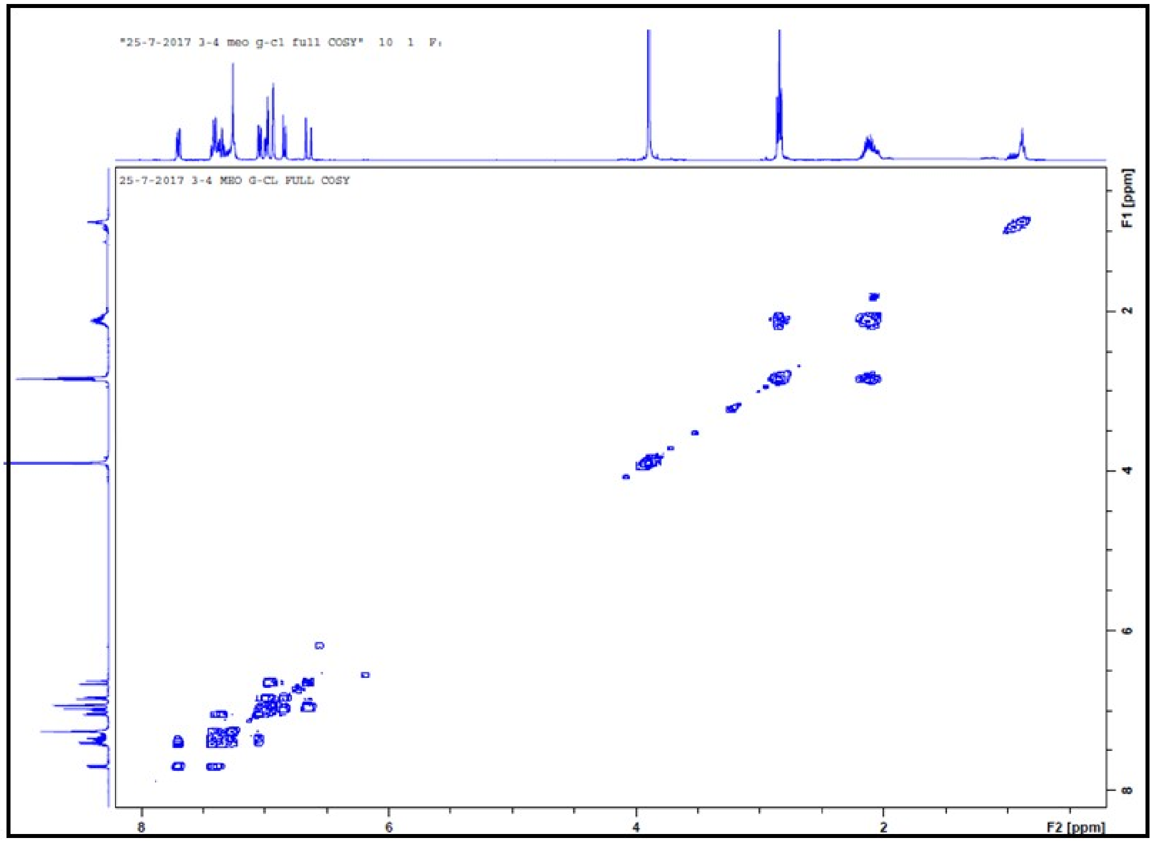

Figure S47: C COSY GPSW {^{1}H}($CDCl_3$, 400MHz) spectrum of compound bis(2((E)-(3,4-dimethoxystyryl)phenyl) glutaramide.

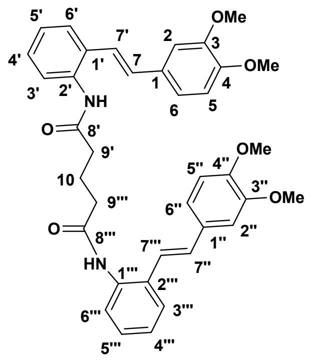

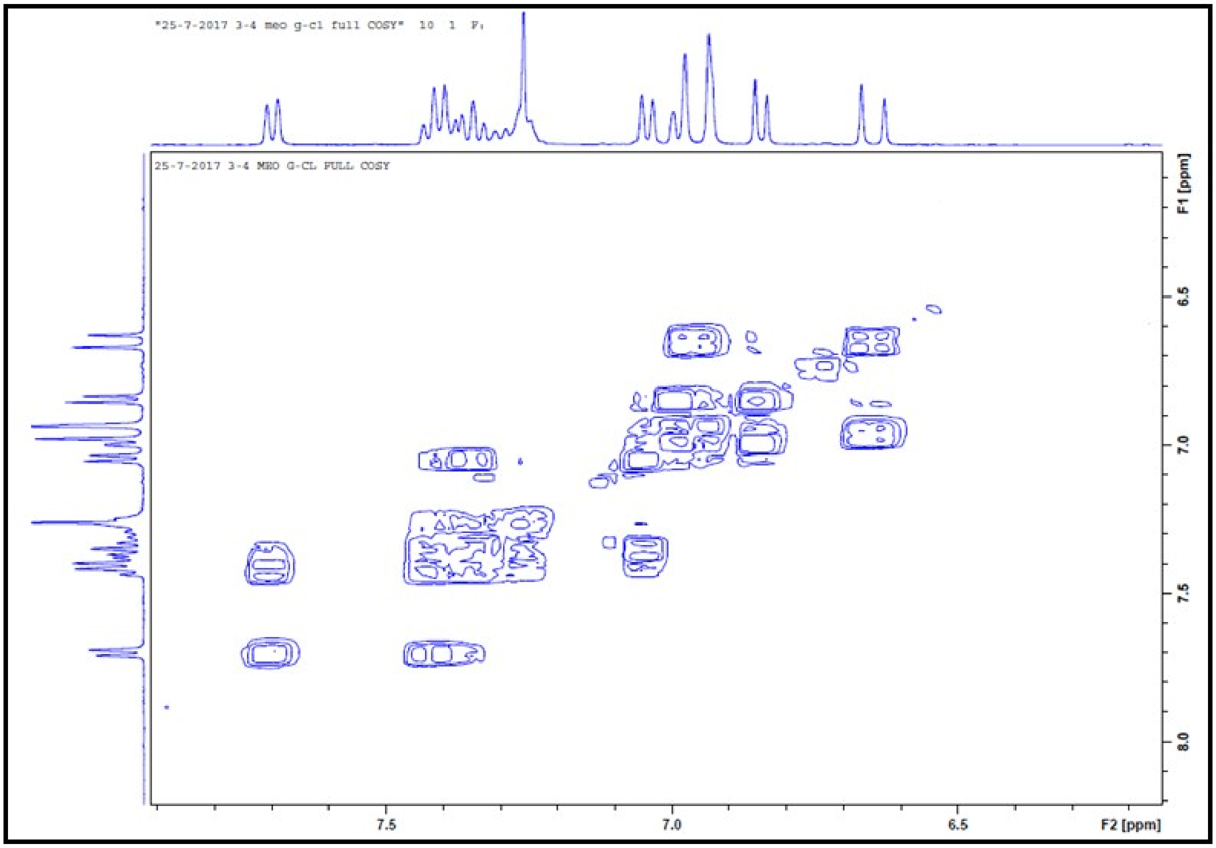

Figure S48: C COSY GPSW {^{1}H}($CDCl_3$, 400MHz) spectrum of compound bis(2((E)-(3,4-dimethoxystyryl)phenyl) glutaramide (EXPANSION).

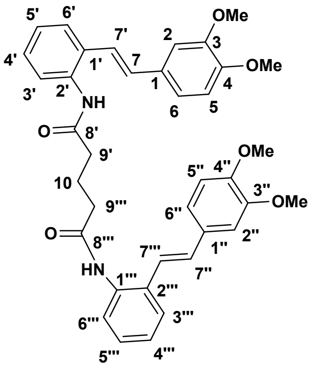

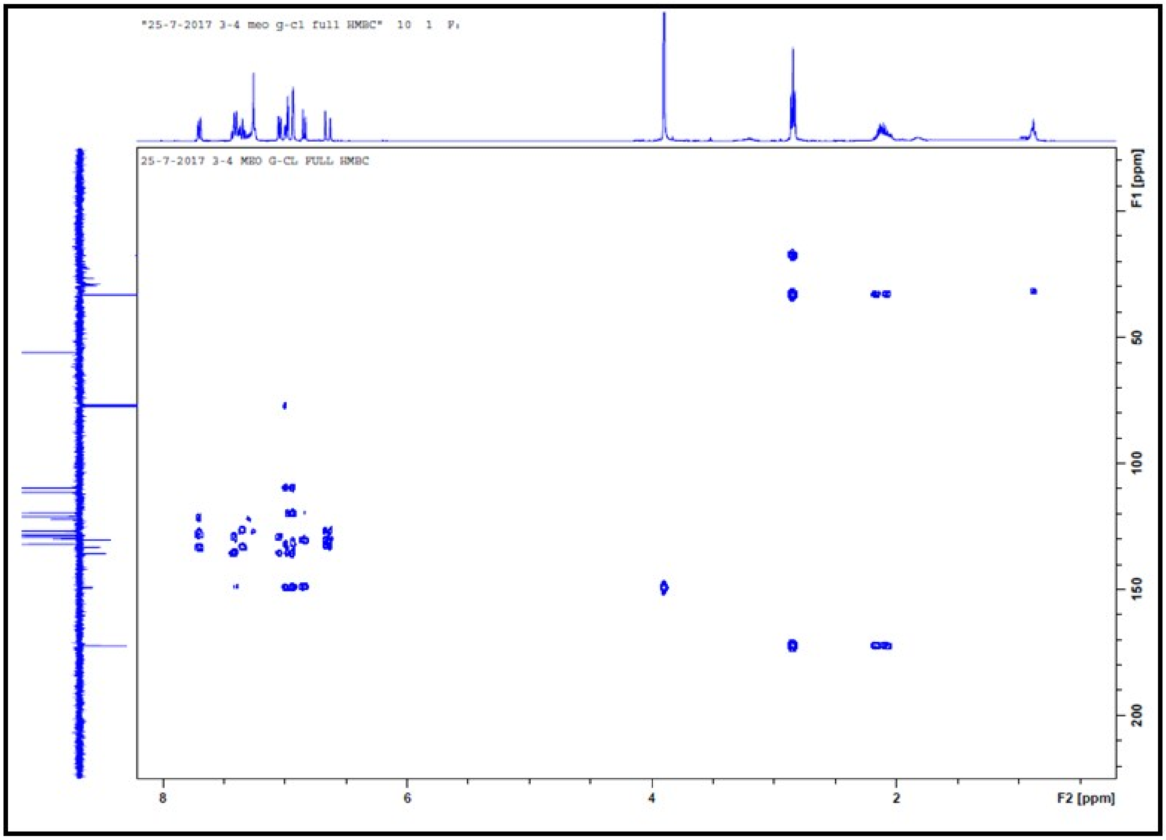

Figure S49: C HMBC GP {^{1}H- ^{13}C}($CDCl_3$, 400MHz) spectrum of compound bis(2((E)-(3,4-dimethoxystyryl)phenyl) glutaramide.

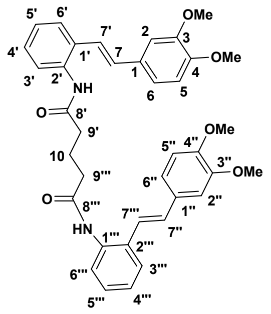

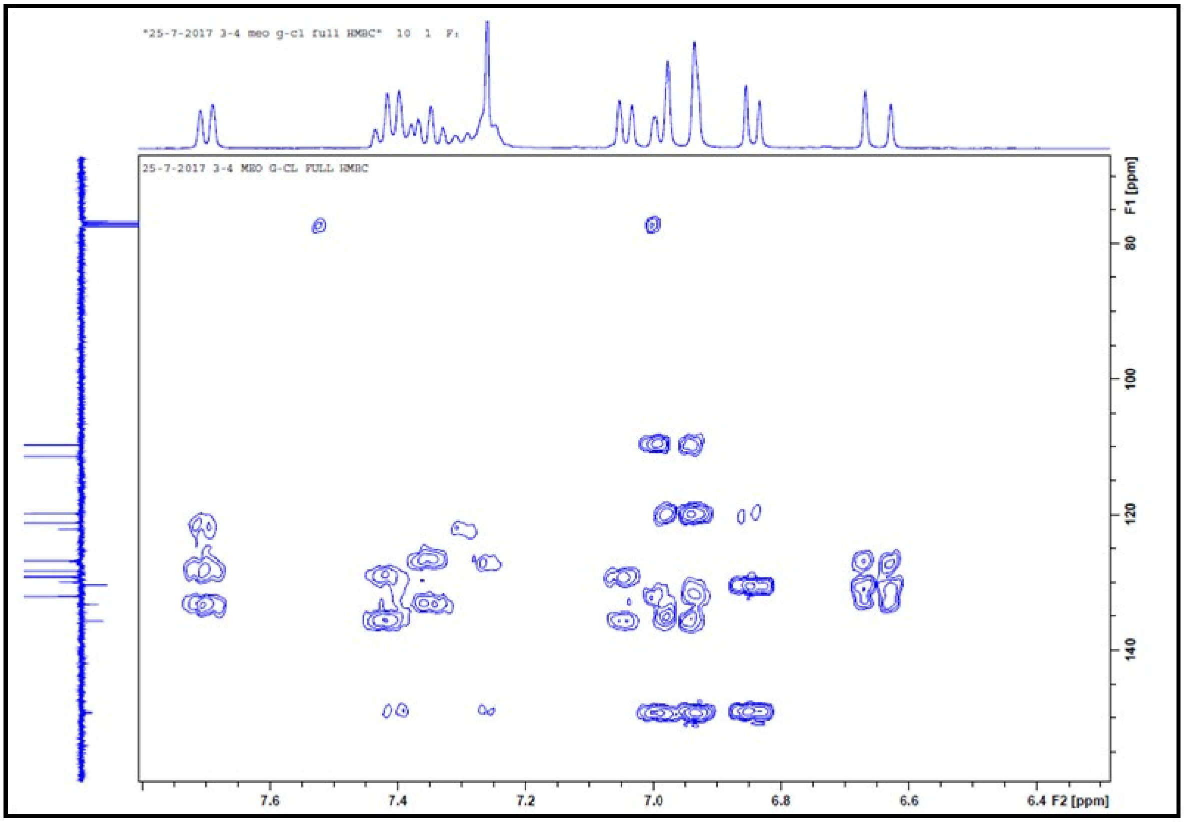

Figure S50: C HMBC GP {^{1}H- ^{13}C}($CDCl_3$, 400MHz) spectrum of compound bis(2((E)-(3,4-dimethoxystyryl)phenyl) glutaramide (EXPANSION).

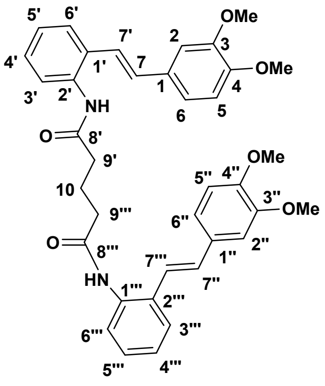

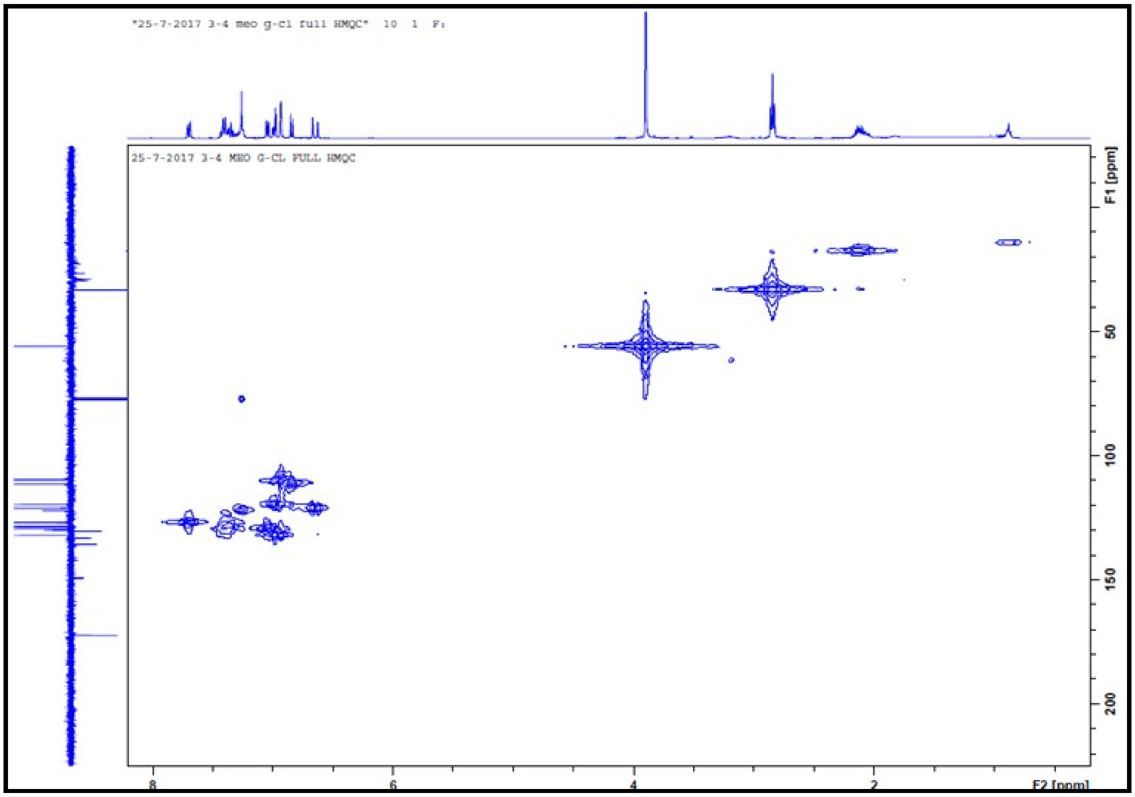

Figure S51: C HMQC GP {^{1}H-^{13}C}($CDCl_3$, 400MHz) spectrum of compound bis(2((E)-(3,4-dimethoxystyryl)phenyl) glutaramide.

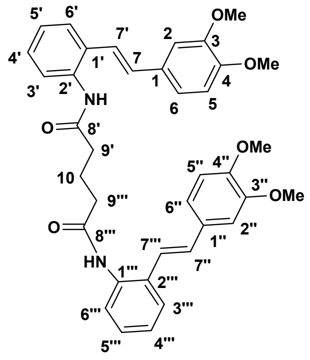

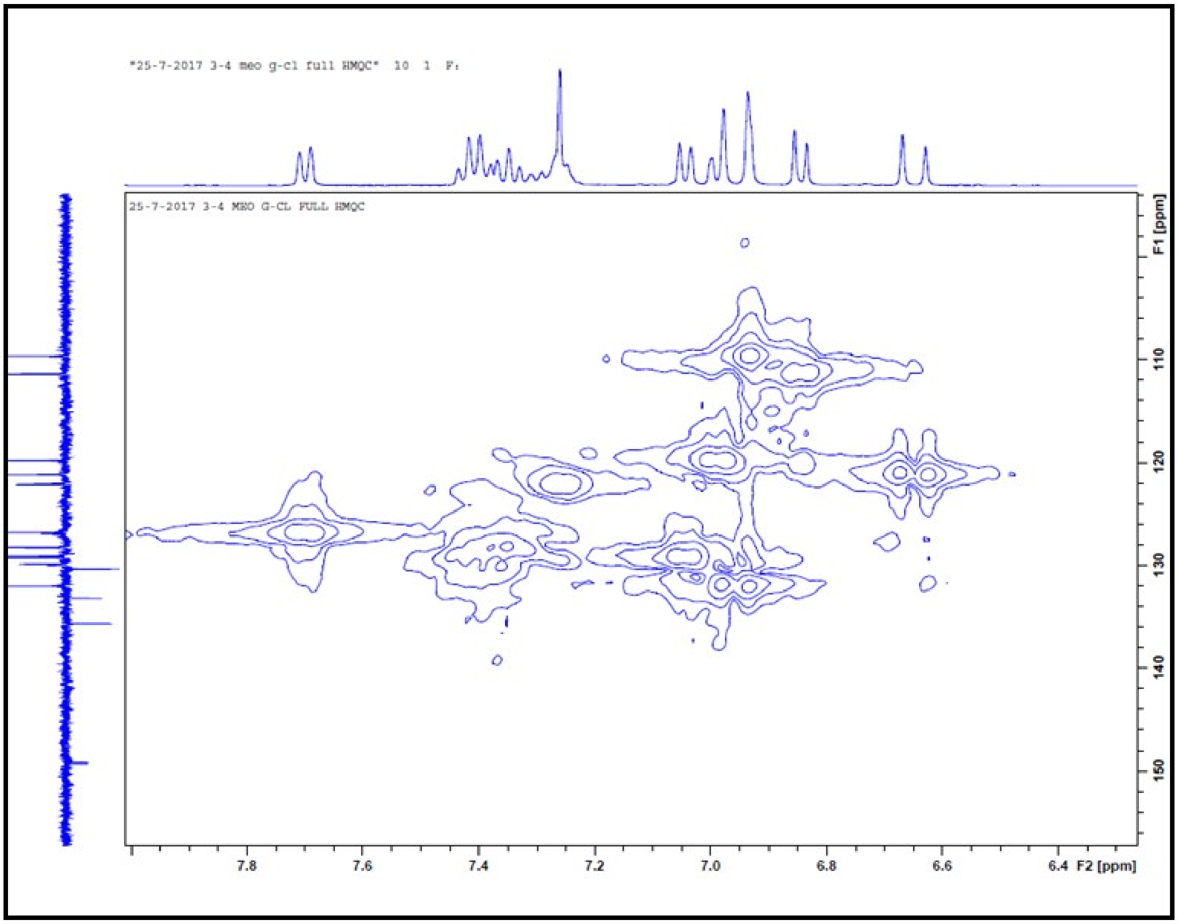

Figure S52: C HMQC GP {^{1}H-^{13}C}(CDCl$_3$, 400MHz) spectrum of compound bis(2((E)-(3,4-dimethoxystyryl)phenyl) glutaramide (EXPANSION).

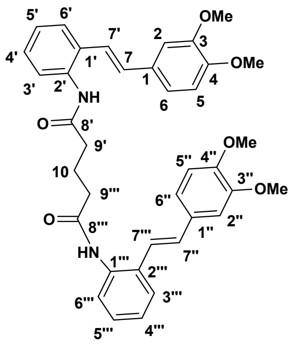

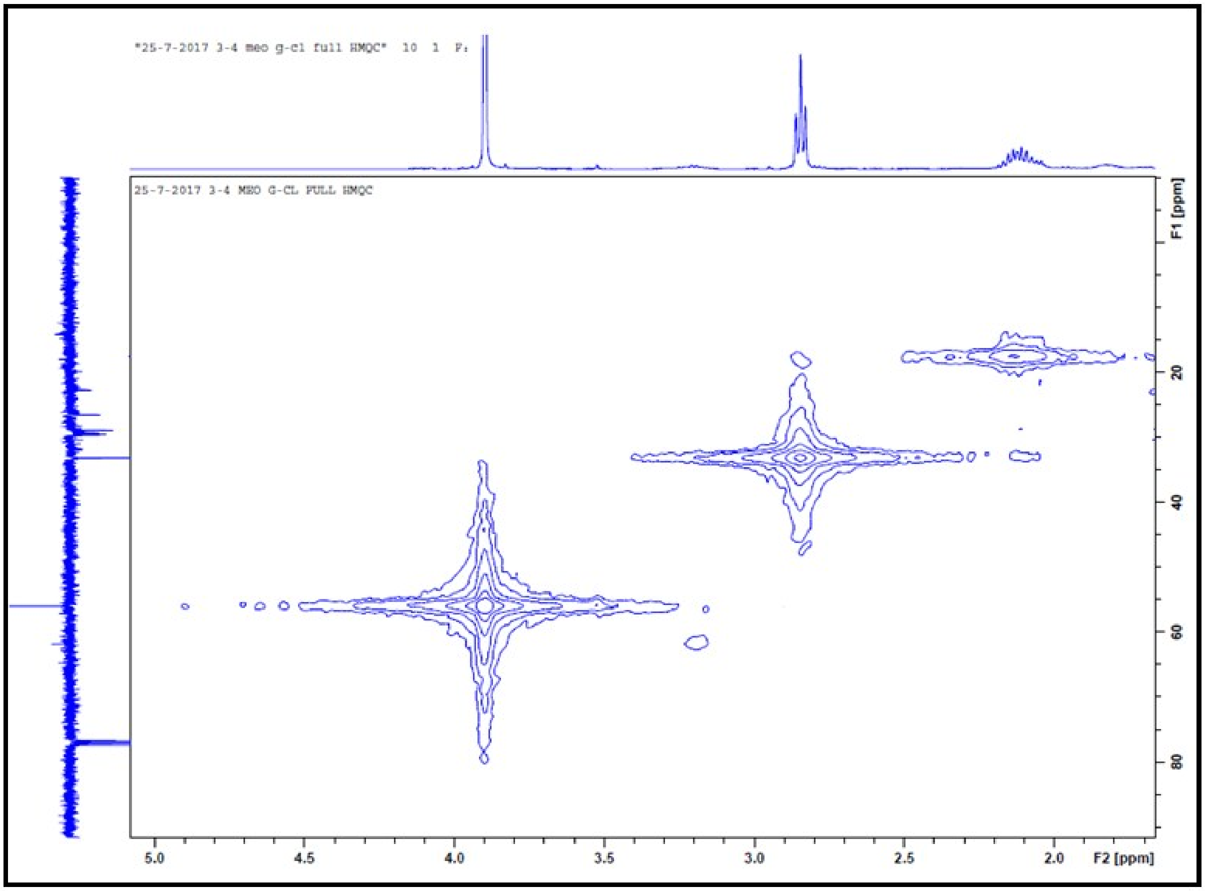

Figure S53: C HMQC GP {^{1}H-^{13}C}($CDCl_3$, 400MHz) spectrum of compound bis(2((E)-(3,4-dimethoxystyryl)phenyl) glutaramide (EXPANSION).

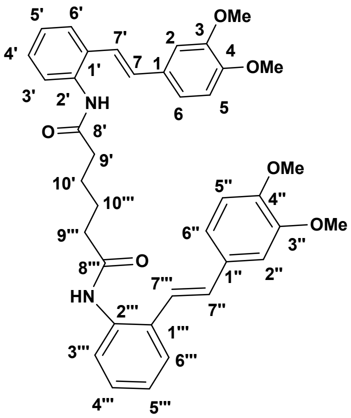

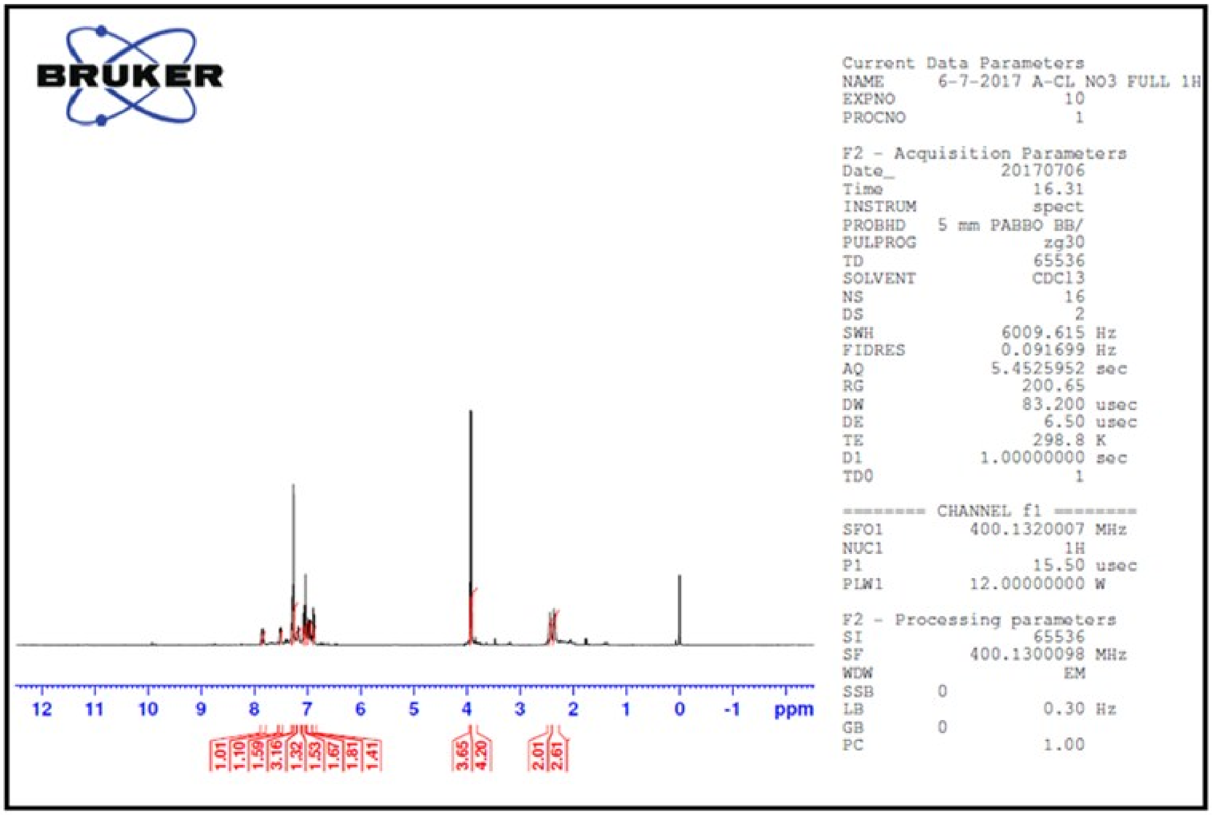

Figure S54:[1]**H NMR (CDCl$_3$, 400MHz) spectrum of compound bis(2((E)-(3,4-dimethoxystyryl)phenyl)adipamide.**

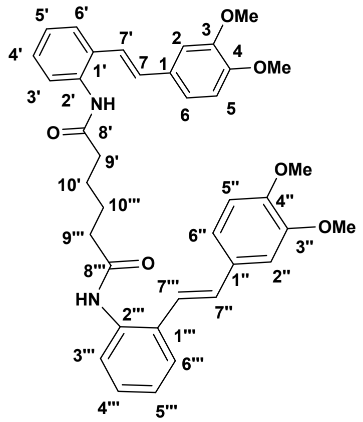

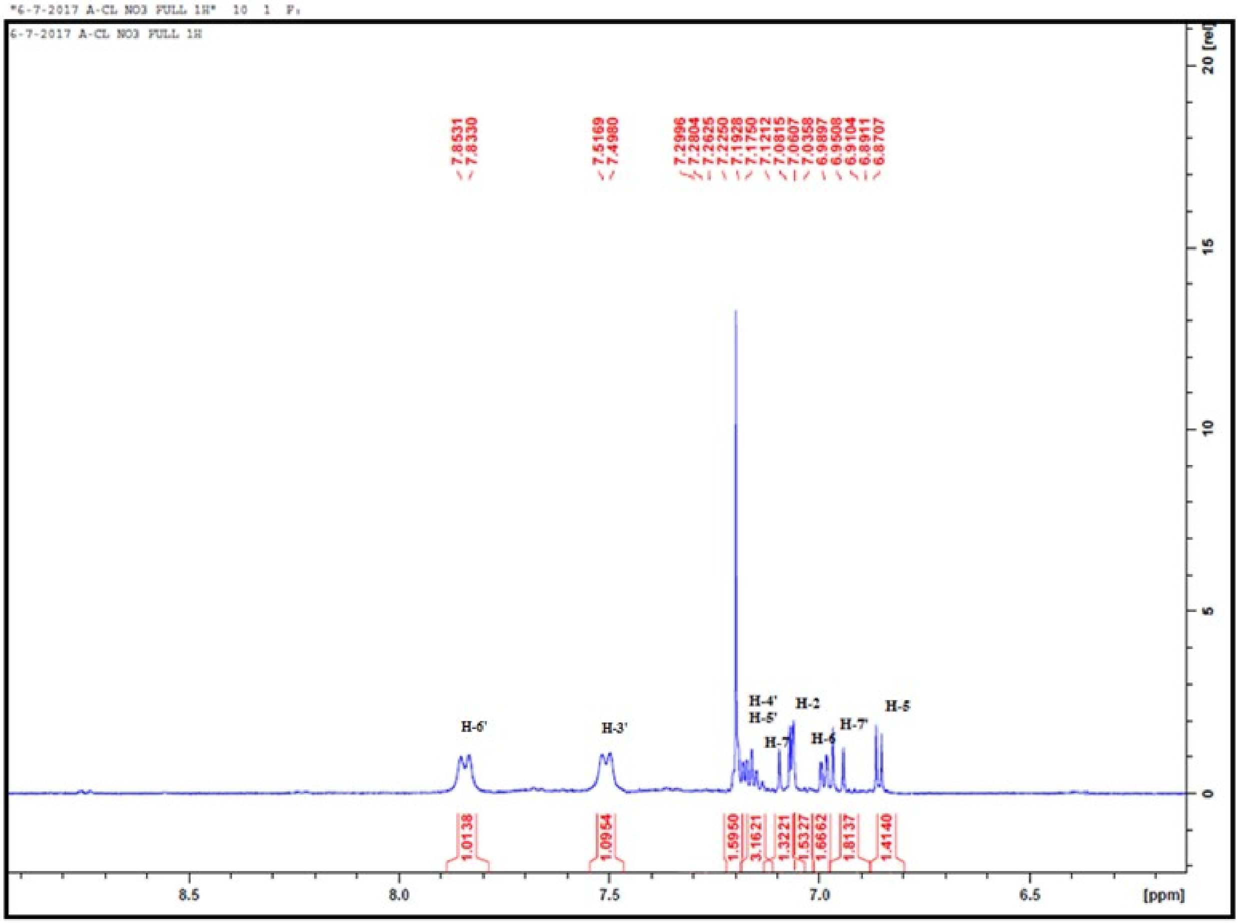

Figure S55: ^{1}H NMR ($CDCl_3$, 400MHz) spectrum of compound bis(2((E)-(3,4-dimethoxystyryl)phenyl)adipamide

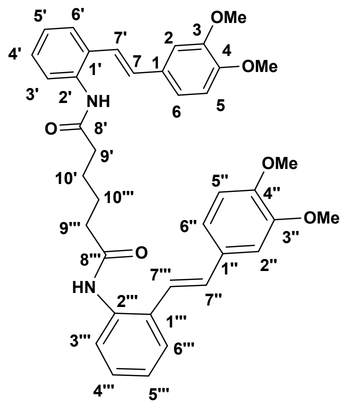

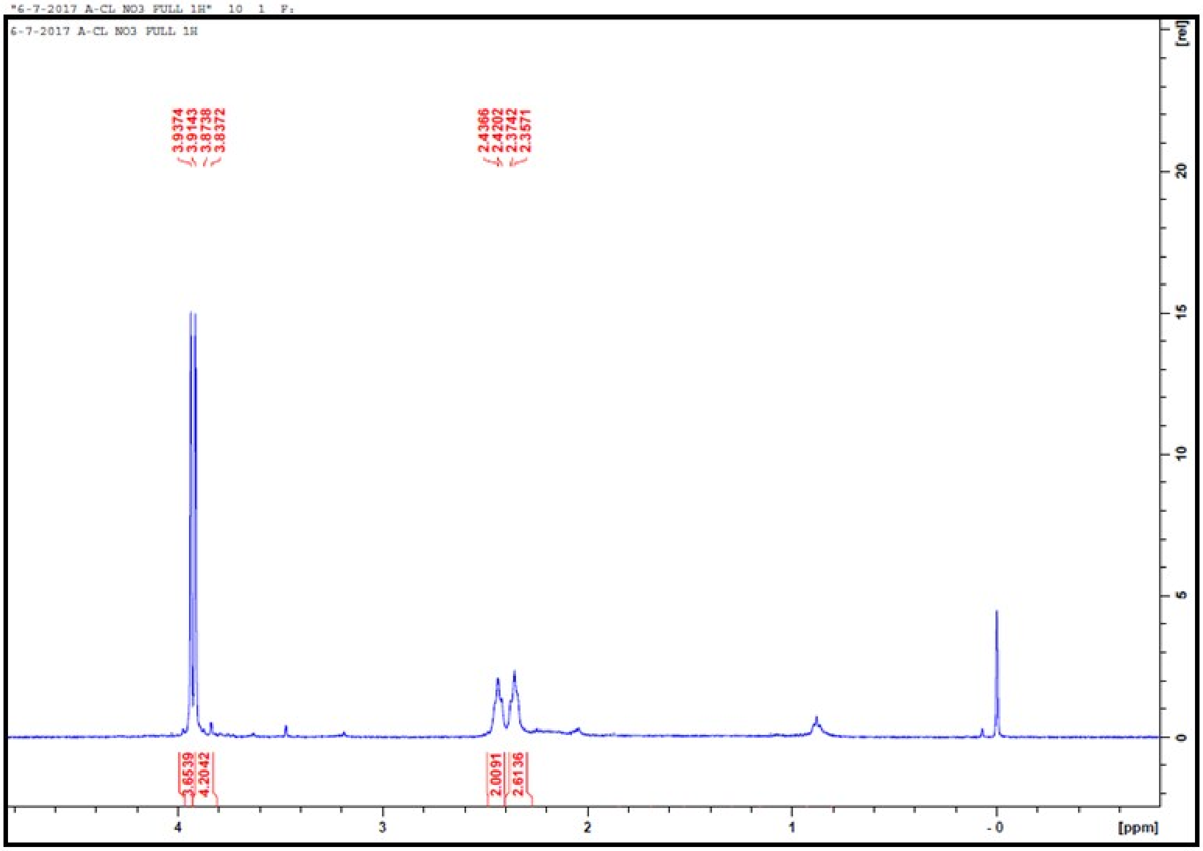

Figure S56: ^{1}H NMR ($CDCl_3$, 400MHz) spectrum of compound bis(2((E)-(3,4-dimethoxystyryl)phenyl)adipamide (EXPANSION).

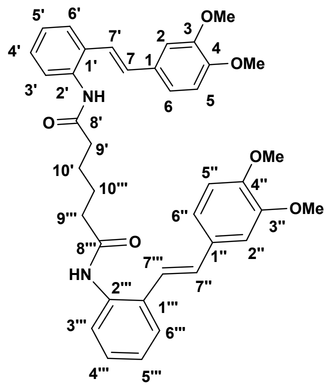

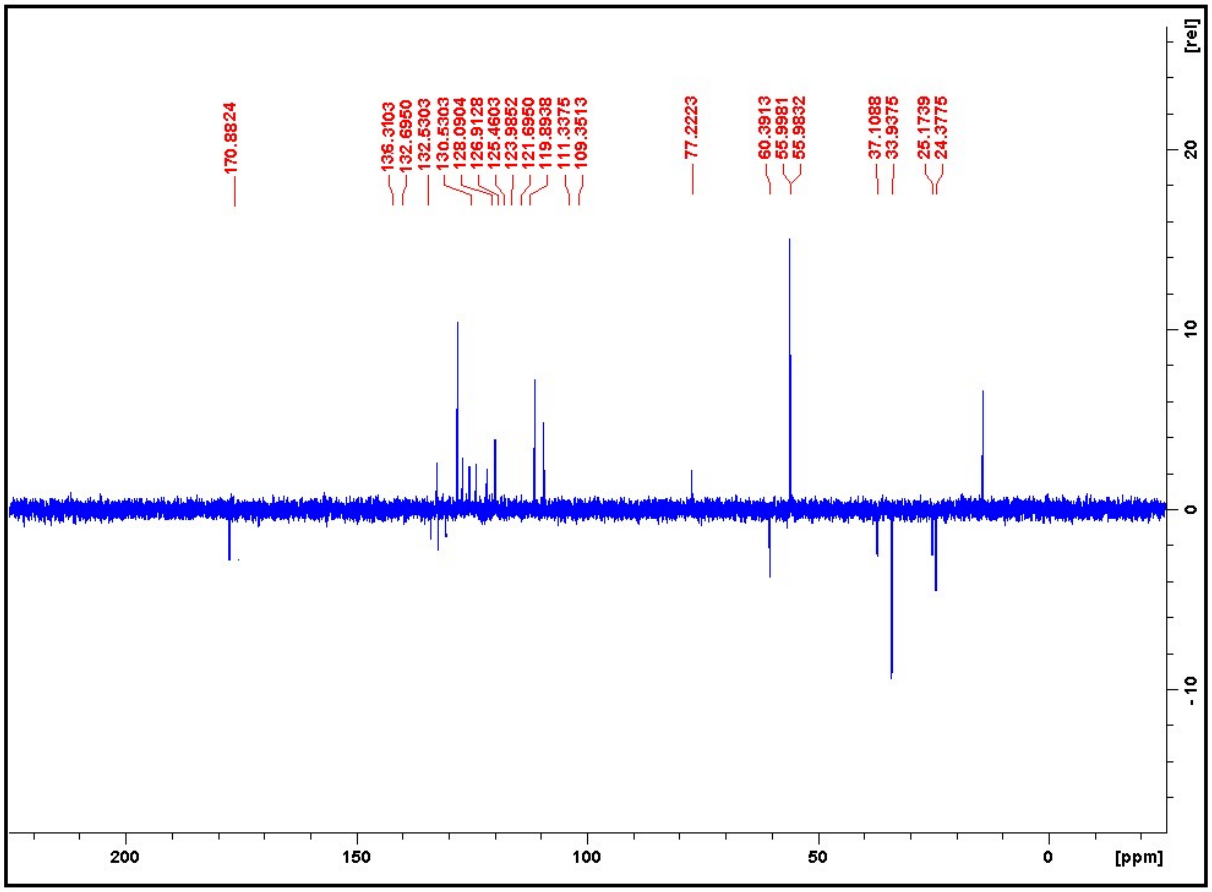

Figure S57: ^{13}C DEPT135,CH_3/CH po {^{1}H}($CDCl_3$, 400MHz) spectrum of compound bis(2((E)-(3,4-dimethoxystyryl)phenyl)adipamide.

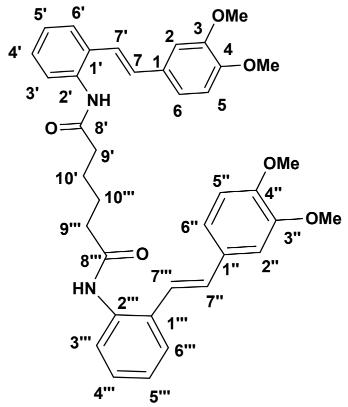

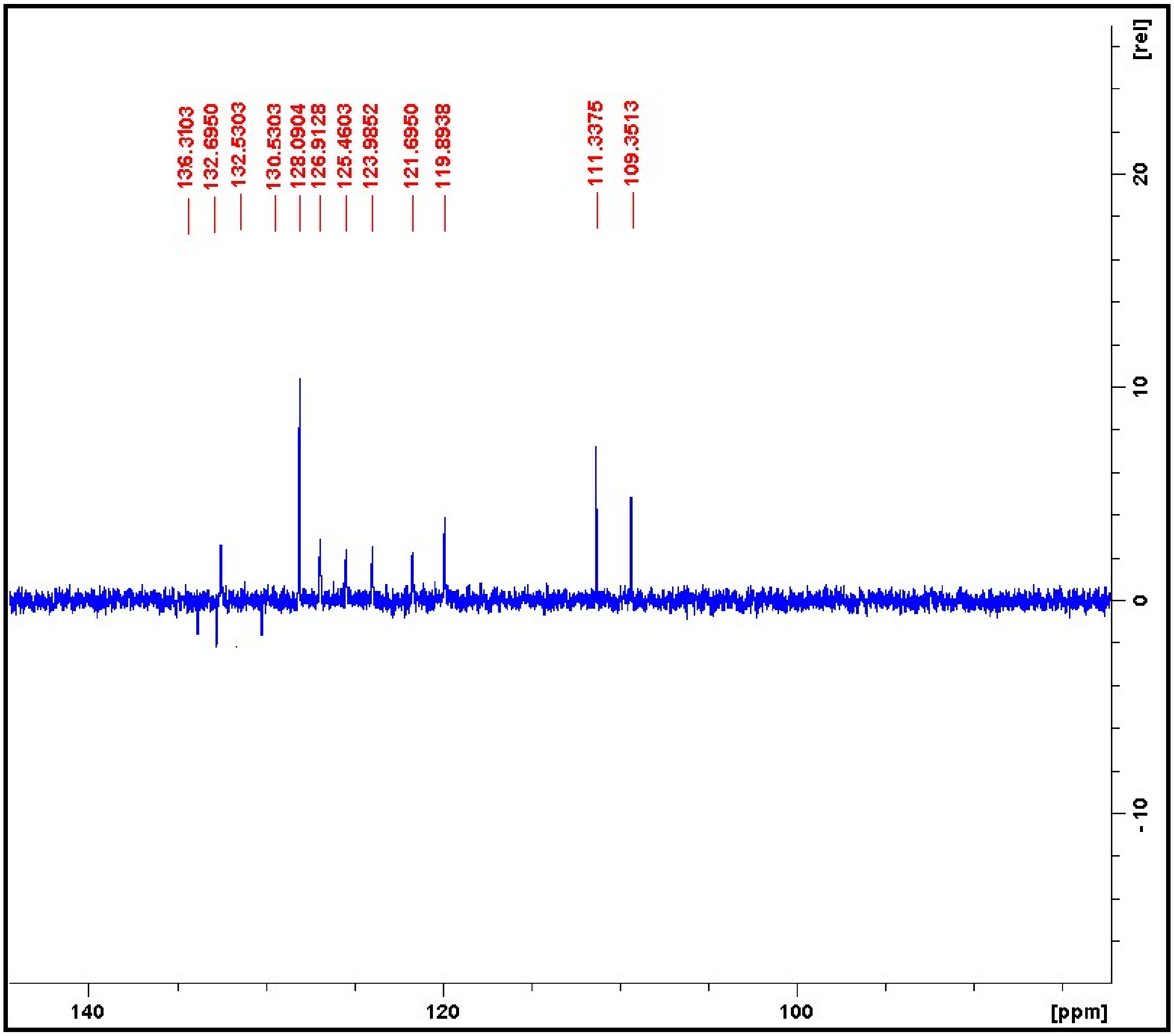

Figure S58: ^{13}C DEPT135,CH_3/CH po {^{1}H}($CDCl_3$, 400MHz) spectrum of compound bis(2((E)-(3,4-dimethoxystyryl)phenyl)adipamide (EXPANSION).

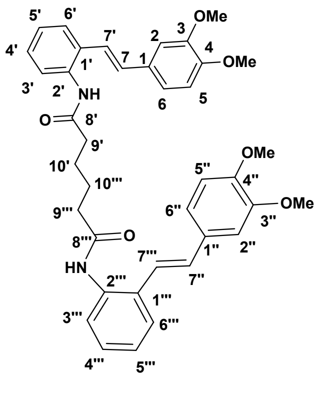

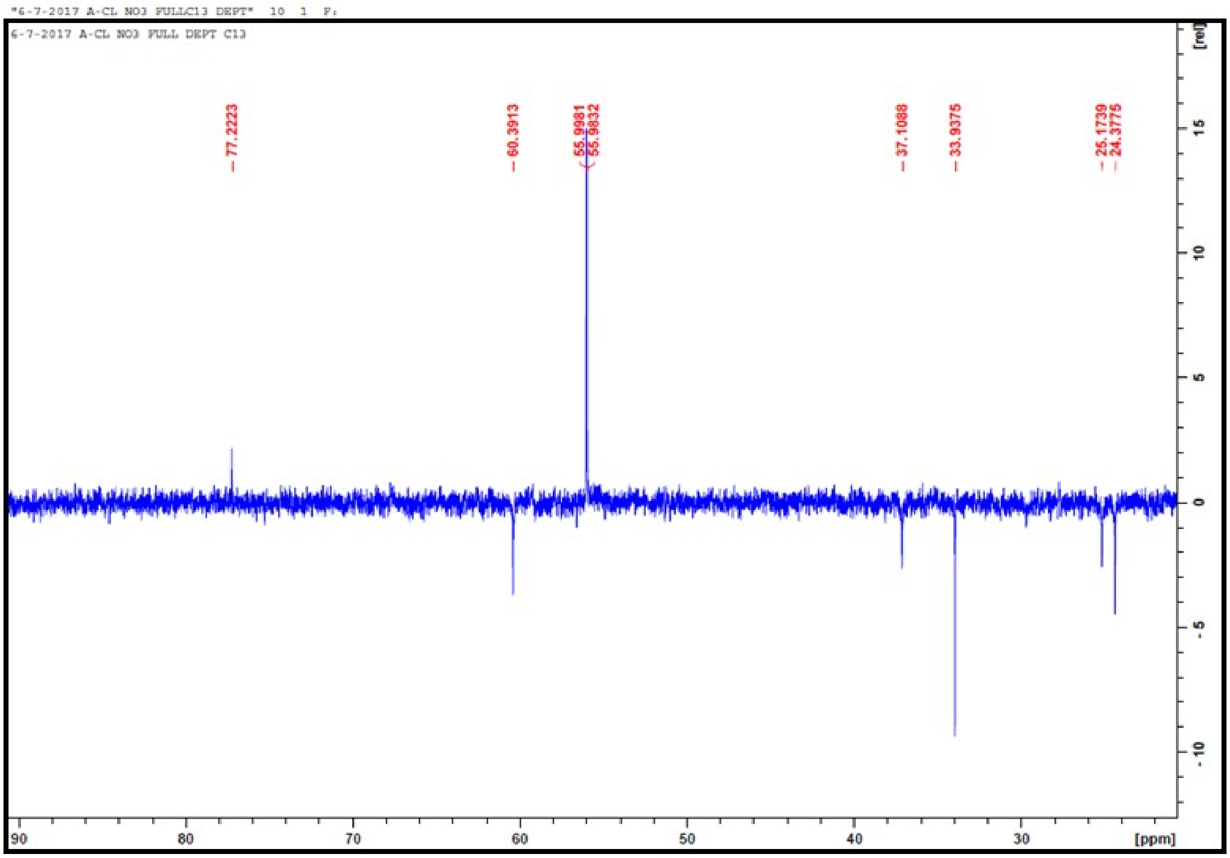

Figure S59: ^{13}C DEPT135,CH_3/CH po {^{1}H}($CDCl_3$, 400MHz) spectrum of compound bis(2((E)-(3,4-dimethoxystyryl)phenyl)adipamide (EXPANSION).

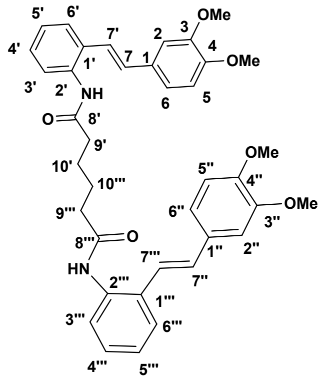

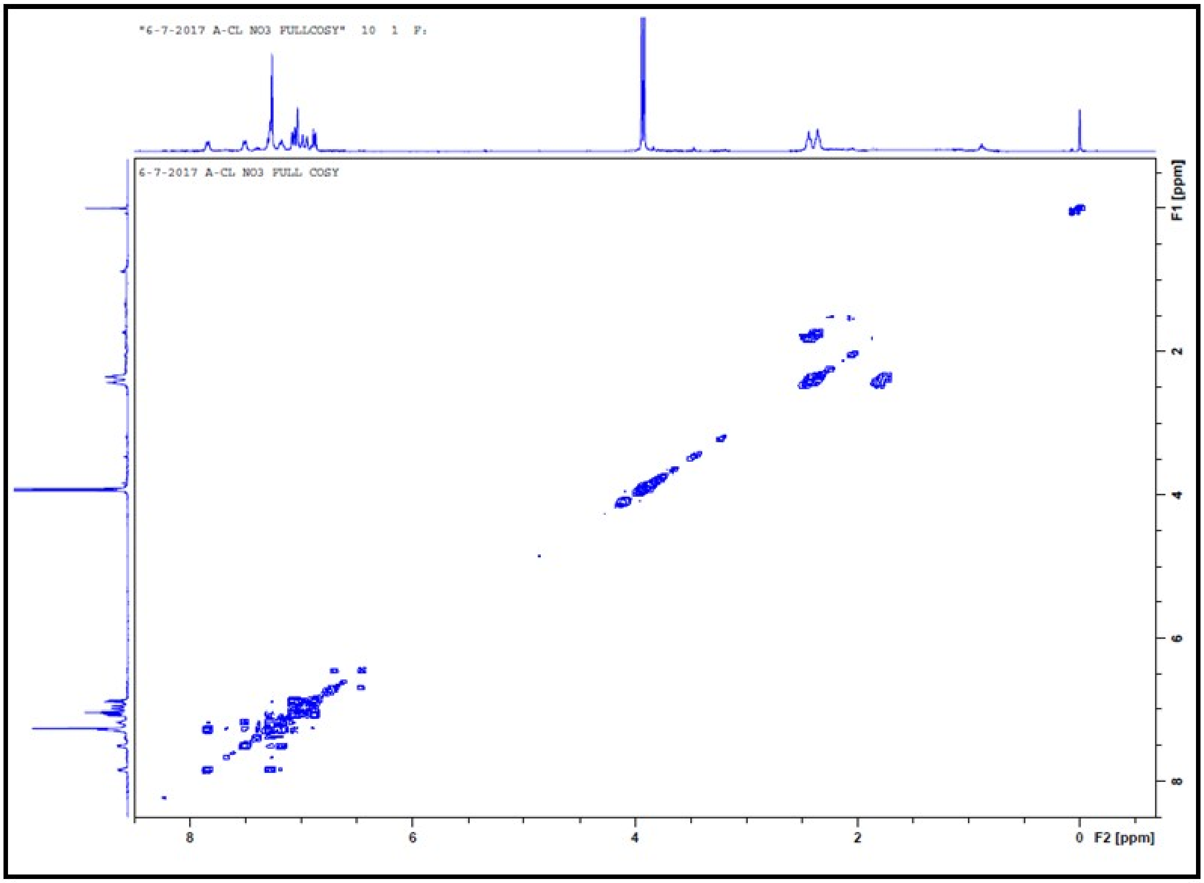

Figure S60: C COSY GPSW $\{^1H\}$($CDCl_3$, 400MHz) spectrum of compound bis(2((E)-(3,4-dimethoxystyryl)phenyl)adipamide.

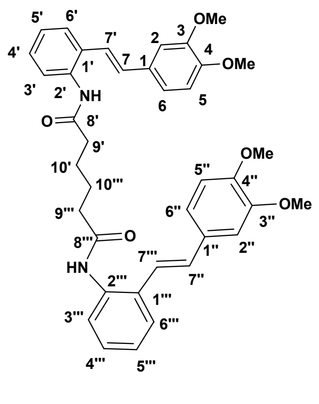

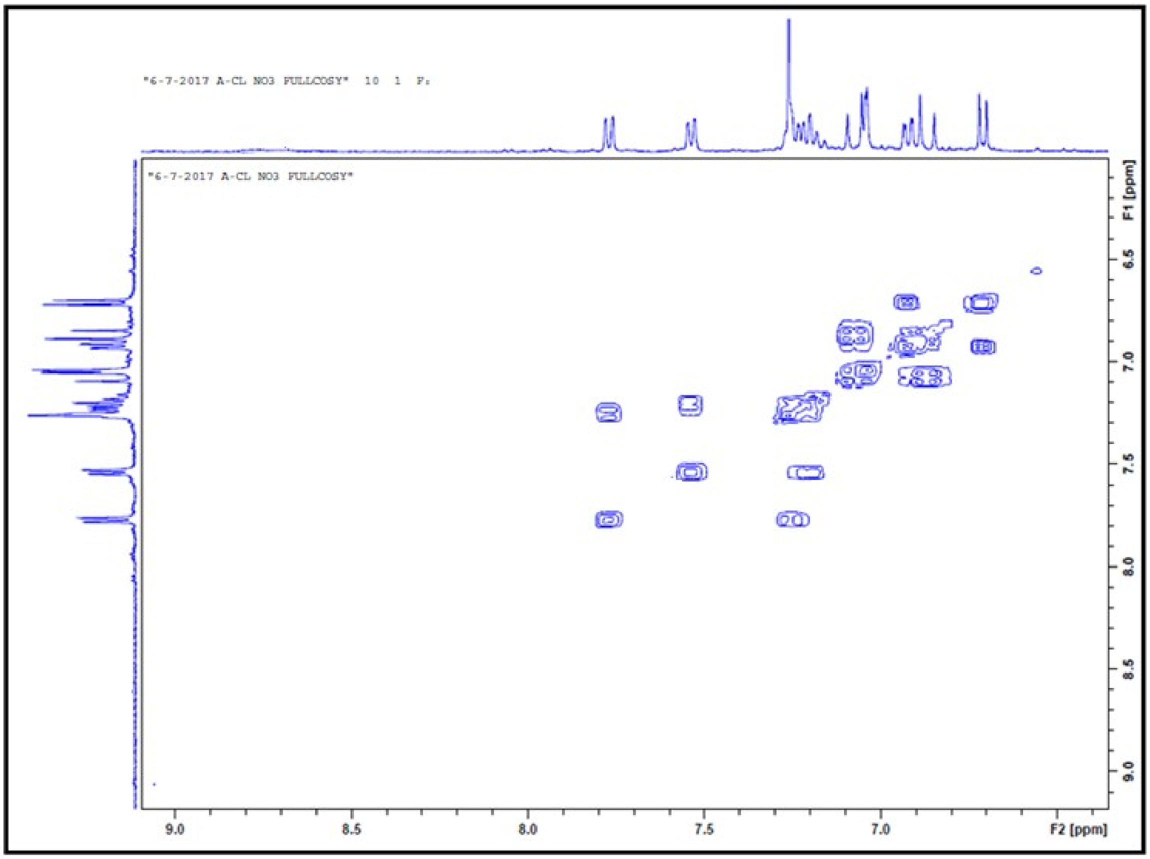

Figure S61: C COSY GPSW {^{1}H}($CDCl_3$, 400MHz) spectrum of compound bis(2((E)-(3,4-dimethoxystyryl)phenyl)adipamide (EXPANSION).

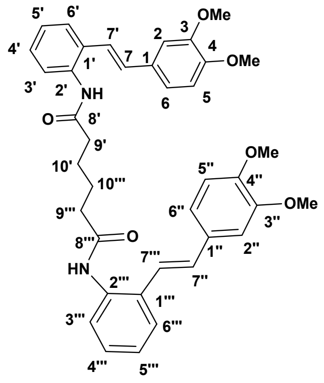

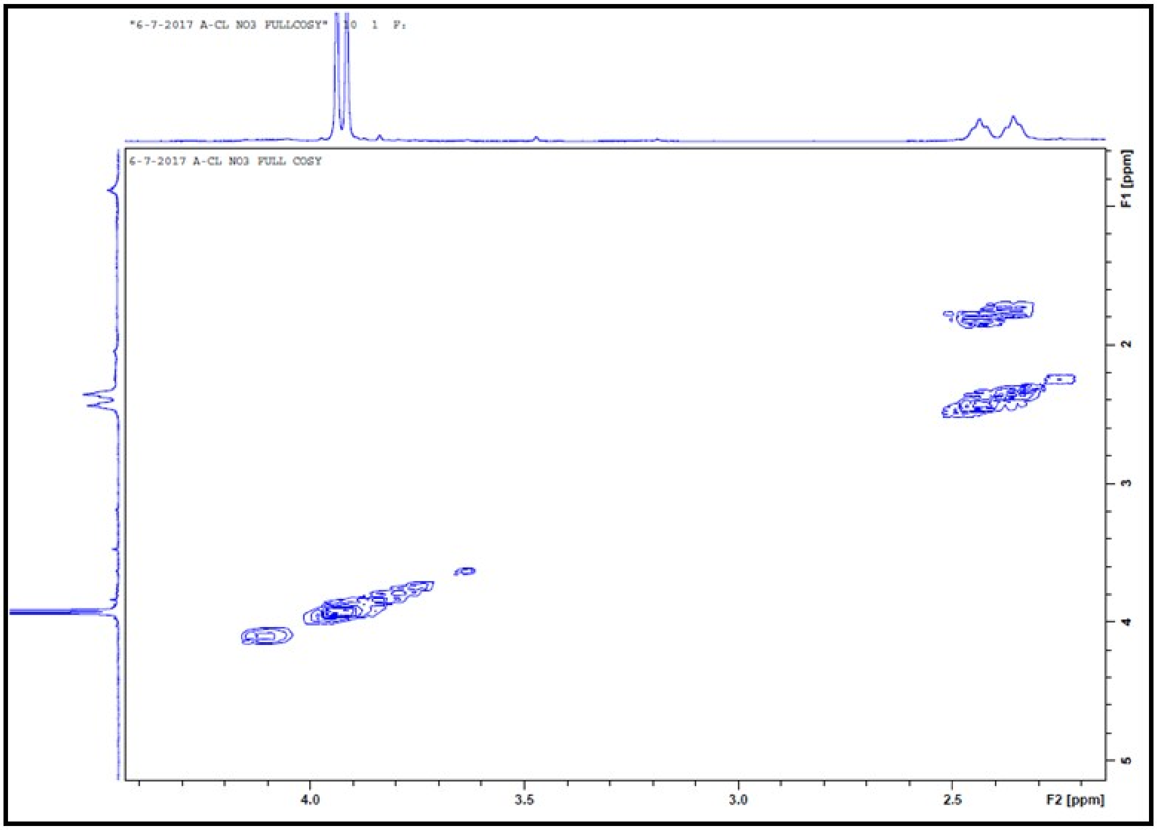

Figure S62: C COSY GPSW {^{1}H}($CDCl_3$, 400MHz) spectrum of compound bis(2((E)-(3,4-dimethoxystyryl)phenyl)adipamide (EXPANSION).

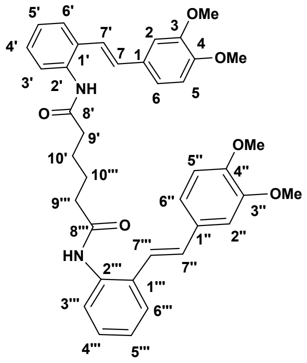

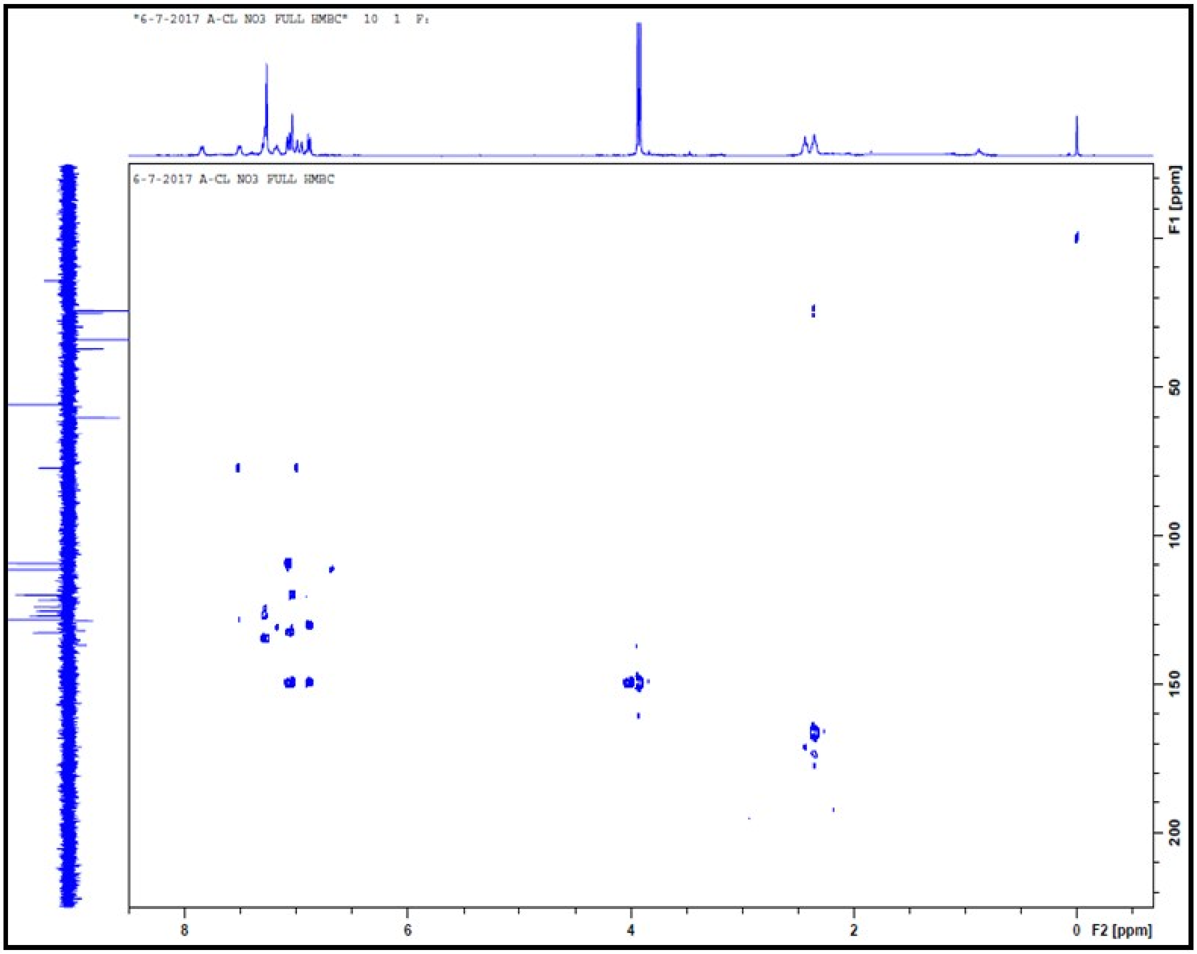

Figure S63: C HMBC GP {^{1}H- ^{13}C}($CDCl_3$, 400MHz) spectrum of compound bis(2((E)-(3,4-dimethoxystyryl)phenyl)adipamide.

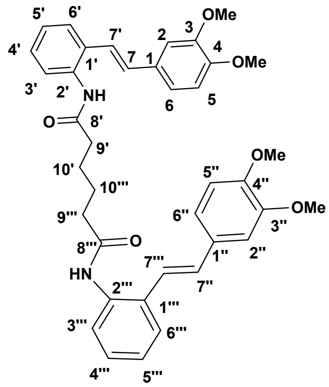

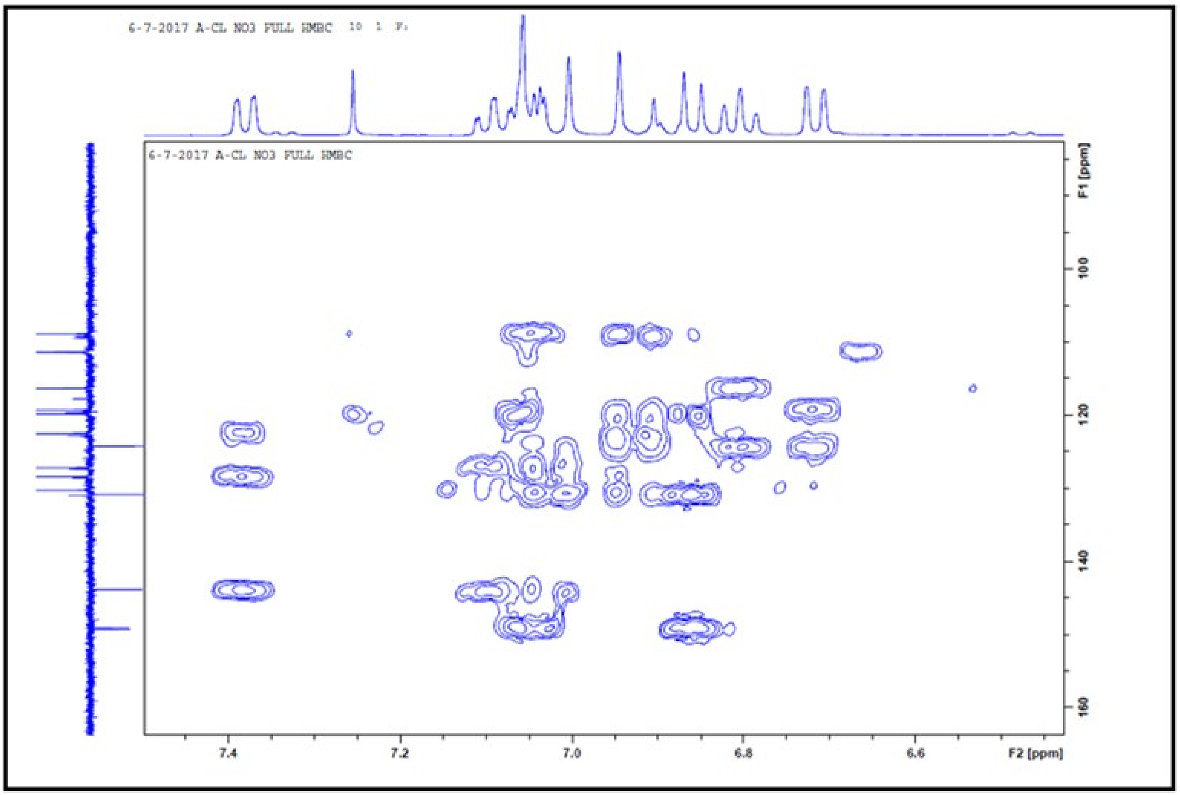

Figure S64: C HMBC GP {^{1}H- ^{13}C}($CDCl_3$, 400MHz) spectrum of compound bis(2((E)-(3,4-dimethoxystyryl)phenyl)adipamide (EXPANSION).

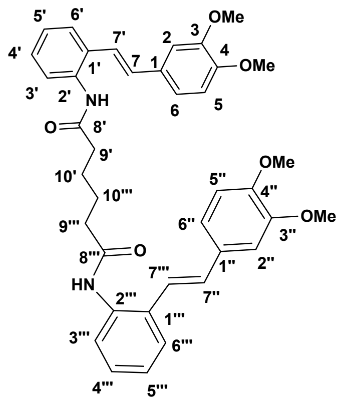

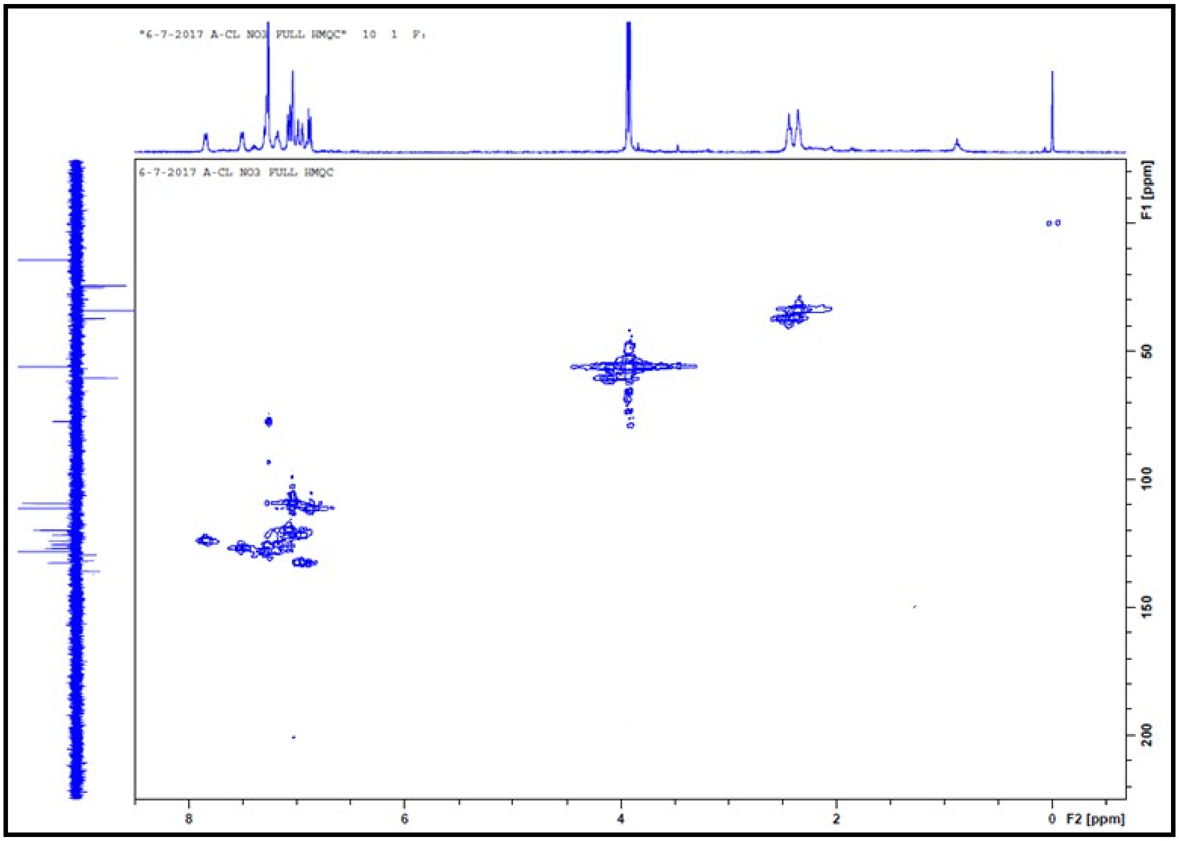

Figure S65: C HMQC GP {^{1}H-^{13}C}($CDCl_3$, 400MHz) spectrum of compound bis(2((E)-(3,4-dimethoxystyryl)phenyl)adipamide.

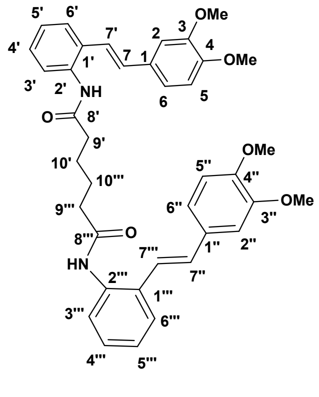

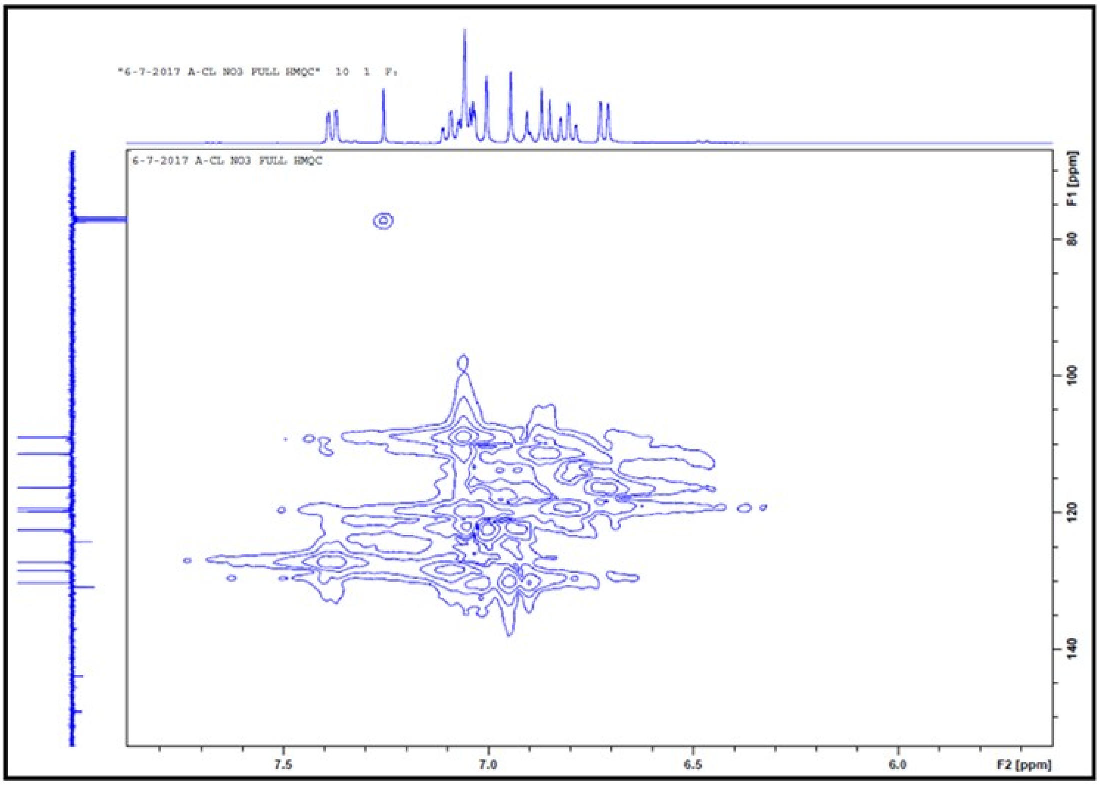

Figure S66: C HMQC GP {^{1}H-^{13}C}($CDCl_3$, 400MHz) spectrum of compound bis(2((E)-(3,4-dimethoxystyryl)phenyl)adipamide (EXPANSION).

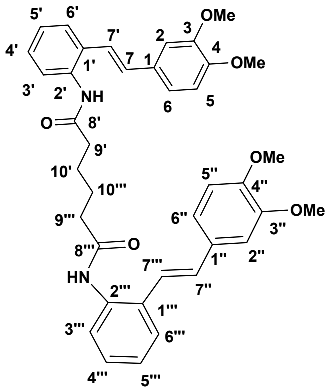

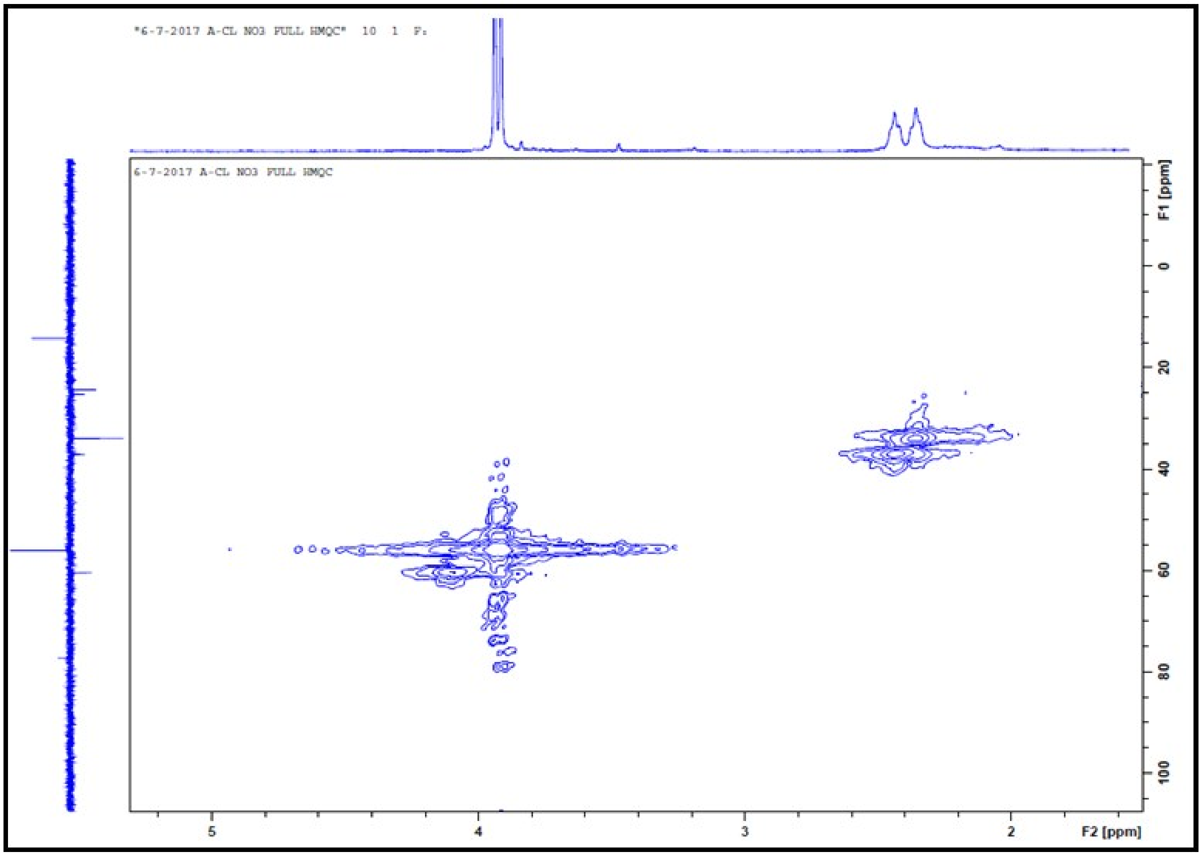

Figure S67: C HMQC GP {^{1}H-^{13}C}(CDCl$_3$, 400MHz) spectrum of compound bis(2((E)-(3,4-dimethoxystyryl)phenyl)adipamide (EXPANSION).

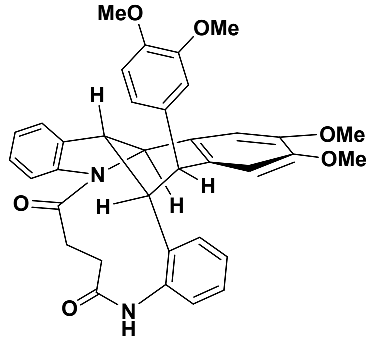

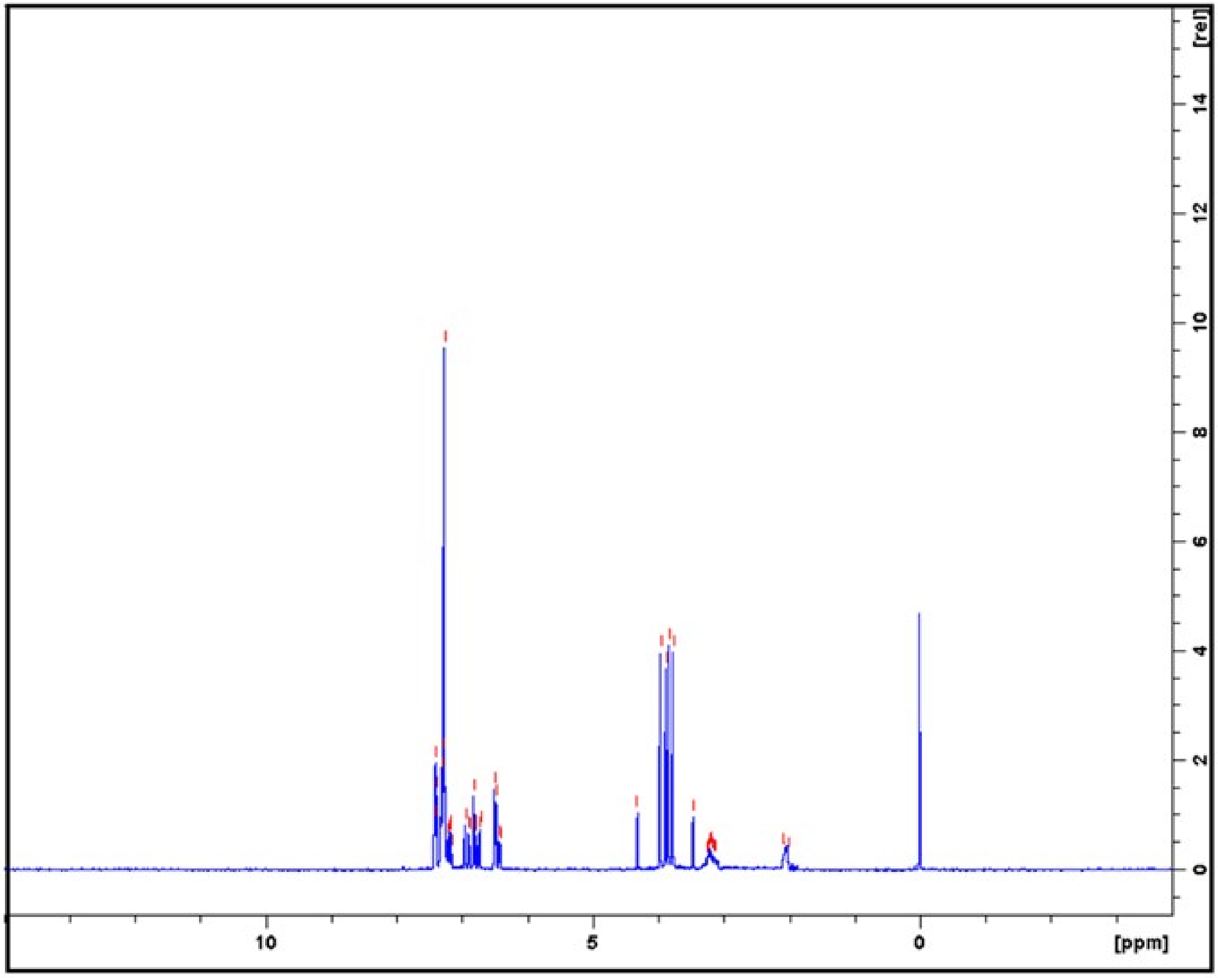

Figure S68: ^{1}H NMR ($CDCl_3$, 400MHz) spectrum of compound (73) Scheme 17.

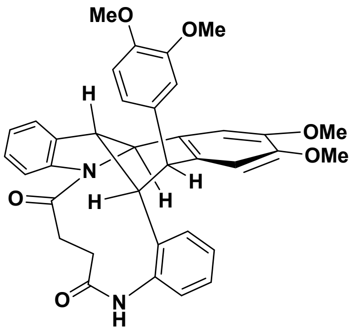

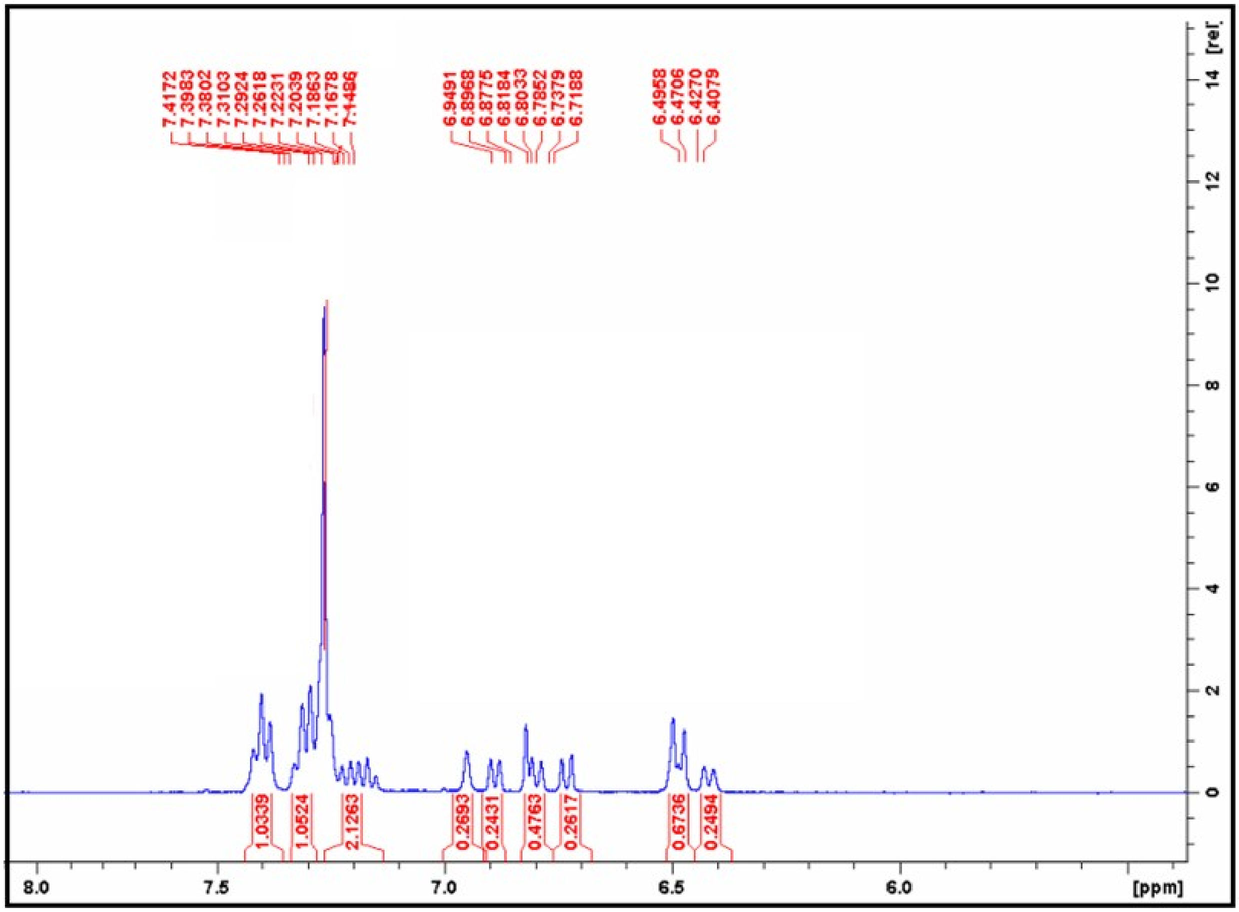

Figure S69: ^{1}H NMR ($CDCl_3$, 400MHz) spectrum of compound (73) Scheme 17 (EXPANSION).

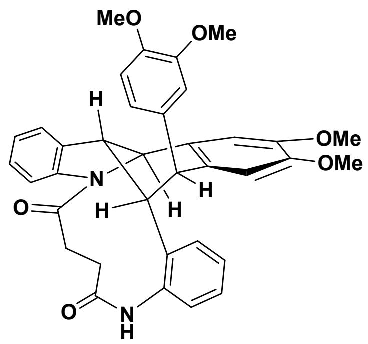

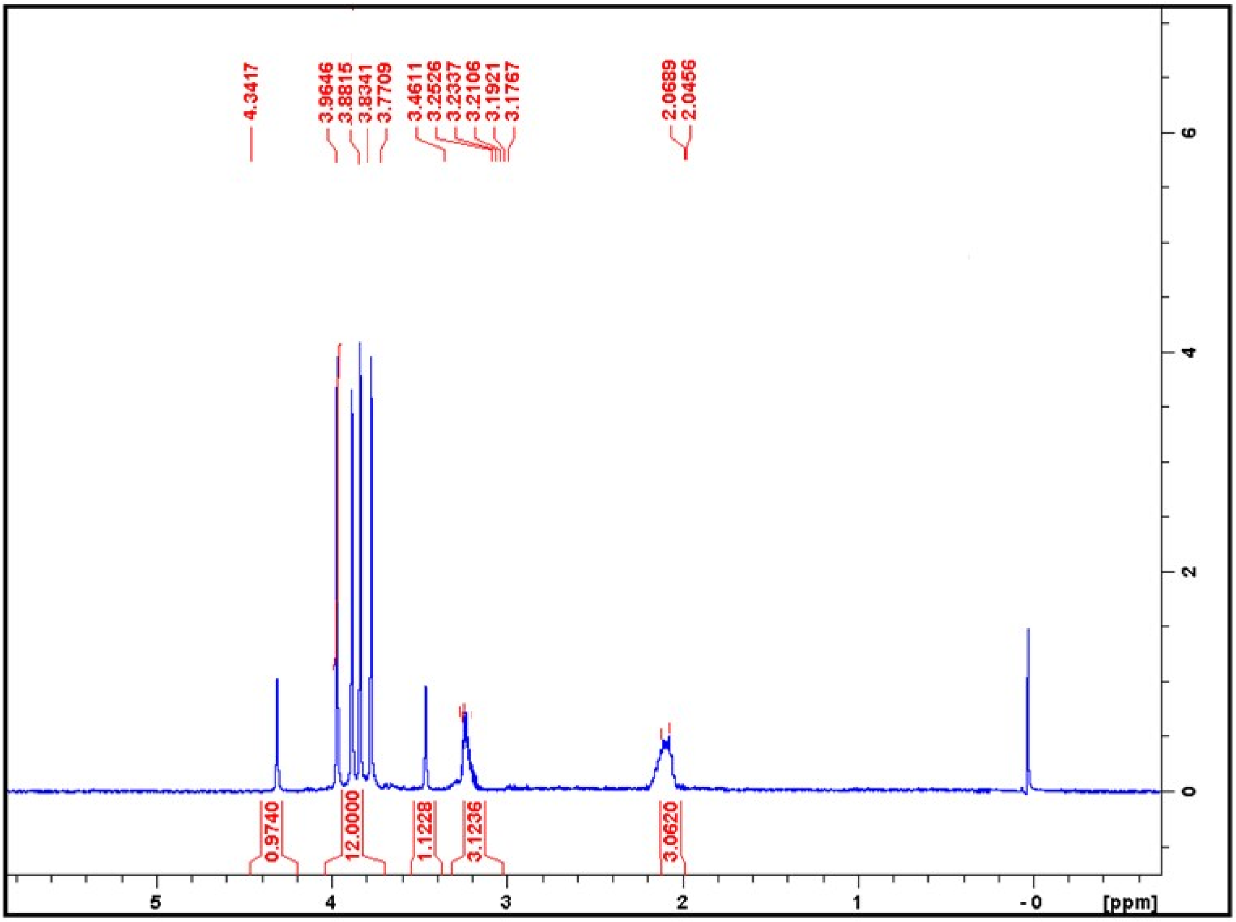

Figure S70: ^{1}H NMR ($CDCl_3$, 400MHz) spectrum of compound (73) Scheme 17 (EXPANSION).

1 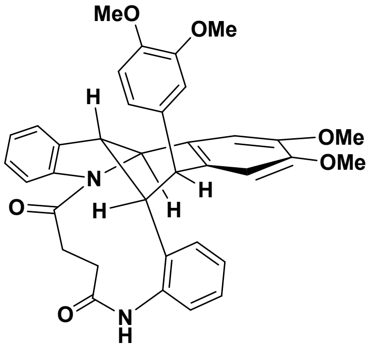

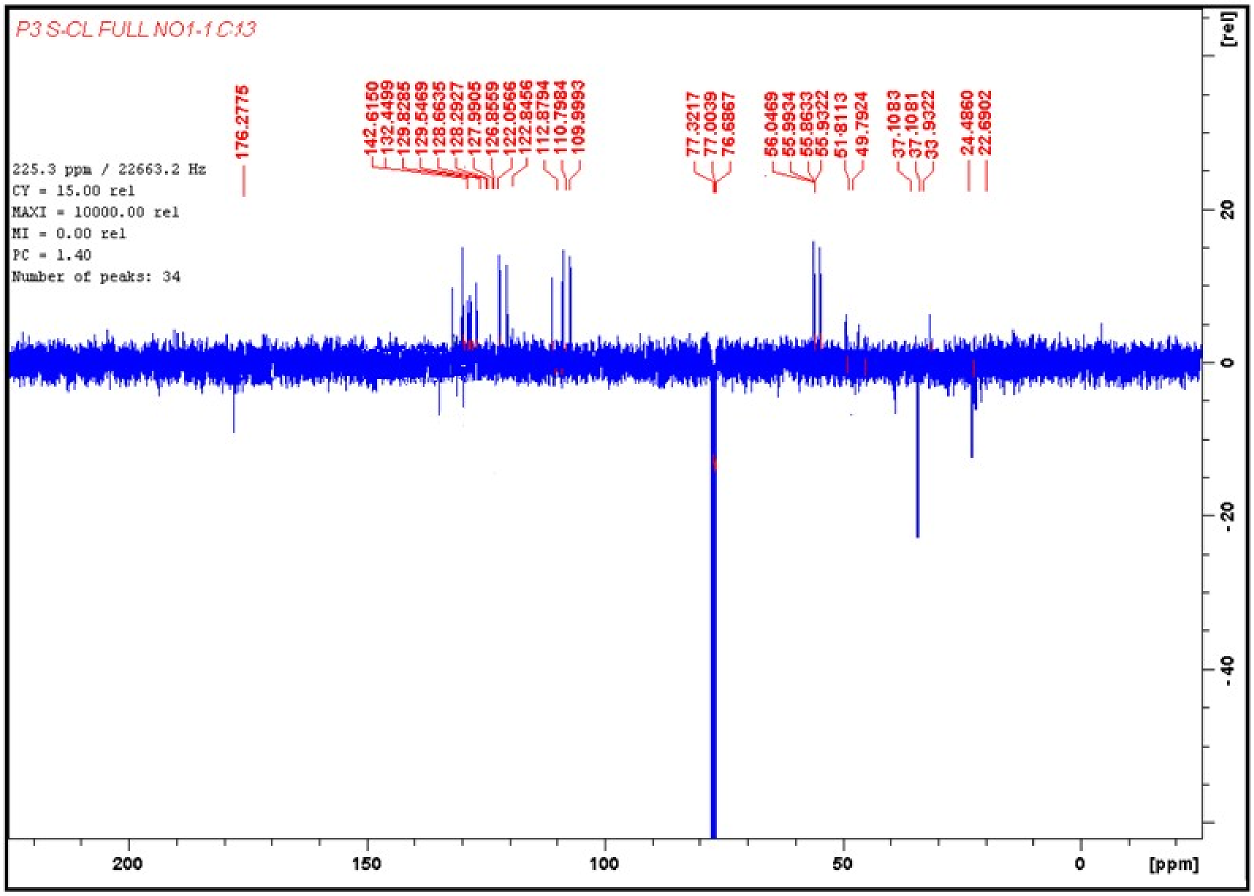

2
3 **Figure S71:^{13}C DEPT135,CH$_3$/CH po {^{1}H}(CDCl$_3$, 400MHz) spectrum of compound compund (73) Scheme**

4 **17.**

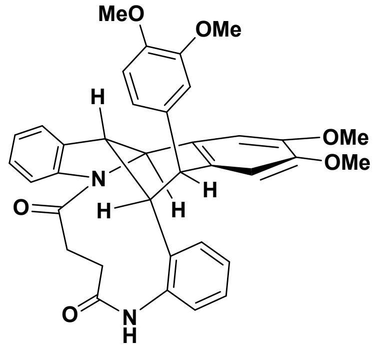

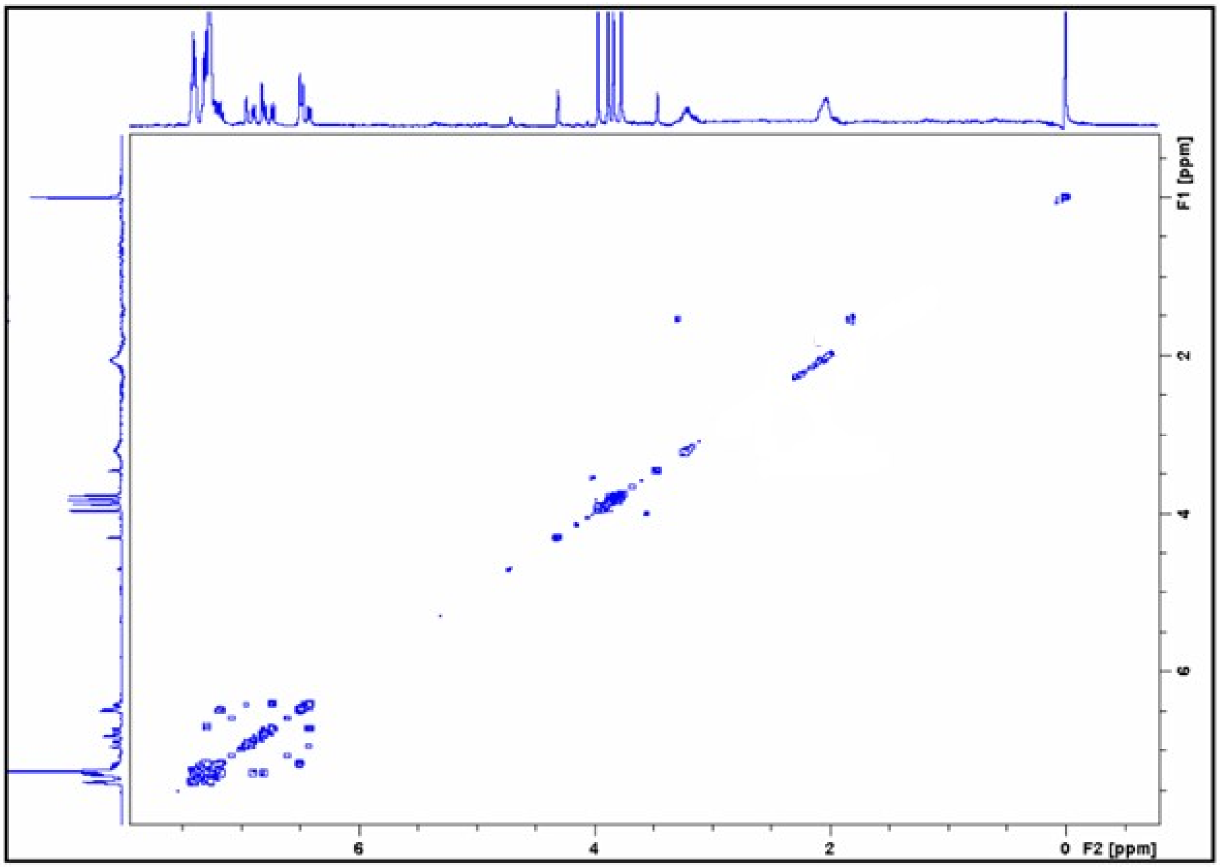

Figure S72: C COSY GPSW {^{1}H}($CDCl_3$, 400MHz) spectrum of compound compund (73) Scheme 17.

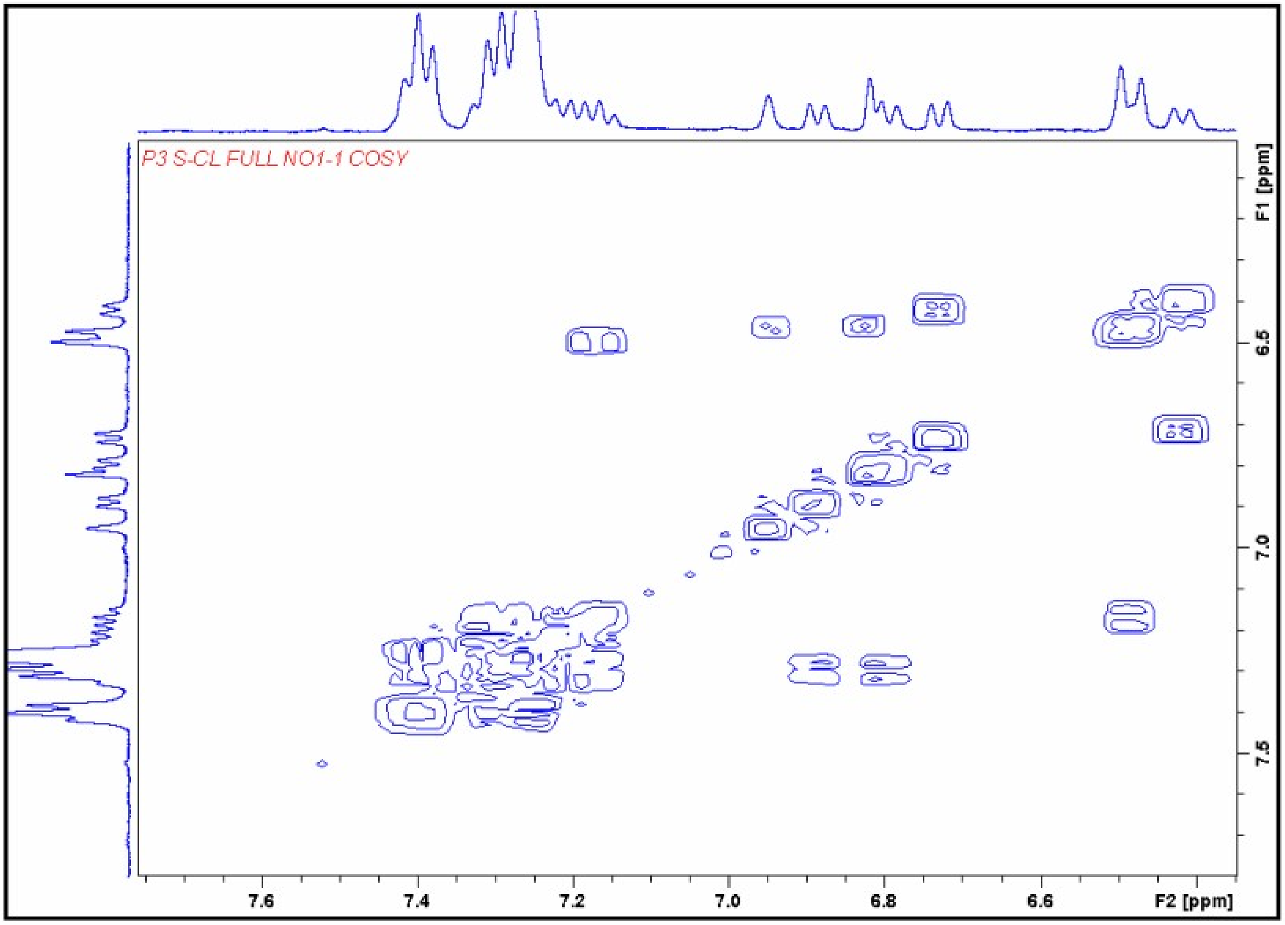

Figure S73: C COSY GPSW {^{1}H}($CDCl_3$, 400MHz) spectrum of compound (73) Scheme 17 (EXPANSION).

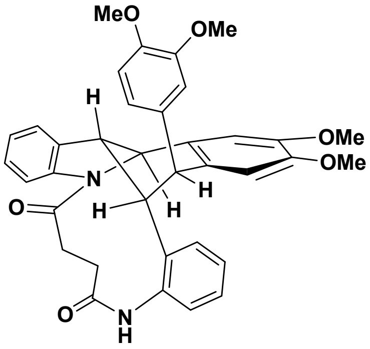

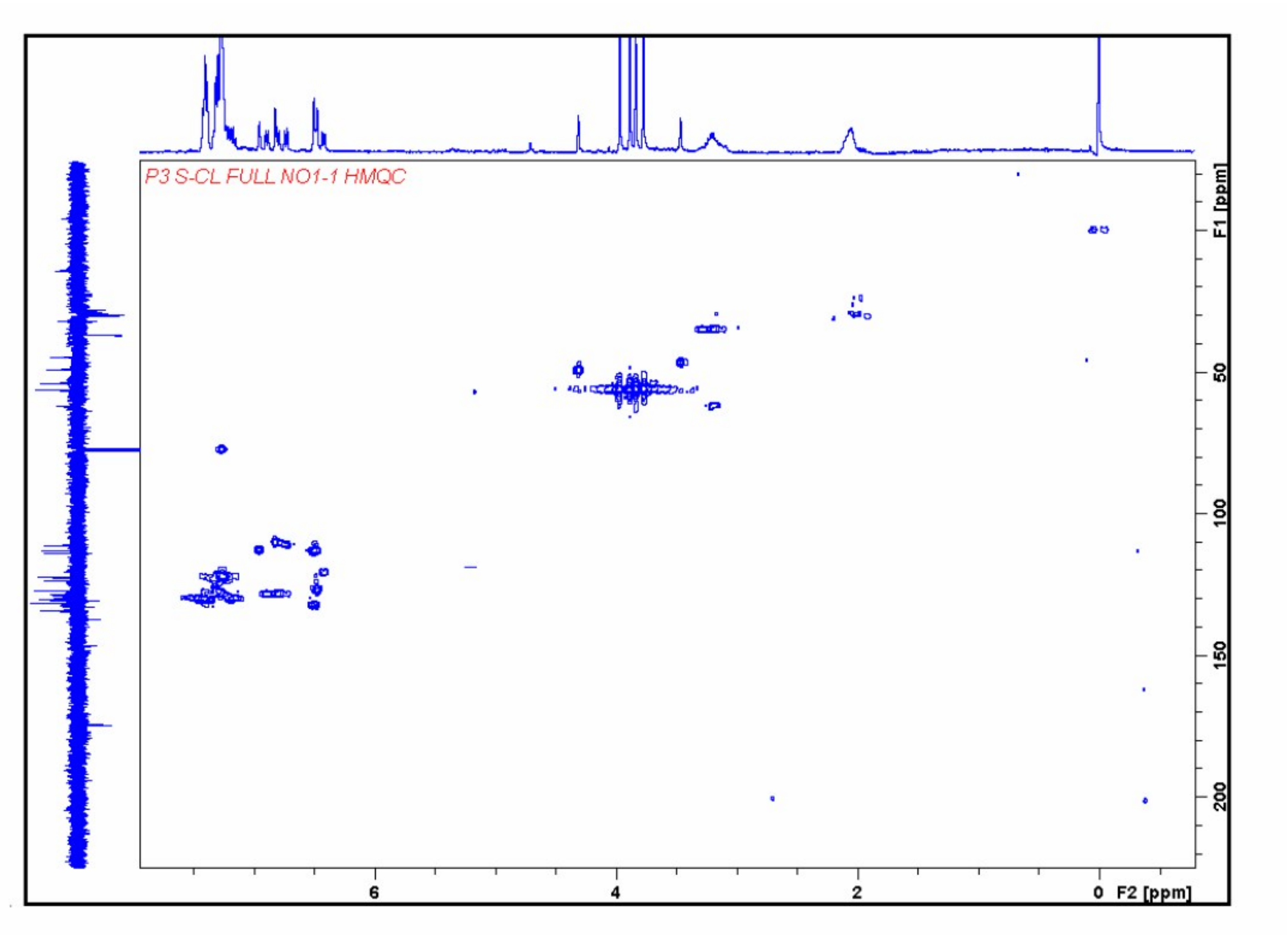

Figure S74: C HMQC GP {^{1}H-^{13}C}($CDCl_3$, 400MHz) spectrum of compound compund (73) Scheme 17.

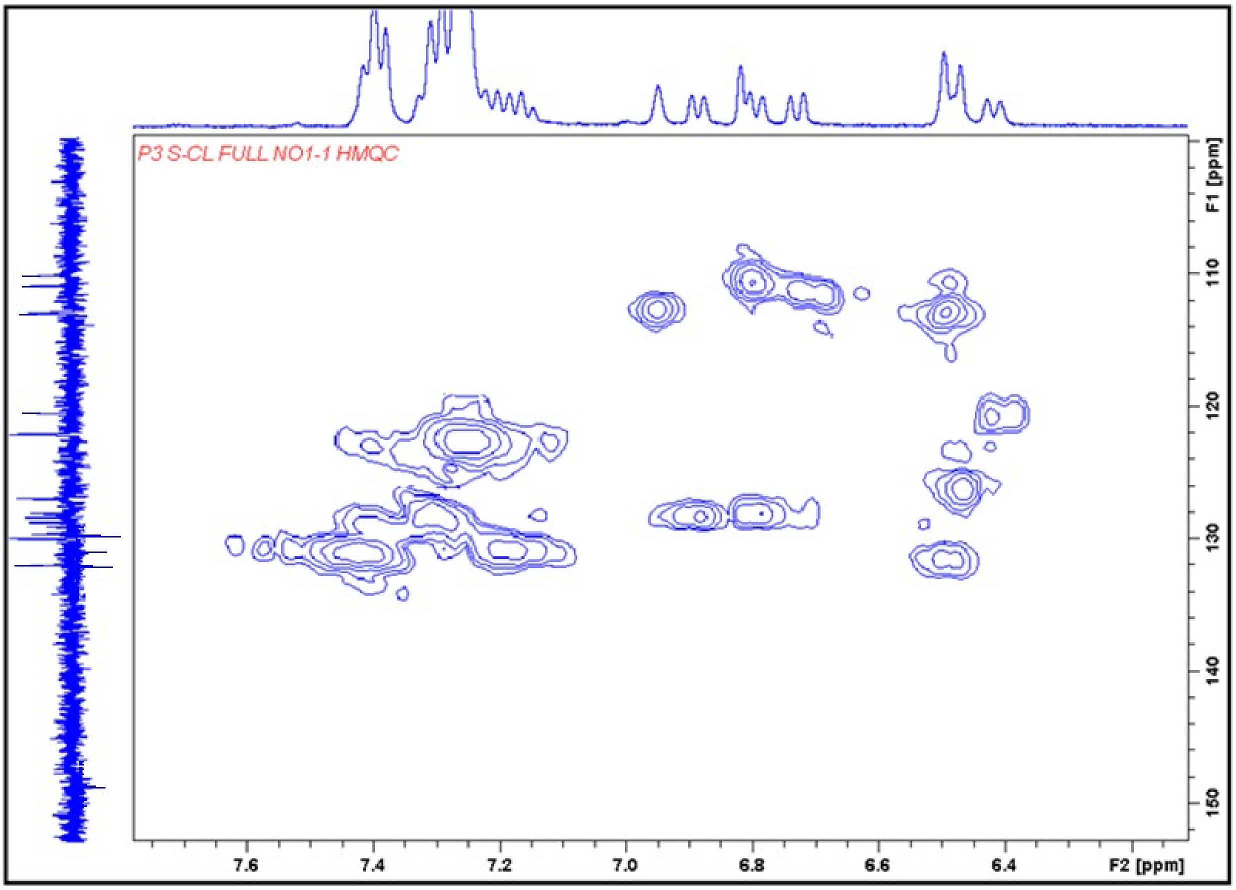

Figure S75: C HMQC GP {^{1}H-^{13}C}($CDCl_3$, 400MHz) s spectrum of compound compund (73) Scheme 17

(EXPANSION).

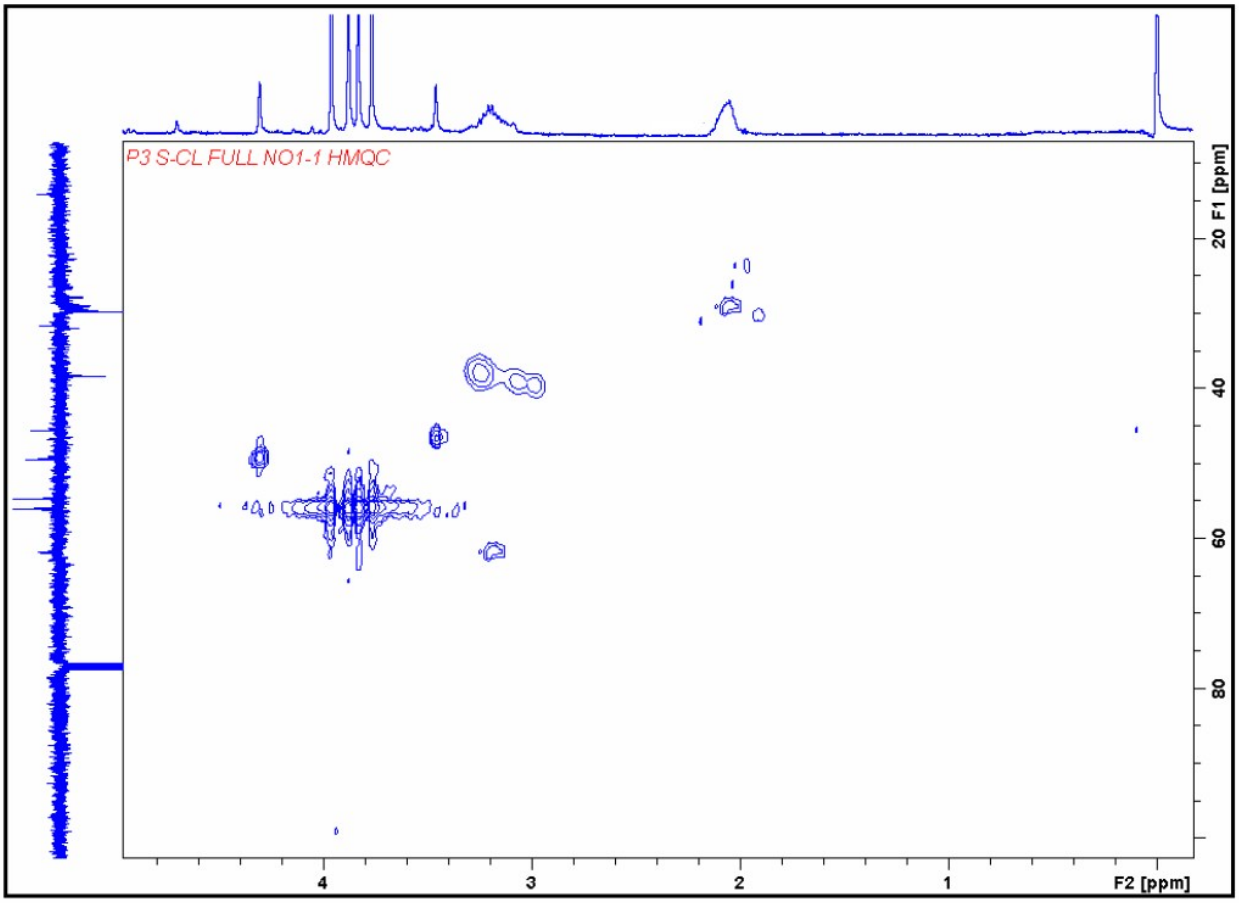

Figure S76: C HMQC GP {^{1}H-^{13}C}($CDCl_3$, 400MHz) spectrum of compound compund (73) Scheme 17 (EXPANSION).

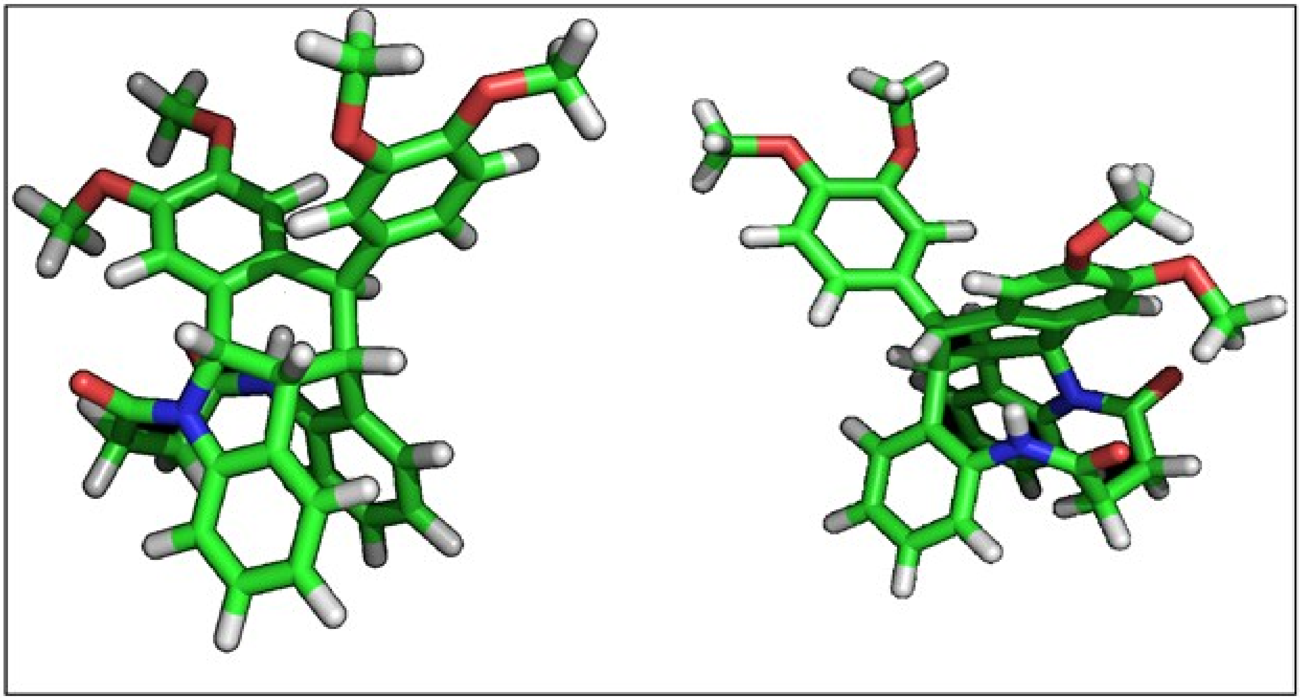

Figure S77 : Two views of the Chem 3D of compund **73**.

**Data File**	3 4 MEO NEG.d	**Sample Name**	3 4 MEO NEG
**Sample Type**	Sample	**Position**	P1-A2
**Instrument Name**	Instrument 1	**User Name**	
**Acq Method**	MARYAM SADAT NEG.m	**Acquired Time**	02-Aug-17 5:15:58 PM
**IRM Calibration Status**	Success	**DA Method**	default.m
**Comment**			

**Sample Group**		**Info.**
**Acquisition SW Version**	6200 series TOF/6500 series Q-TOF B.05.01 (B5125.3)	

Compound Table

Compound Label	Data File	RT	Mass	Abund	Name	Formula	Tgt Mass	Diff (ppm)	MFG Formula	MFG Diff (ppm)	DB Formula
Cpd 1: C16 H17 N O2		2.278	255.32	5790		C16 H17 N O2	255.1259	0.94	C16 H17 N O2		C16 H17 N O2

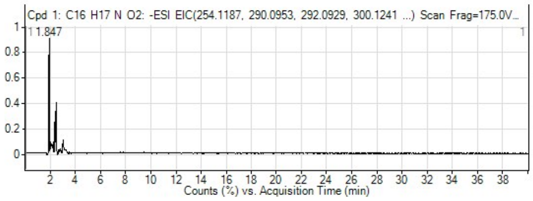

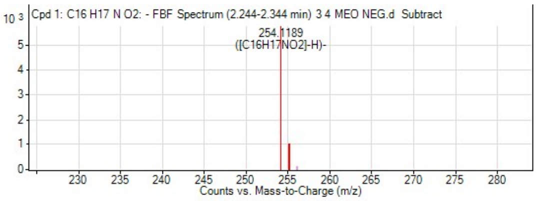

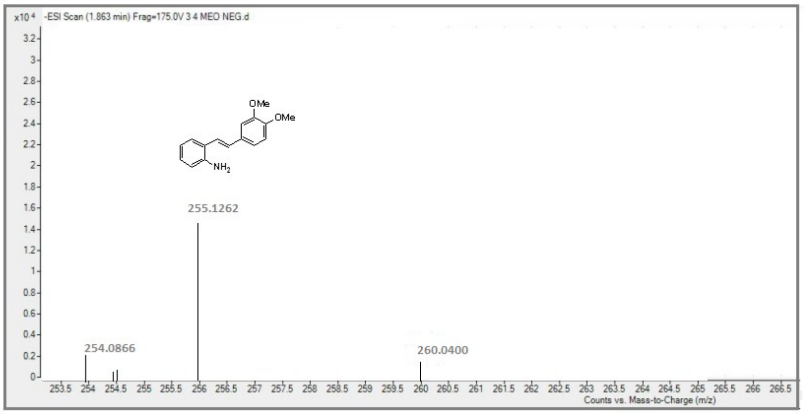

MS Spectrum Peak List

m/z	*Calc m/z*	Diff(ppm)	z	Abund	Formula	Ion
254.1189			1	5790.48	C16H17NO2	(M-H)-
255.322			1	567.57	C16H17NO2	(M-H)-

Figure S78: Mass information of compound (E)-2-(3,4-dimethoxystyryl)aniline.

**Data File**	3 4 MEO M Cl NEG.d	**Sample Name**	3 4 MEO M Cl NEG
**Sample Type**	Sample	**Position**	P1-A6
**Instrument Name**	Instrument 1	**User Name**	
**Acq Method**	MARYAM SADAT NEG.m	**Acquired Time**	03-Aug-17 8:38:30 AM
**IRM Calibration Status**	Success	**DA Method**	default.m
**Comment**			

**Sample Group**		**Info.**
**Acquisition SW Version**	6200 series TOF/6500 series Q-TOF B.05.01 (B5125.3)	

Compound Table

Compound Label	Data File	RT	Mass	Abund	Name	Formula	Tgt Mass	Diff (ppm)	MFG Formula	MFG Diff (ppm)	DB Formula
Cpd 1: C35 H34 N2 O6		2.308	578.2418	838747		C35 H34 N2 O6	578.2417	0.28	C35 H34 N2 O6		C35 H34 N2 O6

Compound Label	Name	*m/z*	RT	Algorithm	Mass
Cpd 1: C35 H34 N2 O6		577.2348	2.308	Find By Formula	578.2418

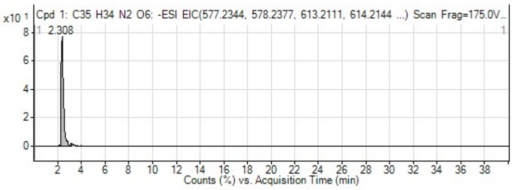

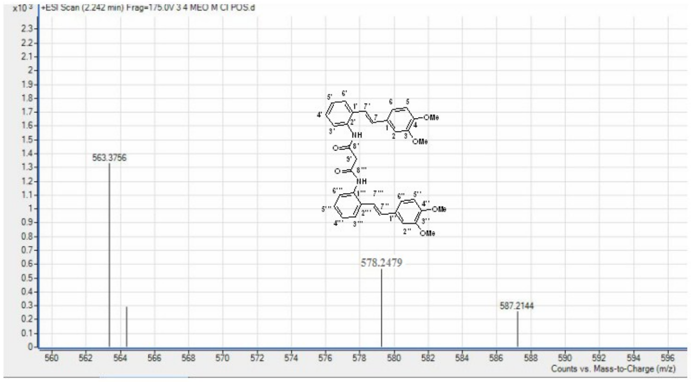

MS Zoomed Spectrum

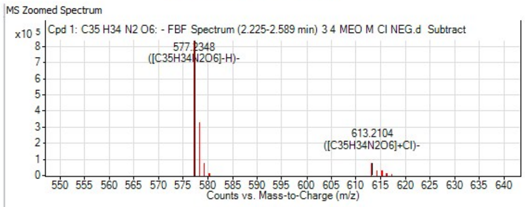

MS Spectrum Peak List

m/z	*Calc m/z*	Diff(ppm)	z	Abund	Formula	Ion
577.2348			1	838747.06	C35H34N2O6	(M-H)-
578.2378			1	324719.91	C35H34N2O6	(M-H)-
579.24			1	48982.52	C35H34N2O6	(M-H)-
580.2418			1	2976.33	C35H34N2O6	(M-H)-
613.2104			1	78116.98	C35H34N2O6	(M+Cl)-
614.213			1	18991.65	C35H34N2O6	(M+Cl)-
615.2085			1	17427.04	C35H34N2O6	(M+Cl)-
616.2099			1	3095.16	C35H34N2O6	(M+Cl)-
617.2111			1	352.83	C35H34N2O6	(M+Cl)-

--- End Of Report ---

Figure S79:Mass spectral data for compound bis (2-(E))-3,4-dimethoxystyryl)phenyl) malonamide.

**Data File**	3-4 MEO S Cl POS.d	**Sample Name**	3-4 MEO S Cl POS
**Sample Type**	Sample	**Position**	P1-A2
**Instrument Name**	Instrument 1	**User Name**	
**Acq Method**	MARYAM SADAT POS.m	**Acquired Time**	12-Jul-17 11:39:36 AM
**IRM Calibration Status**	Success	**DA Method**	default.m
**Comment**			

**Sample Group**		**Info.**
**Acquisition SW Version**	6200 series TOF/6500 series Q-TOF B.05.01 (B5125.3)	

Compound Table

Compound Label	Data File	RT	Mass	Abund	Name	Formula	Tgt Mass	Diff (ppm)	MFG Formula	MFG Diff (ppm)	DB Formula
Cpd 2: C36 H36 N2 O6		1.594	592.2557	1605		C36 H36 N2 O6	592.2573	-2.75	C36 H36 N2 O6		C36 H36 N2 O6

Compound Label	Name	*m/z*	RT	Algorithm	Mass
Cpd 2: C36 H36 N2 O6		593.2631	1.594	Find By Formula	592.2557

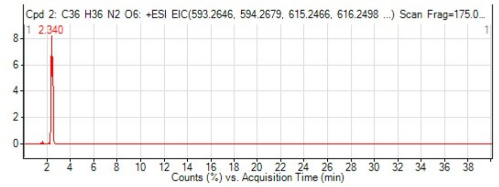

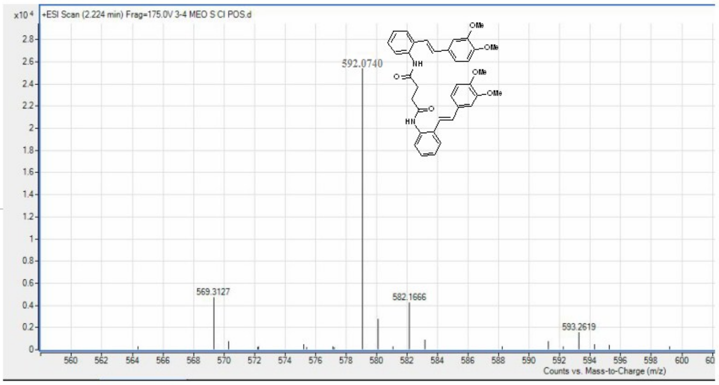

MS Zoomed Spectrum

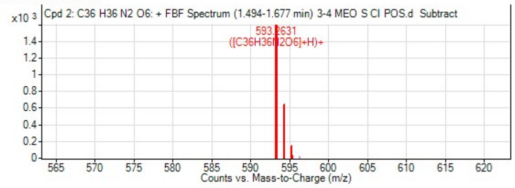

MS Spectrum Peak List

m/z	*Calc m/z*	Diff(ppm)	z	Abund	Formula	Ion
593.2631			1	1605.02	C36H36N2O6	(M+H)+
594.2658			1	370.72	C36H36N2O6	(M+H)+
595.2686			1	45.67	C36H36N2O6	(M+H)+

Figure S80: Mass spectral data for compound bis(2((E)-(3,4-dimethoxystyryl)phenyl)succinamide.

**Data File**	3 4 MEO GCl NEG.d	**Sample Name**	3 4 MEO GCl NEG
**Sample Type**	Sample	**Position**	P1-A5
**Instrument Name**	Instrument 1	**User Name**	
**Acq Method**	MARYAM SADAT NEG.m	**Acquired Time**	02-Aug-17 7:47:54 PM
**IRM Calibration Status**	Success	**DA Method**	default.m
**Comment**			

**Sample Group**		**Info.**
**Acquisition SW Version**	6200 series TOF/6500 series Q-TOF B.05.01 (B5125.3)	

Compound Table

Compound Label	Data File	RT	Mass	Abund	Name	Formula	Tgt Mass	Diff (ppm)	MFG Formula	MFG Diff (ppm)	DB Formula
Cpd 1: C37 H38 N2 O6		2.327	606.2723	226874		C37 H38 N2 O6	606.273	-1.08	C37 H38 N2 O6		C37 H38 N2 O6

Compound Label	Name	*m/z*	RT	Algorithm	Mass
Cpd 1: C37 H38 N2 O6		605.2653	2.327	Find By Formula	606.2723

MS Zoomed Spectrum

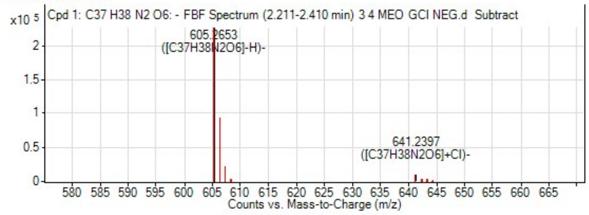

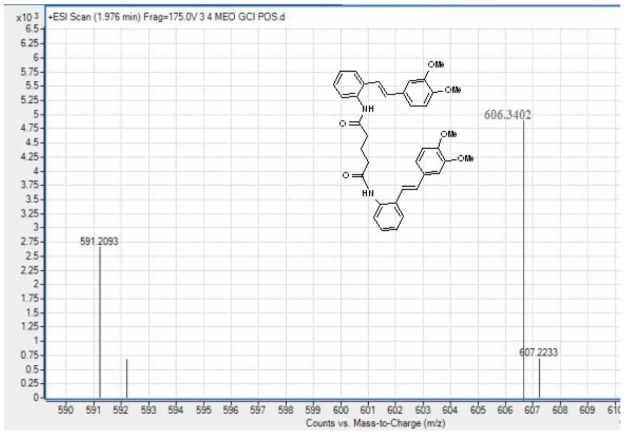

MS Spectrum Peak List

m/z	*Calc m/z*	Diff(ppm)	z	Abund	Formula	Ion
605.2653			1	226873.64	C37H38N2O6	(M-H)-
606.2682			1	76128.48	C37H38N2O6	(M-H)-
607.2704			1	7741.57	C37H38N2O6	(M-H)-
608.2718			1	517.17	C37H38N2O6	(M-H)-
641.2397			1	9114.51	C37H38N2O6	(M+Cl)-
642.2422			1	1962.98	C37H38N2O6	(M+Cl)-
643.2385			1	1741.4	C37H38N2O6	(M+Cl)-
644.2397			1	366.84	C37H38N2O6	(M+Cl)-

--- End Of Report ---

Figure S81: Mass spectral data for compound bis(2((E)-(3,4-dimethoxystyryl)phenyl) glutaramide.

**Data File**	3-4 MEO ADCl POS.d	**Sample Name**	3-4 MEO ADCl POS
**Sample Type**	Sample	**Position**	P1-A4
**Instrument Name**	Instrument 1	**User Name**	
**Acq Method**	MARYAM SADAT POS.m	**Acquired Time**	12-Jul-17 1:20:55 PM
**IRM Calibration Status**	Success	**DA Method**	default.m
**Comment**			

**Sample Group**		**Info.**
**Acquisition SW Version**	6200 series TOF/6500 series Q-TOF B.05.01 (B5125.3)	

Compound Table

Compound Label	Data File	RT	Mass	Abund	Name	Formula	Tgt Mass	Diff (ppm)	MFG Formula	MFG Diff (ppm)	DB Formula
Cpd 1: C38 H40 N2 O6		2.51	620.2889	157834		C38 H40 N2 O6	620.2886	0.48	C38 H40 N2 O6		C38 H40 N2 O6

Compound Label	Name	*m/z*	RT	Algorithm	Mass
Cpd 1: C38 H40 N2 O6		643.2785	2.51	Find By Formula	620.2889

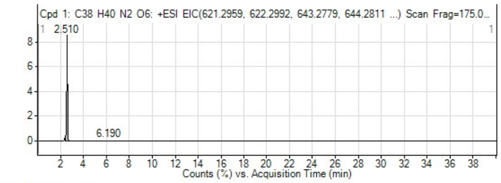

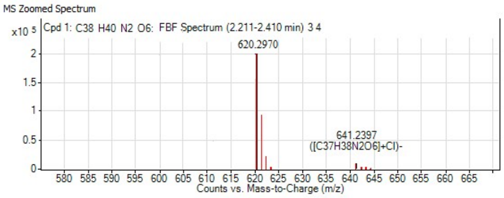

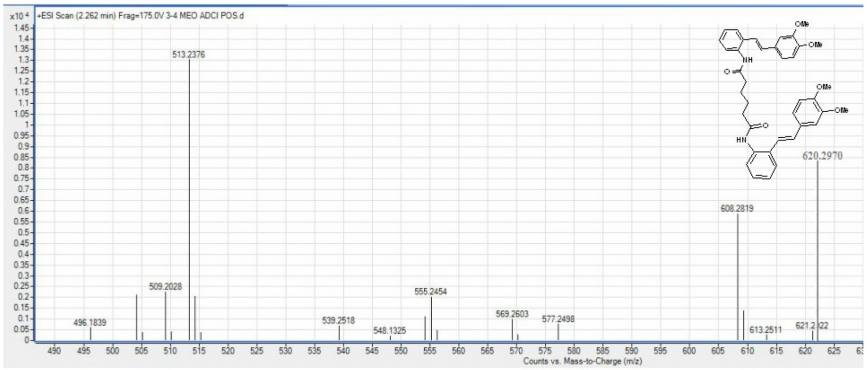

MS Spectrum Peak List

m/z	*Calc m/z*	Diff(ppm)	z	Abund	Formula	Ion
621.2958			1	47876.59	C38H40N2O6	(M+H)+
622.2986			1	12430.74	C38H40N2O6	(M+H)+
623.3005			1	1250.77	C38H40N2O6	(M+H)+
643.2785			1	157833.83	C38H40N2O6	(M+Na)+

Figure S82: Mass spectral data for bis(2((E)-(3,4-dimethoxystyryl)phenyl)adipamide.

**Data File**	P33 4 MEO S Cl POS.d	**Sample Name**	P33 4 MEO S Cl POS
**Sample Type**	Sample	**Position**	P1-A2
**Instrument Name**	Instrument 1	**User Name**	
**Acq Method**	MARYAM SADAT POS.m	**Acquired Time**	28-Sep-17 11:03:34 AM
**IRM Calibration Status**	Success	**DA Method**	default.m
**Comment**			

**Sample Group**		**Info.**
**Acquisition SW**	6200 series TOF/6500 series	
**Version**	Q-TOF B.05.01 (B5125.3)	

Compound Label	Name	*m/z*	RT	Algorithm	Mass
Cpd 274: Cer(d18:0/18:0)	**Cer(d18:0/18:0)**	**590.24**	**5.753**	**Find by Molecular Feature**	**590.24**

Compound Chromatograms

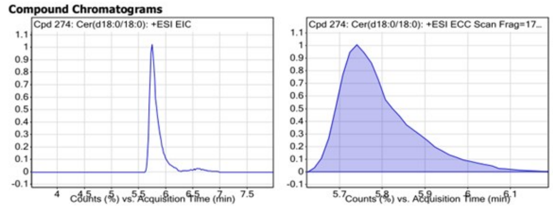

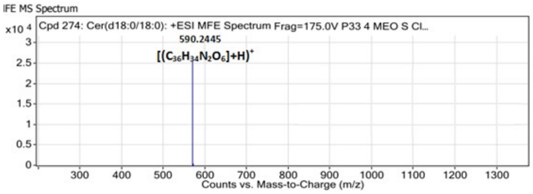

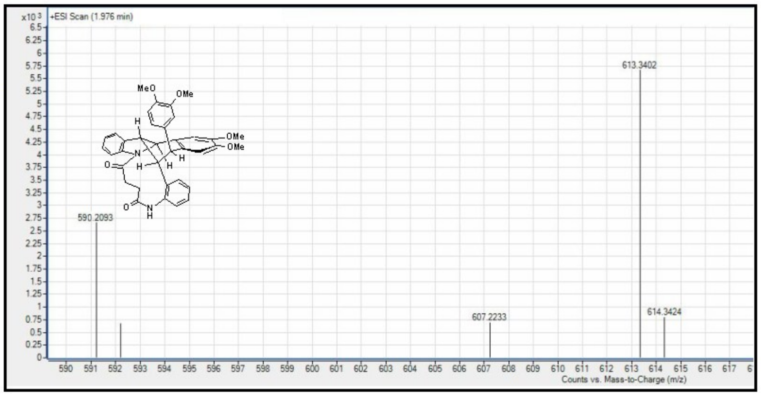

MS Spectrum Peak List

m/z	*Calc m/z*	Diff(ppm)	z	Abund	Formula	Ion
590.24			1	31679.46	C36 H34 N2 O6	(M+H)+
591.25			1	6497.74	C36 H34 N2 O6	(M+H)+
592.25			1	679.08	C36 H34 N2 O6	(M+H)+

MS Spectrum

Figure S83: Mass spectral data for compound (73) Scheme 17.
